# A Comprehensive Structural and Functional Analysis of *Saccharomyces* Killer Toxins

**DOI:** 10.3390/toxins18050235

**Published:** 2026-05-20

**Authors:** Jack W. Creagh, Lily L. Givens, David C. Reetz, Sarah A. Coss, Rodolfo Bizarria, Siti Aisyah Alias, Mohammed Rizman-Idid, Jagdish S. Patel, Andre Rodrigues, F. Marty Ytreberg, Paul A. Rowley

**Affiliations:** 1 Department of Biological Sciences, University of Idaho, Moscow, ID 83844, USA; jackcreagh2016@gmail.com (J.W.C.); daviddalo2012@gmail.com (D.C.R.);; 2 Department of Pharmaceutical Sciences, School of Pharmaceutical Sciences of Ribeirão Preto, University of São Paulo, Ribeirão Preto 14040, Brazil; 3 Department of General and Applied Biology, Institute of Biosciences, São Paulo State University (UNESP), Rio Claro 13506, Brazil; 4 Institute of Ocean and Earth Sciences, C308, Institute of Advanced Studies Building, Universiti Malaya, Kuala Lumpur 50603, Malaysiarizman@um.edu.my (M.R.-I.); 5 Department of Chemical and Biological Engineering, University of Idaho, Moscow, ID 83844, USA; 6 Institute for Modeling Collaboration and Innovation, University of Idaho, Moscow, ID 83844, USA; 7 Department of Physics, University of Idaho, Moscow, ID 83844, USA

**Keywords:** killer toxin, yeast, *Saccharomyces*

## Abstract

Antifungal killer toxins are cytotoxic proteins that have the potential to combat the growing threat of fungi to human health and agriculture. A lack of empirical tertiary structures has limited understanding of their mechanisms of action and their ability to target pathogens. In this study, AlphaFold and molecular dynamics simulations were used to generate tertiary structure models of all canonical *Saccharomyces* killer toxins and to place them in the context of historical empirical data. These models enabled the prediction of functional domains and posttranslational modifications, including proteolytic cleavage sites and disulfide bonds. They also revealed unexpected homology between *Saccharomyces* killer toxins, suggesting that all but K28 are likely ionophores. Structural homology to the well-studied killer toxins K1 and K2 enabled the prediction of the antifungal and immunity mechanisms of K1L, K21, K45, K74, and KHS. The understudied killer toxins Klus, KHR, and K62 were found to have homology to bacterial and plant toxins, including members of the aerolysin family and antifungal lectins. These structural similarities provide clues for the mechanisms of killer toxin carbohydrate binding, oligomerization, and membrane attack. This modeling approach will help guide the continued use of the model yeast *S. cerevisiae* to study killer toxins in the context of the wealth of functional data gathered in the decades since their first discovery.

## 1. Introduction

### 1.1. Killer Toxin Diversity and Prevalence

Killer yeasts produce extracellular antifungal proteins, known as killer toxins, that inhibit the growth of competing yeasts and filamentous fungi. Such a broad definition has included carbohydrate-degrading enzymes (chitinases, glucanases, etc.), nucleases, and toxins that specifically attack fungal cell membranes or arrest the cell cycle. As killer toxins are proteins, they can be considered distinct from the inhibitory properties of small molecules and peptides produced by yeasts (e.g., alcohols, mating pheromones, chelators, etc.) [1,2,3]. The baker’s and brewer’s yeast *Saccharomyces cerevisiae* was the first fungal species identified to exhibit antifungal activity due to the production of killer toxins [4]. These fungi, termed killer yeasts, have since been identified in many genera across the Ascomycota and Basidiomycota and are found in diverse environments, in association with insects, plants, and soils (e.g., refs. [5,6,7,8,9,10,11,12,13,14,15,16,17,18,19]). Killer yeasts have also been repeatedly isolated from anthropogenic fermentations used for food and beverage production, as killer toxins can either protect against or cause spoilage (e.g., refs. [20,21,22,23,24,25]). Killer toxin production has been detected in 50% of *S. cerevisiae* strains, and these killer yeasts were more frequently identified in human clinical isolates and in winemaking strains [8].

The widespread distribution of killer yeasts is thought to be driven by niche competition in the environment. Killer toxin production can increase yeast fitness under optimal conditions for toxin activity (e.g., low pH) [26,27,28,29,30,31,32,33], enabling invasion into toxin-sensitive populations and aiding dispersal [16,33,34,35]. Exposure of yeasts to killer toxins drives the evolution of toxin resistance [36,37]. Toxin resistance is also frequently observed during screens for novel strains of killer yeasts, suggesting the evolution of antitoxin defense mechanisms and adaptation of cellular pathways and structures to prevent intoxication. For example, alterations in the configuration of *S. cerevisiae* cell wall mannans and beta-glucans confer killer toxin resistance by preventing toxin binding [37,38,39,40,41,42,43]. The killer toxin defense protein (Ktd1) also blocks K28 intoxication by mislocalizing the toxin to the vacuole [44,45]. Thus, the production of killer toxins influences microbial community composition by selecting for resistance and is likely driving the evolution of novel toxins in an ongoing genetic arms race between yeasts [16,46]. Indeed, signatures of positive selection have been detected in both *KTD1* and various killer toxins, in addition to the horizontal transfer, expansion, and duplication of killer toxin genes [45,47,48,49]. Such evolutionary processes could explain why killer yeasts tend to exhibit a narrow spectrum of antifungal activity limited to specific yeast strains.

### 1.2. The Application of Killer Toxin Yeasts Against Pathogens and Spoilage Organisms

Fungal pathogens are devastating wild animal and plant populations (e.g., refs. [50,51,52,53,54,55]). They also pose a growing threat to agricultural yields and human health [56,57,58,59], highlighting the critical need for innovative antifungal strategies. The ability of killer yeasts to suppress pathogenic and spoilage fungi has long garnered interest in their potential use in agriculture and medicine. Specifically, killer yeasts can inhibit the growth of a wide range of human pathogenic fungi, including the WHO priority species *Nakaseomyces glabratus* (syn. *Candida glabrata*) and *Candida albicans* [60,61,62,63,64,65]. Similar studies have identified killer yeasts that can inhibit plant pathogens and spoilage fungi [66,67,68,69,70,71,72]. This has led to the application of killer yeasts onto plants, fruits, and silage, to prevent disease and spoilage. Killer toxins have also been used to develop genetically modified, disease-resistant wheat, tobacco, and maize [73,74,75]. In field trials, genetically modified maize expressing the killer toxin KP4 gained significant protection from a disease caused by smut fungi [76]. Recently, a killer yeast (*Suhomyces pyralidae*, LEVEL2 SALVA™) has been developed by the yeast supplier Lallemand to protect wine against spoilage yeasts of the genus *Brettanomyces* [77]. Killer yeasts can also remediate spoilage by diastatic strains of *S. cerevisiae* in brewing [78].

Killer toxins are unlikely to be toxic to humans because they are prevalent in strains of *S. cerevisiae* used in food and beverage production and are inactive at physiological temperatures and pH (Table 1). Additional safety studies suggest that they are also non-toxic to cultured mammalian cells, likely because they target fungal-specific cell-surface receptors and cell wall components (e.g., mannans, chitin, and glucans) [63,76,79]. However, killer toxins are diverse; a small number can target organisms beyond fungi, indicating a broader spectrum of cytotoxicity [80,81,82,83]. These findings underscore the potential of killer toxins as novel antifungals, offering possible future solutions to address the rising challenges posed by fungal pathogens and spoilers, but with the possibility that some could have negative consequences for human, animal, and plant health [84,85,86].

### 1.3. Killer Toxins of Saccharomyces Yeasts

Killer toxins from the *Saccharomyces* genus of yeasts are among the most well-studied, due to the powerful genetic tools that have been developed for use in the model yeast *S. cerevisiae*. The first killer toxin discovered, produced by *S. cerevisiae*, was named K1 [4]. Attempts to characterize the K1 gene discovered that it was inherited cytoplasmically in a non-Mendelian fashion [87]. The nucleic acids responsible for the K1 killer phenotype were subsequently determined to be double-stranded RNAs (dsRNAs) [88]. In many *S. cerevisiae* killer yeast strains, “M” (Medium; ~1500 bp) dsRNAs are associated with toxin production as they encode killer toxin genes and have historically been referred to as killer viruses or satellite viruses, but are now classified by the International Committee on Taxonomy of Viruses (ICTV) as dsRNA satellites [89]. M dsRNAs are always associated with “L” (Large; ~4600 bp) dsRNAs [90,91,92,93]. L dsRNAs were identified as viral genomes from the family *Totiviridae* that encode proteins enabling the replication and packaging of both L and M dsRNAs into viral particles [91]. Surveys of *S. cerevisiae* killer yeasts have revealed a significant correlation between the presence of L and M and the killer phenotype [8]. Advances in nucleic acid sequencing technology have identified different types of M-encoded killer toxins in *Saccharomyces* yeasts, including K1, K2, and Klus from *S. cerevisiae* [94,95,96], as well as K1L, K21, K66, K45, K62, and K74 from *Saccharomyces paradoxus* (Table 1) [40,47,97]. K28 has been identified in both *S. cerevisiae* and *S. paradoxus* [97]. Killer toxin-encoding M satellites are also present in other yeasts of the Ascomycetes [98,99] and Basidiomycetes [100,101,102].

Despite the prevalence of toxin-encoding M dsRNA satellites in *Saccharomyces* yeasts, they appear relatively rare in killer yeasts outside of the Saccharomycotina. While several examples of killer toxins encoded by double-stranded linear DNA plasmids exist [103], many killer toxins are genome-encoded. For example, the killer toxins KHR and KHS are found in the genomes of most *S. cerevisiae* strains, as well as other *Saccharomyces* and non-*Saccharomyces* species [8,104,105,106,107]. Genome-encoded homologs of the M satellite-encoded killer toxins Klus and K62 have also been identified in *S. cerevisiae* [49,94]. Whole-genome sequencing techniques have enabled the discovery of hundreds of putative killer toxins based on primary sequence homology to known killer toxins. One prominent example is the killer toxin KP4 produced by the Basidiomycete yeast *Mycosarcoma maydis* (syn. *Ustilago maydis*), which has hundreds of genome-encoded homologs in fungi and non-vascular plants [82,108,109,110]. The large numbers of putative killer toxins in the KP4 family and other groups of genome-encoded killer toxins have provided evidence for horizontal gene transfer, rapid evolution, and gene expansion [45,47,48,49]. The widespread distribution and diversification of killer toxin homologs underscore their importance in fungal ecology and evolution, suggesting they play multifaceted roles in competition, defense, and niche adaptation across diverse taxa. However, given the large and expanding number of these genes, it is a significant challenge to elucidate whether they are toxins or if they have other functional roles.

### 1.4. A Lack of Tertiary Structure Models of Killer Toxins

The lack of knowledge regarding the antifungal mechanisms of killer toxins is partly due to a paucity of tertiary structure models. To date, only six empirical structural models of fungal killer toxins have been determined, including those from *Millerozyma farinosa* (Salt Mediated Killer Toxin, SMKT), *Mycosarcoma maydis* (Killer Protein 4 (KP4), and Killer Protein 6 (KP6)), *Zymoseptoria tritici* (Zt-KP6-1 and Zt-KP4-1), and *Williopsis mrakii* (Williopsis mrakii Killer Toxin (WmKT)) (Table S1). These killer toxins are all small proteins (<223 amino acids) and, except for WmKT, have structural similarity based on a typical alpha/beta sandwich. Experimental determination of the tertiary structures of *Saccharomyces* killer toxins has proved challenging due to the sometimes complex workflows required for the purification of native toxins, low levels of expression, and toxicity to yeast when overexpressed [111,112,113,114,115,116,117]. Powerful recombinant expression systems that have been developed for bacteria have not been utilized due to the requirement for eukaryotic-specific posttranslational modification of killer toxins (i.e., protease cleavage, disulfide bond formation, glycosylation). Recombinant systems for killer toxin expression by yeasts have been developed, but none have been successfully applied to solving tertiary structures [118,119,120].

Recent advances in artificial intelligence-based protein structure prediction have enabled the generation of three-dimensional models directly from primary amino acid sequences. The most well-known example, AlphaFold, has revolutionized the field by employing deep neural architectures to infer high-confidence structural models without requiring a close homologous template [121]. Subsequently, molecular dynamics (MD) simulations are often employed to evaluate the conformational stability of these models and to probe their biophysical properties and atomic-level interactions. In contrast, classical structure prediction tools such as MODELLER, PHYRE, and PSIPRED rely on homology modeling or threading against empirically determined structures with shared sequence similarity [122,123,124]. Although such methods have been applied to generate structural models of killer toxins, their accuracy is fundamentally constrained by the limited availability of experimentally determined structures [82]. Accordingly, integrating cutting-edge artificial intelligence with MD simulations offers a powerful approach to infer the structures of killer toxins, their maturation, and potential mechanisms of action that were previously unattainable. Leveraging these new techniques is the central motivation for the manuscript, as these models will serve as a useful framework for future empirical investigations and for categorizing the thousands of putative protein toxins found in other organisms.

### 1.5. Manuscript Organization by Killer Toxin Families

This manuscript provides a comprehensive review of current knowledge of *Saccharomyces* killer toxin function and places it in the context of tertiary structural models generated by AlphaFold and refined with MD simulations. The manuscript is organized first by identifying sequence homology between known killer toxins and unknown proteins in publicly available sequence archives. This approach grouped the known toxins into six families based on primary, secondary, and tertiary structure analysis, which are then discussed in subsequent sections of the manuscript.

## 2. Results and Discussion

### 2.1. Classification of Killer Toxin Families by Primary Sequence Homology

**Figure 1:**
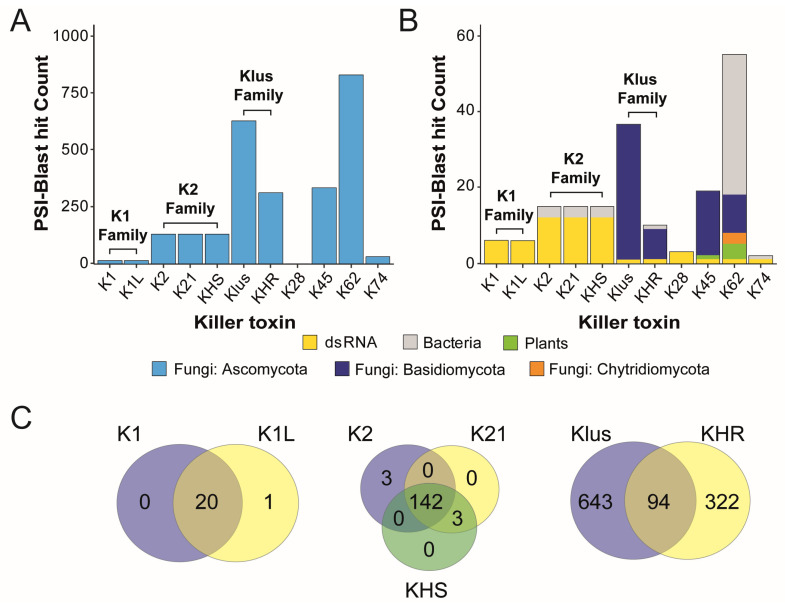
Sequence homologs of *Saccharomyces* killer toxins are abundant in fungi but are also found in plants and bacteria. (**A**) Identification of sequence homologs of 11 *Saccharomyces* killer toxins in the Ascomycota using the amino acid sequences of each canonical toxin to query the NCBI database using PSI-BLAST. Killer toxin families were identified based on overlap in sequence homologs between killer toxins K1/K1L (K1 family), K2/K21/KHS (K2 family), and Klus/KHR (Klus family). (**B**) Homologs of *Saccharomyces* killer toxins have been identified in fungi of the Basidiomycota and Chytridiomycota, bacteria, and plants. Killer toxins identified as being encoded on dsRNAs are also indicated. (**C**) Venn diagrams illustrating the overlap between sequence homologs of K1/K1L, K2/K21/KHS, and Klus/KHR.

This Initial analysis of *Saccharomyces* killer toxins was based on primary sequence similarity to more completely define the homology noted in prior publications [40,47,107]. To analyze the sequence similarity between *Saccharomyces* killer toxins and identify killer toxin homologs, the amino acid sequences of K1, K1L, K2, K21, K28, K45, K62, K74, KHR, KHS, and Klus were used as queries for position-specific iterated BLAST (PSI-BLAST) (Figure S1 and Table S2). A total of 4437 potential homologs were identified. Sequences were filtered to exclude those shorter than 75% or longer than 150% of the query sequence, removing 1703 candidates likely representing non-functional genes or proteins with possible functional divergence from killer toxins (Table S2). After filtering, sequence homologs were identified for all canonical killer toxins, except for K28, which yielded only three nearly identical sequences from different strains of *S. cerevisiae*. Across all of the identified homologs, 96% were identified in fungi, of which 93.4% were from the Ascomycota and 2.60% from the Basidiomycota and Chytridiomycota. The majority of all the identified homologs (35.4%) were in the subphylum Saccharomycotina (Figure 1A). Other killer toxin homologs were identified in bacteria (1.8%) and plants (0.2%) (Figure 1B). Of the 48 bacterial proteins identified, 37 were homologs of K62, with the remainder similar to K45 (*n* = 9), K74 (*n* = 1), and KHR (*n* = 1). Killer toxin homologs in plants were confined to K62 (*n* = 2) and K45 (*n* = 1). Finding killer toxin homologs encoded in the genomes of a wide diversity of organisms supports their horizontal transfer between species and ongoing gene diversification and expansion.

PSI-BLAST of K1 and K1L confirmed their sequence homology and identified that the majority of homologs were from Saccharomycotina yeasts and previously named “K1-like Killer Toxin” (*KKT*) genes [47]. The 21 homologs identified by K1 and K1L showed almost perfect overlap and were collectively named the K1 family (Figure 1C). The overlap in PSI-BLAST results across other *Saccharomyces* killer toxins enabled the identification of two additional toxin families: K2/K21/KHS (K2 family) and Klus/KHR (Klus family) (Figure 1C). The sequence homology between K2 and KHS has been previously reported in other yeasts of the Saccharomycotina (e.g., *Vanderwaltozyma polyspora*) [107]. Sequence homology searches using K2, K21, and KHS identified 150 unique proteins, with 97.9% of the homologs shared across all three killer toxins (Figure 1C). The Klus family has the second-largest number of homologs (after K62), with 871 proteins and an overlap of 94 proteins between Klus and KHR. PSI-BLAST analysis of the three other *Saccharomyces* killer toxins (K62, K45, and K74) identified lists of unique homologs and were named after the canonical killer toxins (Figure 1B,C).

With the establishment of primary sequence homology among *Saccharomyces* killer toxins, each killer toxin family was compared based on its predicted secondary and tertiary structures as well as posttranslational modifications, such as protease cleavage and disulfide bonding. This integrated approach was intended to strengthen confidence in the classification of killer toxin families and to detect deeper homology, both between killer toxin families and with other experimentally determined protein structures.

### 2.2. Molecular Modeling of Saccharomyces Killer Toxins

To determine structural similarities among the known *Saccharomyces* killer toxins, secondary and tertiary structures were predicted using AlphaFold2 [121]. AlphaFold2 estimated the confidence of each tertiary structure by predicted Local Distance Difference Test (pLDDT) for each residue of each killer toxin. Scores can range from the lowest (0) to the highest confidence (100). Values greater than 80.0 are considered of high confidence and most often correlate with regions of secondary structure, while lower scores indicate less confident structural predictions and disordered regions between elements of secondary structure. The average AlphaFold2 pLDDT scores for all killer toxin models ranged from 35.0 to 82.0, with K62 exhibiting the highest confidence and K28 and K74 the lowest (Figure S2). The majority of the killer toxin models (K1, K1L, K2, K21, Klus, KHR, and KHS) showed moderate overall confidence, with average pLDDT scores ranging from 51.0 to 68.0 (Figure S3 and Table S3). Despite relatively low global confidence, all models exhibited well-defined secondary structure elements with high local confidence, reflected by an average pLDDT maximum of 89.1.

For each killer toxin, the highest average confidence structures generated by AlphaFold2 were subjected to a 1 μs MD simulation to model behavior in a solvated environment and to improve the quality of the predicted structures; these were used for further functional analysis. Root mean squared deviation (RMSD) values were used to assess structural stability over time by comparing the movement of polypeptide backbone alpha carbons (Figure S4). Low RMSD values indicated little change from the initial AlphaFold2 tertiary structure during the MD simulation. In contrast, high RMSD values indicated movement of the tertiary and secondary structure away from the initial model. RMSD over time for most killer toxin tertiary models stabilized between 0.4 and 2.4 nm from the initial AlphaFold structure, indicating only small structural fluctuations (Figure S4). Most models stabilized after 100 to 200 ns, with K1, K2, KHS, and K45 stabilizing quickly and showing minimal RMSD movement for the remainder of the simulation. In contrast, KHR, Klus, and K62 each had a shift in RMSD during simulation, likely due to a conformational change. For K62, this shift occurred within 50 ns and stabilized after 100 ns, which was primarily due to N-terminal flexibility. For Klus and KHR, the shift occurred after 600 ns due to flexibility in the first 15 N-terminal residues and a large flexible loop (amino acids 111–161), respectively. For K62, Klus, and KHR, removal of these flexible regions resulted in more stable structures (Figure S4). The RMSD of K1L and K28 both increased throughout the simulation, indicating the general instability of these models, likely due to the lower-confidence AlphaFold models used prior to the MD simulation.

Ramachandran plots before and after MD were used to visualize improvements in phi and psi bond angles and to provide a graphical representation of favored and unfavored angles for all amino acid residues in each tertiary structure model (Figure S4) [125]. Folded proteins typically have most of their residues in favored regions of the plot, corresponding to secondary structural elements, such as α-helices and β-sheets. Overall, MD simulations refined the predicted killer toxin models by allowing backbone flexibility, resulting in more residues in favored regions and improved model quality. Overall, MD improved Ramachandran-favored residues by an average of 3.2% and reduced outliers by 2.3% (Figure S4 and Table S3).

### 2.3. The K1 Family

#### 2.3.1. K1 Family Introduction: Discovery and Early Characterization

K1 was the first killer toxin discovered and is one of the best understood due to decades of empirical investigation. K1 was also the first killer toxin to be identified as being encoded on a dsRNA satellite (M1) associated with a totivirus (L-A) [90]. Variants of K1 containing non-synonymous polymorphisms have also been reported to exhibit different antifungal activities [8,126]. Overall, K1 is a potent antifungal toxin that inhibits many yeasts and is particularly effective against the opportunistic human pathogen *N. glabratus* [61,63]. The toxin is heat-labile, with an optimal temperature for activity being ~25 °C. Like many killer toxins, K1 is most active in acidic conditions (Table 1).

K1L was identified as produced by *S. paradoxus* Y-63717, originally isolated from the exudate of an Asian aspen tree (*Populus davidiana*) in the eastern province of Khasan, Russia (see ARS Culture Collection (NRRL)). K1L is encoded on a dsRNA satellite, M1L, maintained by a totivirus (L-A-45), which also supports the replication of M45 (encoding K45) in a different East Asian strain of *S. paradoxus* [47]. The K1L toxin was identified as similar to K1 by predicted secondary structure and apparent domain organization, despite only 18% amino acid identity. K1L is more closely related to a group of genome-encoded homologs found across yeasts of the Saccharomycotina, which are active killer toxins when ectopically expressed by *S. cerevisiae* [47,127]. K1L has a unique spectrum of antifungal activity that is more similar to K1 but is less effective at inhibiting yeast growth than K1, is heat-labile, and has a pH optimum of 4.5 (Table 1) [47].

#### 2.3.2. K1 Family Introduction: Domain Organization and Maturation

**Figure 2:**
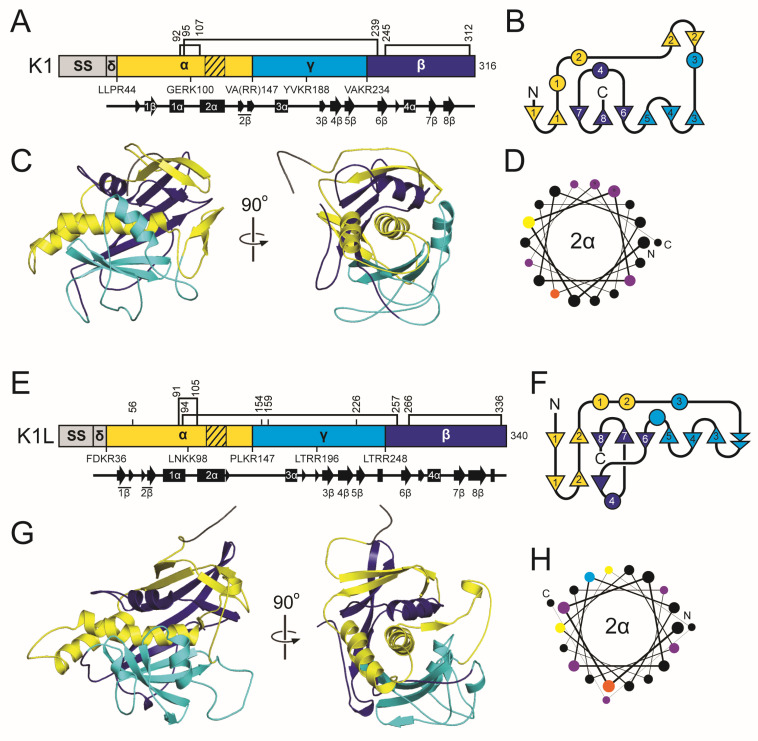
Secondary and tertiary structure models of K1 family killer toxins. (**A**,**E**) Domain diagrams of K1 and K1L ppTox indicate the proteolytic processing sites that define the delta, alpha, gamma, and beta domains. The positions of Kex protease cleavage sites are shown below the domain diagram, with the four amino acids preceding the cleavage point illustrated. The amino acids in parentheses indicate the dipeptide cleaved by Kex1 to create the mature C-terminus of the K1 alpha domain. Cysteine residues are indicated by lines and numbers above the domain diagram, with connections representing predicted disulfide bonds. Hatching represents amino acid sequences predicted to form transmembrane or pore-forming structures. Secondary structure, with numbered α-helices and β-strands, is represented by arrows and rectangles below the domain diagram, respectively. (**B**,**F**) Two-dimensional schematic of the relative secondary structure organization of K1 and K1L with α-helices represented as circles and β-strands as triangles (numbered as in the linear representation of the secondary structure). N = amino-terminus, C = carboxyl-terminus. (**C**,**G**) Killer toxin pTox tertiary structure models colored by domains as depicted in panels (**A**,**E**). (**D**,**H**) Helical wheel diagram of 2α helix, with hydrophobic (black), positively charged (yellow), negatively charged (blue), polar (purple), and aromatic (orange) amino acids. The line thickness between amino acids (from thick to thin) represents the sequence from the N-terminus to the C-terminus.

K1 is divided into four domains: delta (amino acids 27–44), alpha (45–147), gamma (148–234), and beta (235–316) (Figure 2 and Table S4). These domains are defined by sites of proteolytic processing during maturation of the 35 kDa (316 amino acids) primary translation product of K1, known as the preprocessed toxin (ppTox) [128]. To enter the secretory pathway, ppTox is exported to the endoplasmic reticulum (ER), where a signal sequence is cleaved after residue A26, forming the protoxin (pTox) [129,130]. The pTox is glycosylated, although mutation of residues targeted for this modification has no appreciable effect on the killer phenotype [131]. The K1 sequence contains six cysteine residues with a predicted interdomain disulfide bond between C92 and C239 of the alpha and beta domains, respectively (Table S5) [132]. Additional intradomain disulfide bonds are predicted between C95-C107 and C248-C312 [132]. The glycosylation and crosslinking of pTox results in a ~43 kDa pTox. Export of pTox to the Golgi is dependent on proteins of the secretory pathway, as blocking Golgi trafficking prevents further maturation of K1 by proteolytic processing [133]. Cleavage of K1 pTox occurs by the action of the Kex2 endopeptidase after basic residues R44, R149, and R233 [134,135]. A third potential Kex2 cleavage site is located after R188 in the gamma domain, and mutation of this site reduces K1 toxicity but not immunity [136]. Kex1 carboxypeptidase cleaves before R148 to remove an arginine dipeptide, creating the mature C-terminal end of the alpha domain [134,137]. Therefore, the mature 20.6 kDa K1 is a disulfide-linked heterodimer of processed alpha (11.1 kDa) and beta (9.5 kDa) domains linked by a single disulfide bond.

K1L and its homologs have a secondary structure organization similar to that of K1, including delta (27–36), alpha (37–147), gamma (148–248), and beta (249–340) domains, and a similar pattern of six cysteine residues split evenly between the alpha and beta domains (Figure 2 and Tables S4 and S5). K1L has longer delta, alpha, gamma, and beta domains compared to K1 (by 2, 6, 16, and 10 amino acids, respectively) (Table S4) [47,127]. Kex1 and Kex2 are also both required for the production of active K1L homologs [127]. The predicted alpha domain of K1L and its homologs is also cytotoxic when expressed by *S. cerevisiae*, consistent with the K1 alpha domain functioning as the toxin domain [115,127]. Overall, these empirical data support the close functional and structural relationship between K1, K1L, and their sequence homologs.

#### 2.3.3. The K1 Family Introduction: Antifungal Activities

The K1 alpha/beta heterodimer is an ionophoric toxin that increases the permeability of the yeast plasma membrane, leading to cell death [138,139]. The mechanism of intoxication by K1 begins with the toxin binding to the cell wall [140]. Both domains of mature K1 (alpha and beta) have been implicated in cell wall binding [141]. Fractionation of the yeast cell wall has identified that 1,6-β-D-glucan is the primary cell wall receptor for K1 [142,143]. Manipulation of 1,6-β-D-glucan content alters susceptibility to K1, with depletion and overexpression leading to decreased or increased sensitivity to K1 intoxication, respectively [38]. After cell wall binding, K1 attacks the plasma membrane, which is dependent on the Kre1 glycosylphosphatidylinositol (GPI) anchored protein [144,145]. Kre1 is thought to be the K1 membrane receptor, and its deletion confers high levels of resistance to both cells and spheroplasts [140,144,145,146,147]. A region of the C-terminus of Kre1 can bind K1 in vitro and is both necessary and sufficient for the intoxication of spheroplasts, but is insufficient for the intoxication of whole cells [145].

The increased permeability of the cell membrane after K1 intoxication is thought to result from the formation of voltage-independent ion channels that cause energy-independent potassium ion efflux and, potentially, hydrogen ion influx [148,149]. An alternative hypothesis for the observed ion leakage during K1 intoxication is the activation of the Tok1 potassium channel [150]. However, the role of Tok1 in K1 sensitivity has been questioned [38,145,151]. Disruption of critical electrochemical gradients leads to cell death via different pathways, depending on the K1 concentration [152,153]. The alpha domain appears to be solely responsible for intoxication, as its overexpression alone causes a suicidal phenotype independent of the Kre1 membrane receptor [115]. The primary structure of K1 alpha has been shown to contain hydrophobic α-helical regions, supporting a mechanism of membrane attack. Evidence for K1 oligomerization, consistent with channel formation, comes from observations of K1 assembling into large complexes or aggregates, as well as into soluble octamers [112,154,155].

#### 2.3.4. The K1 Family Introduction: Immunity

The expression of ppTox K1 is necessary and sufficient for the self-protection of K1 killer yeasts against attack from exogenous K1 killer toxin. K1 ppTox immunity depends on export to the ER, but the signal sequence is not directly involved in immunity [156]. Early studies were able to decouple immunity from K1 toxicity by isolating inactive K1 mutants that provide immunity [112,135,136,157]. Similarly, yeast strains lacking functional *KEX1* or *KEX2* do not produce mature K1 but remain immune to exogenous K1 [133]. Mutation of the beta domain or its deletion is also dispensable for K1 immunity, as is the majority of the gamma domain [156]. Moreover, C-terminal truncations of K1 identified that the minimal immunity domain consists of delta and alpha with 31 amino acids of gamma [136]. Mutagenesis further narrowed the minimal immunity region to the latter half of alpha and the N-terminus of gamma and identified that amino acids C95 and C107 are essential for toxicity and immunity [156]. Current working models of immunity predict that partially processed pTox (with alpha and gamma linked) prevents K1 intoxication at the cell surface by sequestering the Kre1 membrane receptor or mature K1 [136,156]. These models are supported by the detection of pTox mutants that provide K1 immunity outside of the cell, but the exact mechanism remains to be determined.

#### 2.3.5. The K1 Family: Molecular Modeling Results

The tertiary structure models of K1 and K1L reveal a shared architecture centered on a globular one-layer alpha/beta sandwich fold that encapsulates the central α-helix, 2α (Figure 2). In K1, 2α is predicted to participate in membrane interaction and pore formation, consistent with previously mapped hydrophobic regions of the alpha domain [135,141,156]. In both the K1 and K1L models, 2α is buried in a pocket formed primarily by antiparallel β-sheets, with support from α-helices of the surrounding alpha, gamma, and beta domains. In K1, 2α is nested in a pocket of approximately 1698.0 Å^2^, shaped by three discontinuous β-strands and sheets (1β, 3–6β, and 7–8β), burying 83.8% of the helix (Table S6). In K1L, 2α is enclosed by a similar assembly of antiparallel β-strands from the alpha (1–2β), gamma (3–5β), and beta domains (6–8β) that buries 90.9% of the surface area of the helix (2186.8 Å^2^) (Table S6). The K1L alpha domain also forms a continuous β-sheet with strands 1–2β, in contrast to the discontinuous configuration of the analogous β-strands in K1.

The K1 and K1L models predict both interdomain and intradomain disulfide bonds. In K1, a disulfide bond between C95 (alpha domain) and C239 (beta domain) likely forms before Kex cleavage in the Golgi, stabilizing the heterodimer (Figure 2 and Figure S5). This prediction aligns with empirical data indicating that C239 is the only beta domain cysteine essential for the alpha-beta disulfide linkage, but assigns C95 as the alpha-domain cysteine instead of C92 [132]. The K1L model mirrors the K1 architecture with a predicted interdomain disulfide bond between C94 and C257 (Figure 2 and Figure S5). Intradomain disulfides are also predicted in both toxins, such as C92–C107 in the alpha domain and C245–C312 in the beta domain of K1, which could contribute to structural rigidity and functional properties, including immunity and cell binding [132].

Both structural models are consistent with the known posttranslational maturation pathway of K1-family toxins, in which the pTox is processed in the Golgi by Kex1 and Kex2 proteases (Table S4). These cleavage sites occur at dibasic sites on solvent-exposed loops between domains, regions that are clearly accessible in tertiary structure models of K1 and K1L. Additional Kex cleavage sites are also located in the alpha and gamma domains on exposed linkers between 1α and 2α, and 3α and 3β. The internal cleavage of gamma appears to be important for K1 toxicity, but the functional relevance of the additional processing of alpha is unclear. A similar arrangement is observed in K1L, where the surface loops align with predicted Kex cleavage sites that define domain boundaries.

**Figure 3:**
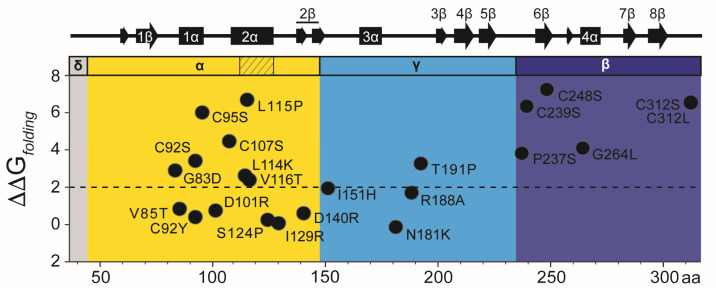
FoldX analysis of K1 mutations identified in previous empirical studies. FoldX mutagenesis of published point mutations positioned relative to the alpha, gamma, and beta domains and secondary structure of K1. The cutoff for mutations that are predicted to disrupt protein structure is shown as a black dashed line (ΔΔG_folding_ ±2 kcal mol^−1^). K1 secondary structure with α-helices and β-strands is shown as rectangles and arrows above each domain.

There are 22 point mutations identified in previous studies that affect the toxicity, immunity, and cell wall-binding properties of K1 [132,136,141,156]. To determine whether these mutations caused functional defects due to loss of protein stability, FoldX was used to predict the changes in folding stability (ΔΔG_folding_) of the K1 tertiary structure model (Figure 3 and Table S7) [158]. The majority (15 of 22) were predicted to be destabilizing using a cutoff of ΔΔG_folding_ >2 kcal mol^−1^, and four are not surface-exposed (V116, S124, I151, C248) [159,160]. Most destabilizing mutations reduced toxin secretion (11/15) and all reduced antifungal activity against whole cells (15/15). Only two mutations predicted to be destabilizing retained >75% of wild-type activity and secretion (G264L and T191P).

All but one of the seven single cysteine mutants in K1 are predicted to be structurally unstable, which is consistent with their general loss-of-function (Figure 3 and Table S7) [132]. All cysteine mutations reduce K1 toxicity against whole cells, and only C248S in the beta domain was able to kill spheroplasts [132]. C92S in the alpha domain and all beta domain cysteine mutations retained functional immunity. Empirical data also show that cysteine mutations in the alpha domain (C92S, C92Y, C95S, and C107S) and beta domain (C239S) reduce the expression of extracellular pTox and/or mature K1 [132,136]. The instability of alpha domain mutants likely explains the loss of K1 alpha toxicity when expressed alone in *S. cerevisiae*. Conversely, K1 with single (C248S) or double (C248S and C312S) mutations in the beta domain are expressed at wild-type levels and capable of inhibiting the growth of spheroplasts but not whole cells. This indicates that the predicted misfolding of the K1 beta domain would generally prevent cell wall binding but not cell membrane attack. Therefore, these mutations appear not to cause additional misfolding of the alpha domain or loss of K1 expression, enabling membrane receptor binding and membrane permeabilization by the alpha domain.

Seven of the eight K1 mutations predicted to be stable (ΔΔG_folding_ <±2 kcal mol^−1^) resulted in defects in toxicity and/or immunity (V85T, D101R, S124P, I129R, D140R, N181K, and R188A) (Table S7). Six of these amino acids were predicted to be surface-exposed (V85T, D101R, I129R, D140R, N181K, and R188A), and three were positioned in regions of the protein lacking secondary structure (I129R, N181K, R188A). These mutations could therefore be important for defining regions of K1 that mediate cell recognition or the conformational changes required for toxicity (Figure 3). Specifically, residue D101 in the alpha domain is surface-exposed and positioned on the loop between α-helices 1α and 2α. The mutation D101R (ΔΔG_folding_ = 0.52 kcal mol^−1^) results in a loss of cell wall binding while retaining the ability to kill spheroplasts and confer immunity. This residue could define a surface contact point between the toxin and the yeast cell wall, which could be useful for defining K1 specificity.

### 2.4. The K2 Family

#### 2.4.1. K2 Family Introduction: Discovery and Early Characterization

K2 was the second killer toxin discovered after screening 964 yeasts from various genera in the National Collection of Yeast Cultures (NCYC) [5]. All K2 killer yeast strains originated from ale yeasts isolated from U.K. breweries, and K2 was identified as a toxin with a distinct activity and immunity profile [5]. Since their initial discovery, K2 killer yeasts have been repeatedly isolated from wineries and breweries [8,161,162,163]. Extraction of dsRNAs from K2 killer yeasts confirmed the presence of an M2 satellite. The dsRNA was required for K2 expression, as curing with cycloheximide or high temperatures led to loss of the M2 and killer phenotype [17]. The M2 genetic sequence identified the K2 open reading frame, which was confirmed to encode the K2 ppTox responsible for the observed antifungal and immunity phenotypes [96,164,165]. K2 exhibits optimal killing activity at pH 4.3 and between 20 and 25 °C [114]. The toxin has a broad spectrum of activity, inhibiting the growth of many species in the *Saccharomyces* genus as well as the human pathogen *N. glabratus* and diastatic brewing strains (Table 1) [5,63,78].

The K21-producing strain *S. paradoxus* T21.4 was originally isolated from oak trees in the U.K. [166]. The toxin produced by *the S. paradoxus* T21.4 was erroneously considered a K1 or K28 toxin [100,101] before being designated the unique toxin named K21 [97]. The toxin was confirmed to be encoded by an M satellite by dsRNA extraction and curing of the dsRNA satellite by growth at elevated temperature [10]. Sequencing of the M21 satellite found little nucleotide sequence homology to known yeast killer toxins but a similar organization to other dsRNA satellites [97]. Another dsRNA-encoded toxin, K66, with 92% amino acid identity to K21, was later identified in the *S. paradoxus* strain ALM–66 from the spontaneous fermentation of serviceberries [40]. The antifungal activities of K21 and K66 inhibit the same species of yeasts, and the immunity functions of both toxins are cross-protective (i.e., K21 protects against K21 and K66). K66 has an optimal antifungal activity at 20 °C and pH 4.8 (Table 1) [40].

First discovered in 1990, KHS (Killer of Heat-Sensitive) was identified in *S. cerevisiae* isolated from Japanese wineries [20]. The killer yeast was found to lack an M satellite, and the killer phenotype was resistant to curing by cycloheximide and elevated temperatures, suggesting a genome-encoded killer toxin [20]. The *KHS1* gene was initially identified from *S. cerevisiae* genomic libraries and mapped to the right arm of chromosome V [167]. This gene was cloned and expressed ectopically, confirming that it conferred the killer phenotype and its respective immunity functions [167]. Initial sequencing data contained errors, which were later corrected in subsequent studies [107]. Importantly, *KHS1* is absent from the reference genome of *S. cerevisiae*, but is present in most other strains, often with polymorphisms and premature stop codons [9,168]. Comprehensive screening of more than 1000 strains of *S. cerevisiae* confirmed the prevalence of killer toxin production that correlated with apparently functional *KHS1* [8,9]. The close similarity of some *KHS1* genes identified in *S. cerevisiae* and *S. paradoxus* supports introgression from *S. paradoxus* [9]. Homologs of *KHS1* have also been identified in the genomes of other yeast species within the Saccharomycotina [107].

The antifungal activities of KHS are consistent with other killer toxins, with an optimal activity at pH of 4.7 and ambient temperatures <30 °C (Table 1). However, the antifungal activity of KHS appears to be ineffective against many strains of *S. cerevisiae* and *S. paradoxus*, presumably due to the prevalence of *KHS1* and associated (but as of yet uncharacterized) immunity function. Thus, prior screens for killer toxins likely failed to recognize the widespread production of active KHS by *S. cerevisiae*. Indeed, the antifungal activities of KHS so far appear limited to the opportunistic pathogen *N. glabratus* and a single strain of *S. cerevisiae* [8,9,167]. Although little is known about the mechanism of action of KHS, its amino acid sequence homology to K2 and K21 suggests a similar ionophoric antifungal and immunity mechanisms.

#### 2.4.2. The K2 Family Introduction: Domain Organization and Maturation

**Figure 4:**
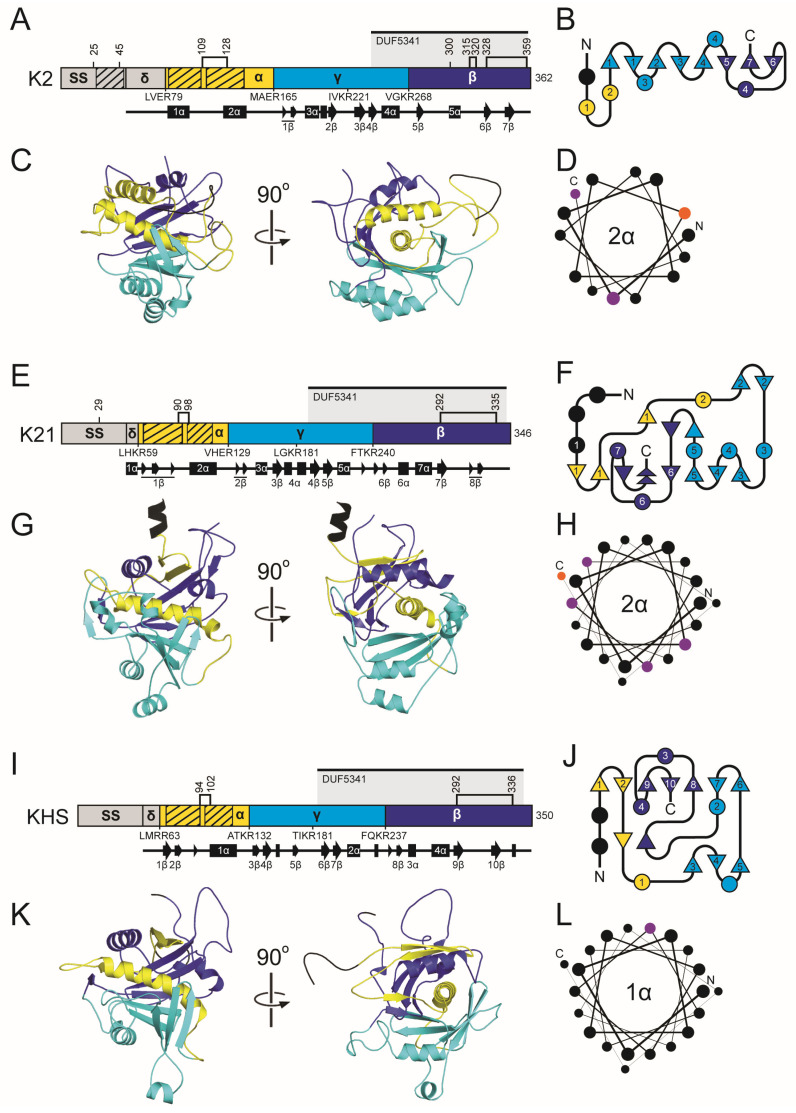
Secondary and tertiary structure models of K2 family killer toxins. (**A**,**E**,**I**) Domain diagrams of K2, K21, and KHS ppTox indicating sites of proteolytic processing that define the delta, alpha, gamma, and beta domains. Kex protease cleavage sites are indicated below the domain diagram, with the four amino acids preceding the cleavage point illustrated. Cysteine residues are shown by lines and numbers above the domain diagram, with connections between them representing predicted disulfide bonds. Hatching in the diagram represents amino acid sequences predicted to form transmembrane or pore-forming structures. Secondary structures, including numbered α-helices and β-strands, are represented below the domain diagram by arrows and rectangles, respectively. DUF5341 aligning region marked by a bar above the domain diagrams at amino acids 239–360 (K2), 190–343 (K21), and 185–344 (KHS). (**B**,**F**,**J**) Two-dimensional schematic of the relative secondary structure organization of K2, K21, and KHS, with α-helices represented as circles and β-strands as triangles (numbered as in the linear representation of the secondary structure). N = amino-terminus, C = carboxyl-terminus. (**C**,**G**,**K**) Tertiary structure models of K2, K21, and KHS pTox colored by domains as depicted in panels (**A**,**E**,**I**). (**D**,**H**,**L**) Helical wheel diagram of 2α helix, with hydrophobic (black), polar (purple), and aromatic (orange) amino acids. The line thickness between amino acids (from thick to thin) represents the progression of the sequence from the N-terminus to the C-terminus.

The K2 ppTox consists of 362 amino acids across four domains: delta (amino acids 55–79), alpha (80–165), gamma (166–268), and beta (269–362), in order from the N- to C-terminus (Figure 4) [169]. The primary transcription product is predicted to encode a 39 kDa protein [164]. The K2 ppTox is predicted to enter the secretory pathway, first being exported to the ER, where a signal sequence is cleaved. However, the signal sequence prediction tools SignalP and PSIPRED fail to recognize a canonical signal sequence in K2 (Figure 4 and Table S4), but the N-terminal prepro region possesses certain properties consistent with an *S. cerevisiae* signal sequence [170]. These include an N-terminal region with positively charged residues, followed by a hydrophobic α-helical region, and a recognition sequence for a signal peptidase. The difficulty in detecting this signal sequence in K2 is due to an additional ~30 residues before the predicted cleavage site, rather than the more typical five residues. In silico truncation of the K2 N-terminus results in the recognition of a signal sequence by prediction tools [170]. These analyses predicted that the signal peptidase cleavage site for K2 is somewhere after amino acid 54 [170].

Following K2 signal peptidase cleavage, pTox enters the ER, where disulfide linkages can form. K2 pTox in the Golgi is predicted to be cleaved by the Kex2 protease after R79, R165, R221, and R268, with likely additional processing by Kex1, resulting in a mature heterodimeric toxin (Figure 4 and Table S4) [164]. Loss of either or both Kex proteases reduces or eliminates K2 toxicity, respectively, without altering K2 immunity function [164]. Extensive mutagenesis supports a K2 domain organization similar to that of K1/K28, with a gamma domain that is internally cleaved and removed during toxin maturation [169]. This is evident in the gamma domain’s high mutational tolerance and in the observed decrease in function resulting from mutation of putative Kex cleavage sites [169]. Assuming processing at the non-canonical signal peptidase cleavage site and predicted Kex cleavage sites, K2 alpha and beta have theoretical molecular weights of ~8.7 kDa and ~10.5 kDa, respectively. Mature K2 appears to have an apparent molecular weight of ~21.5 kDa. However, the amino acid sequences at the termini of the mature K2 toxin domains are yet to be experimentally validated.

The K21 ppTox consists of a 346 amino acid protein that can be divided into four domains: delta (amino acids 41–59), alpha (60–129), gamma (130–240), and beta (241–346) in order from the N- to C-terminus (Figure 4 and Table S4). Domain boundaries are defined by dibasic and basic residue motifs for Kex cleavage that are positioned similarly to K2. PSIPRED predicts that the K21 signal sequence lies after amino acid 40, in line with the organization and posttranslational modification of K2. Moreover, K21 has a hydrophobic region between residues 23–39 that is typical of a signal sequence, but like K2, is positioned away from the N-terminus. K21 also has a region of positive charge with amino acids R9, R14, and K22 forming a conserved triad of residues before the hydrophobic region in the N-terminus. Although K21 has only four cysteine residues, their positions in the alpha and beta domains are similar to K2.

There has been no prior attempt to determine whether KHS is posttranslationally modified. However, due to its sequence homology with K2 and K21, it is predicted to have four domains: delta (37–63), alpha (64–132), gamma (133–237), and beta (238–350) (Figure 4 and Table S4). Unlike K2 and K21, all of the domain boundaries are defined by dibasic residue motifs that are likely cleavage sites for Kex proteases. The similar positioning of the KHS dibasic sites supports the functionality of non-canonical monobasic cleavage sites of K2 and K21. PSIPRED predicts that KHS has signal peptidase cleavage site after L36. Like K2 and K21, the signal sequence cleavage site is positioned away from the N-terminus due to the extension of the N-terminus before the pattern of positively charged residues (R20, R23, R26) and a hydrophobic domain (residues 27–46). The cysteine residues of KHS are positioned more similarly to K21 than compared to K2.

#### 2.4.3. The K2 Family Introduction: Antifungal Activities

Similar to K1, the K2 toxin is thought to be an ionophore. The mature heterodimeric toxin interacts with 1,6-β-D-glucan of the yeast cell wall as a primary receptor [171]. Enzymatic removal of the cell wall does not protect from K2 intoxication, indicating that K2 also interacts with the plasma membrane [172]. Moreover, the loss of Kre1 provides protection against K2 for whole cells and spheroplasts, and it has been suggested that, like K1, it is the secondary membrane receptor for K2 [172]. However, unlike K1, there has not been a direct measure of K2 interaction with Kre1. Exposure of yeast cells to K2 leads to cellular damage (as measured by lipophilic anion binding), which correlates to reduced respiration activity and lowered intracellular ATP levels, but without detectable ATP leakage seen during K1 intoxication [173]. Scanning electron microscopy of K2-intoxicated cells revealed shrinkage, loss of turgor, and surface cracks and pores, suggesting disruption of the cell wall and membrane [162,174]. Transmission electron microscopy also revealed disruption of the cell wall and an abnormal undulating morphology of the plasma membrane. Expression of the K2 alpha domain is also toxic to yeasts, suggesting that it is responsible for cytotoxicity, as is the case for the K1 alpha domain [170].

Genome-wide screens have identified hundreds of genes that influence K1, K2, and K21/K66 resistance or hypersensitivity [38,40,41]. Genes associated with resistance predominantly involve cell wall and plasma membrane structure, biogenesis, and mitochondrial function. Conversely, genes associated with hypersensitivity are primarily linked to stress signaling pathways and ion and pH homeostasis. These findings suggest that, while K2 and K21 share mechanistic similarities with K1, supporting their classification as ionophoric toxins, they also exhibit unique interactions with specific cellular components during intoxication.

Toxins of the K2 family may share further functional similarity due to the presence of a conserved Pfam Domain of Unknown Function (DUF5341) (Figure 4) [97]. This domain resides in the C-terminus of each K2 family toxin, beginning in the gamma domain and extending nearly the entire length of the beta domain. This configuration predicts that complete cleavage by Kex proteases of the K2 family of toxins would split the domain into two, with only the C-terminal portion present in the mature toxin. DUF5341 has 106 Uniprot entries, 82 of which are uncharacterized fungal proteins with unknown function in the genomes of Ascomycota fungi (Table S8). The remaining 24 entries include other members of the K2 family, proteins that appear to be associated with the fungal cell wall, and several YER187W and YGL262-like proteins, whose similarity to KHS and K2 has been previously documented [107]. The majority (76%) of DUF5341 domains are found in the C-terminus of small proteins (100–400 amino acids), similar to the organization found in the K2 family. Exactly half of the DUF5341-containing proteins also overlap with PSI-BLAST hits for the K2 family and are mostly uncharacterized. While the function of DUF5341 is unknown, homology to the beta domain of the K2 family may suggest a conserved carbohydrate-binding functionality across these proteins.

#### 2.4.4. The K2 Family Introduction: Immunity

Like the majority of killer toxins, K2 killer yeasts are immune to their own toxin [17]. Unlike K1 and K28, which require ppTox for functional immunity, the prepro region at the N-terminus of K2 is necessary and sufficient for immunity [164,170]. The prepro immunity function of K2 was first observed after the creation of a mutant that lacked K2 immunity but expressed an active toxin [164]. Moreover, yeast expressing this mutant K2 toxin grew well at pH 7 (K2 inactive), but were unhealthy at pH 4.5 (K2 active). A more recent study identified an N-terminal immunity peptide in the K2 prepro region that is released after signal sequence cleavage [170]. The mechanism by which the K2 prepro region functions as an immunity factor is not fully understood; however, due to its hydrophobicity, it may interact with cell membranes, similar to other small immunity peptides from bacteriocin immunity systems [175]. Localization of the K2 immunity domain to membranes could prevent pore formation, but other mechanisms are also plausible. Despite the low sequence similarity and identity of 31% and 10%, respectively, K21 and KHS have a similar organization in the N-terminal region, suggesting a similar immunity mechanism to K2.

#### 2.4.5. The K2 Family: Molecular Modeling Results

Superimposition of the predicted structures of the K2 family resulted in RMSD values of 5.8 Å (K2/K21), 3.9 Å (K2/KHS), and 1.1 Å (K21/KHS), revealing a shared globular organization centered around a conserved central α-helix surrounded asymmetrically by a series of discontinuous antiparallel β-strands. This one-layer alpha/beta sandwich architecture appears characteristic of *Saccharomyces* ionophoric toxins and is similar to the predicted structures of the K1 killer toxin family.

In K2, the central 2α helix is positioned within the alpha domain and is flanked by a total of eight β-strands: 1–2β (alpha domain), 3–4β (gamma domain), and 5–7β (beta domain). Helices from all three domains, including 1α, 3α, and 4α, contribute to wrapping the central helix and the burial of 1652.2 Å^2^ or 95.5% of the central 2α helix (Table S6). Notably, α-helices 5α and 6α lie on the opposite side of the β-sheet and do not appear to interact directly with 2α. Hydrophobicity and PSIPRED analyses suggest that α-helices 2α and 1α of the alpha domain may function as membrane-interacting or pore-forming structures, reinforcing the proposed mechanism of action for K2. The AlphaFold2 model of K2 is consistent with its known Golgi-mediated post-translational processing, as experimentally validated Kex cleavage sites are located on solvent-exposed loops that would facilitate proteolysis. Unlike K1, K2 does not appear to form interdomain disulfide bonds between the alpha and beta domains (Figure S5 and Table S5). Instead, intradomain disulfide bonds are predicted in the alpha domain linking the N-terminus of 2α to the C-terminus of 1α. A beta domain disulfide bond serves to link the K2 C-terminal tail to the penultimate β-strand (6β), reminiscent of K1 pTox. Recent studies support the functional significance of these cysteines in K2 activity [169].

The K21 structural model closely mirrors that of K2 but displays more disordered regions and less extensive secondary structure. Like K2, K21 has a central 2α helix (residues 97–119) predicted to be transmembrane based on hydrophobicity and secondary structure predictions. The central helix is embedded in a seven-stranded β-sheet composed of elements from all three domains: 1–3β (alpha domain), 4–5β (gamma domain), and 6–8β (beta domain) that bury a surface area of 1652.2 Å^2^ or 83.2% of the central helix (Table S6). Surrounding α-helices (5α, 6α, and 7α) interact with exposed regions of the 2α helix, while 3α and 6α interact with the solvent-exposed face of the gamma domain. An additional hydrophobic region with β-strands, located at residues 62–85 in the alpha domain, aligns with a similar α-helical region in K2, suggesting that it may contribute to membrane association. K21 is predicted to undergo Kex cleavage at exposed loops, and two intradomain disulfide bonds are formed in the alpha and beta domains, similar to K2 (Figure S5 and Table S5).

The KHS model shares the same core topology as K2 and K21, though with some structural differences. It features a central α-helix (1α) surrounded by nine antiparallel β-strands: 1–2β (alpha domain), 6–7β (gamma domain), and 8–10β (beta domain), burying a large area of 1831.8 Å^2^ or 89.2% of the surface area of 1α (Table S6). Like K2 and K21, the central α-helix of the KHS alpha domain is predicted to be transmembrane, being almost exclusively composed of hydrophobic residues (Figure 4L). A second transmembrane region is also present in the alpha domain, consisting of β-strands similar to K21 (amino acids 68–94). Helices 4α and 5α (from the alpha and beta domains) contact the exposed side of the central α-helix. Similar to K21, KHS shows greater structural disorder and loop flexibility than K2. Kex cleavage sites and disulfides are also positioned similarly to K21 and K2 (Figure S5 and Table S5).

### 2.5. The K45 Family

#### 2.5.1. The K45 Family Introduction: Discovery and Early Characterization

K45 is a poorly studied killer toxin produced by *S. paradoxus* N-45, which was isolated from the exudate of a Mongolian oak (*Quercus mongolica*) in Ternei City, Russia (see ARS Culture Collection (NRRL)) [166]. After an initial screen in 2013 that failed to identify N-45 as a killer yeast [10], an antifungal phenotype was observed in a later survey, which then mischaracterized the toxin as K1 [176]. A more detailed analysis of this killer yeast revealed a dsRNA satellite, designated M45, that showed little nucleotide homology to either M28 or M1, as determined by Northern blotting [97]. Further efforts to obtain the genetic sequence of M45 identified the killer toxin K45, but no other studies have characterized this toxin (Table 1).

#### 2.5.2. The K45 Family Introduction: Domain Organization and Maturation

**Figure 5:**
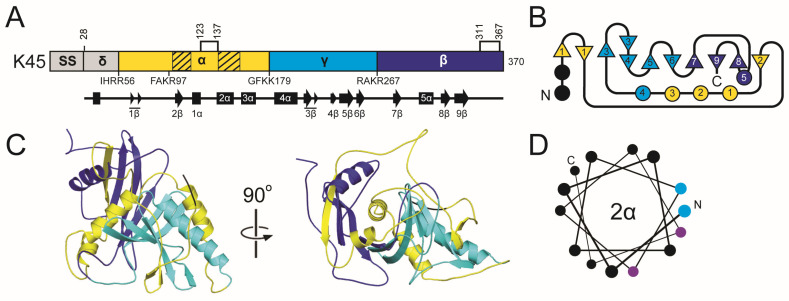
Secondary and tertiary structure models of K45 family killer toxins. (**A**) Domain diagrams of K45 ppTox indicating the sites of proteolytic processing that define the delta, alpha, gamma, and beta domains. The sites of Kex protease cleavage are numbered below the domain diagram, with the four amino acids prior to the cleavage point illustrated. Cysteine residues are indicated by lines and numbers above the domain diagram, with connections between them representing predicted disulfide bonds. Hatching in the diagram represents an amino acid sequence predicted to form transmembrane or pore-forming structures. Secondary structure, with numbered α-helices and β-strands, is represented by arrows and rectangles below the domain diagram, respectively. (**B**) Two-dimensional schematic of the relative secondary structure organization with α-helices represented as circles and β-strands as triangles (numbered as in the linear representation of the secondary structure). N = amino-terminus, C = carboxyl-terminus. (**C**) Tertiary structure model of killer toxin pTox colored by domains as depicted in panel (**A**). (**D**) Helical wheel diagram of 2α helix, with hydrophobic (black), negatively charged (blue), and polar (purple) amino acids. The line thickness between amino acids (from thick to thin) represents the progression of the sequence from the N-terminus to the C-terminus.

Overall, the domain organization of K45 is predicted to resemble that of other *Saccharomyces* killer toxins, with the order of delta (28–56), alpha (57–179), gamma (180–267), and beta (268–370). The boundaries between these domains are defined by putative Kex cleavage sites (Figure 5). An additional dibasic motif (K96 and R97), located near the center of the alpha domain, resembles dibasic sites found in the alpha domains of KHS and K1, but it has no known biological function. Although SignalP failed to identify a signal sequence cleavage site, PSIPRED predicted cleavage after amino acid 27. The sequence before the signal sequence cleavage site exhibits an organization typical of a *Saccharomyces* signal sequence, featuring positively charged amino acids and a hydrophobic region (Table S4). Similar to K1 and K2 toxin families, K45 contains a hydrophobic α-helix located in the C-terminal half of the alpha domain. Another hydrophobic region in the alpha domain includes 2β.

#### 2.5.3. The K45 Family: Molecular Modeling Results

K45 shares tertiary structure features with the K1 and K2 families, with a central α-helix (2α) surrounded by eight β-strands organized in an antiparallel arrangement: 2β (alpha domain), 3–6β (gamma domain), and 7–9β (beta domain). This extensive structure, along with two other α-helices (1α and 5α), buries 1158.1 Å^2^ of surface area or 89.2% the 2α helix (Table S6). The lone 5α helix of the beta domain is positioned antiparallel to the 2α helix and offset by ~20°. Compared to 2α, 5α is only partially wrapped by the central β-sheet, with a large portion of the structure being solvent-exposed. The hydrophobic 2β strand from the alpha domain is responsible for the majority of the interactions with 5α, as it is positioned at the end of the β-sheet region next to 8β. K45 has five cysteines, one of which is removed upon cleavage of the signal sequence, and the other four are predicted to form disulfide bonds in the alpha and beta domains. The organization of the cysteine and disulfide bonds is most similar to K2 family toxins (Figure S5 and Table S5). As with the K1 and K2 families, disulfides link the N-terminus of the central α-helix to the C-terminal end of the preceding secondary structure element (1α in K45) and pin the C-terminal tail to the beta domain.

### 2.6. The K74 Family

#### 2.6.1. The K74 Family Introduction: Discovery and Early Characterization

*S. paradoxus* strains Q74.4 and Y8.5 were both isolated from oak trees in the U.K. and identified as killer yeasts [10,176]. Despite the erroneous initial identification as K1, the novel spectrum of antifungal activity led to the discovery of K74 and the novel dsRNA satellite M74 [97,177]. Efforts to study the function of K74 have been aided by the expression of the toxin by laboratory strains of *S. cerevisiae* and the cytoduction of M74. Overexpression of K74 determined that the optimal conditions for the activity of K74 are pH 4.3 and 20 °C, which is consistent with other *Saccharomyces* killer toxins (Table 1) [177].

#### 2.6.2. The K74 Family Introduction: Domain Organization and Maturation

**Figure 6:**
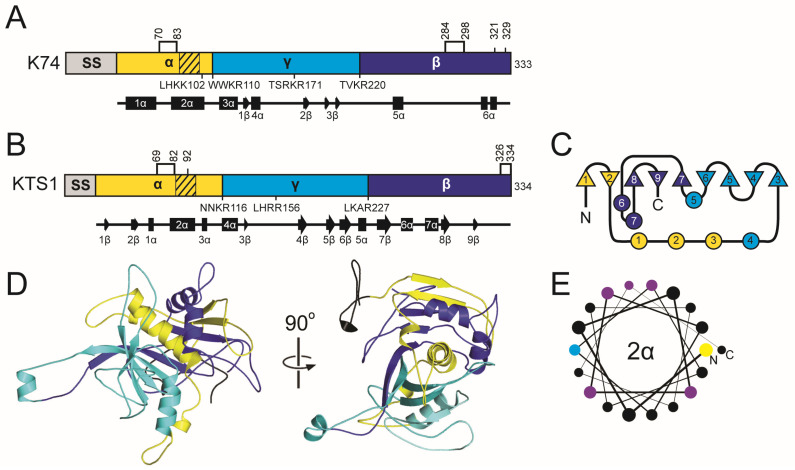
Secondary and tertiary structure models of K74 family killer toxins. Domain diagrams of (**A**) K74 and its sequence homolog (**B**) KTS1^Cmal^ ppTox, indicating the sites of proteolytic processing that define the delta, alpha, gamma, and beta domains. The sites of Kex protease cleavage are indicated below the domain diagram, with the four amino acids preceding the cleavage point illustrated. Cysteine residues are indicated by lines and numbers above the domain diagram, with connections between them representing predicted disulfide bonds. Hatching in the diagram represents amino acid sequences predicted to form transmembrane or pore-forming structures. Secondary structure, represented by numbered α-helices and β-strands, is illustrated by arrows and rectangles below the domain diagram, respectively. (**C**) Two-dimensional schematic of the relative secondary structure organization of KTS1^Cmal^ with α-helices represented as circles and β-strands as triangles. N = amino-terminus, C = carboxyl-terminus. (**D**) Tertiary structure model of killer toxin pTox colored by domains as depicted in panel (**B**). (**E**) Helical wheel diagram of 2α helix, with hydrophobic (black), positively charged (yellow), negatively charged (blue), and polar (purple) amino acids. The line thickness between amino acids (from thick to thin) represents the progression of the sequence from the N-terminus to the C-terminus.

All functional analyses of K74 have been reported by Rodriguez-Cousiño et al., who predicted proteolytic posttranslational modification due to the presence of dibasic motifs [97,177]. These predictions indicate that K74 is organized similarly to a typical killer toxin with alpha, beta, and gamma domains, but with a significantly longer beta domain and a shortened gamma domain compared to other *Saccharomyces* killer toxins (Table S4 and Figure 6). Expression of K74 in a *kex2∆* null strain caused the loss of mature K74 and the accumulation of glycosylated pTox [177]. Mutation of arginine residues (R110 and R220) at dibasic motifs that define the boundaries of alpha/gamma and gamma/beta domains, respectively, also prevented the processing of pTox to mature K74. Western blot analysis of K74 with reducing agents caused the release of a ~13 kDa C-terminal beta domain, suggesting that the mature K74 is a disulfide-linked heterodimer. Systematic mutation of all six cysteines of K74 revealed that they are required for the production of the mature toxin; however, it remains unresolved whether any cysteine pair (or pairs) is responsible for intramolecular crosslinking between the alpha and beta domains. The positioning of the cysteines in the alpha and beta domains is similar to that of the K1/K2/K45 killer toxin families.

#### 2.6.3. The K74 Family: Molecular Modeling Results

The confidence of the AlphaFold2 molecular model of K74 was the second lowest among the *Saccharomyces* killer toxins and had the highest proportion of unstructured loops (64.26%) (Figures S2 and S3, and Table S3). AlphaFold3 was used to model K74 but also yielded a low-confidence model and so we cannot be confident about the predicted structure of this toxin [178]. To create a more confident representative model of the K74 killer toxin family, 31 tertiary structure models were generated from all K74 homologs with an average sequence identity of 19.2% (SD 14.8%) (Figure 1). Of these models, 21 had high confidence scores, with pTM values greater than 0.7 (Table S9). These models contained putative Kex2 cleavage sites that divide the protein into a domain configuration similar to K1, with a central 2α helix from the alpha domain wrapped by β-strands from the alpha, beta, and gamma domains (Figure S6). The model from an uncharacterized K74 homolog from the saprophytic plant pathogen *Cadophora malorum* (named hereafter Killer Toxin Seventy four from *C. malorum*; KTS1^Cmal^) had the highest identity to K74, with a pTM score greater than 0.8. MD simulations indicated that the predicted structure was also stable (Figures S2 and S3, and Table S3). All confident structures of K74 homologs have structural similarity to KTS1^Cmal^, with an average RMSD of 2.06 Å (Table S9).

The tertiary structure model of KTS1^Cmal^ is a globular protein with an organization similar to that of other ionophoric killer toxins (delta, alpha, gamma, and beta) (Figure 6). The alpha and gamma domain boundary is a dibasic motif, whereas the boundary between gamma and beta is a non-canonical cleavage site (LKAR) (Table S4). Of the K74 homologs, 12 share this monobasic non-canonical gamma/beta cleavage site while 20 possess a dibasic cleavage site, suggesting that this cleavage site is biologically active. Monobasic sites are less common in killer toxins but have been confirmed in K1 at the delta/alpha boundary and in K28 between alpha/delta and alpha/gamma. Mutagenesis data also support the cleavage of monobasic sites in K2 [169]. A cleavage site in the middle of the gamma domain of KTS1^Cmal^ also demonstrates similar domain organization to K1, K2, and K28 and divides gamma into 39 and 71 amino acid peptides. All Kex2 cleavage sites of KTS1^Cmal^ are solvent exposed, with 2/3 on flexible loops in relatively the same positions as K74 (Table S4).

As in the molecular models of K1, K2, and K45 family killer toxins, the hydrophobic 2α helix of KTS1^Cmal^ is wrapped by a discontinuous antiparallel β-sheet composed of strands from all three domains. The alpha domain contributes β-strands 1–2β, gamma 4–7β, and beta 8–9β. Helices 1–4α interrupt the continuity of the β-sheet and separate 1–2β from 3–8β. The central hydrophobic α-helix 2α is also clamped by the beta domain α-helices 6α and 7α. As a result of these interactions, 87.3% (1721.2 Å^2^) of the surface area of 2α is buried (Table S6). The gamma domain interacts predominantly with the alpha domain, specifically the 2α helix (Figure 6). The C-terminus of helix 1α is connected to 2α by a loop that is predicted to be stabilized by the disulfide bond C69-C82, which is a common configuration in the K1, K2, and K45 families of killer toxins (Table S5). Overall, the cysteine residues are predicted to form intradomain disulfide bonds in the alpha (C69-C82) and beta (C326-C334) domains. The predicted disulfide bonds in KTS1^Cmal^ and other homologs are generally inconsistent with the disulfide-linked heterodimer that was predicted for K74 [177]. Moreover, there is only one K74 homolog, from the filamentous fungus *Glarea lozoyensis*, with a predicted alpha-beta interdomain crosslink, but this is due to a unique cysteine configuration compared to K74. Together, these data suggest that the alpha/beta heterodimer of K74 and its homologs is likely held together by non-covalent interactions, as is predicted for K2, K21, KHS, and K45. Although these data indicate that the structure of K74 may be an outlier within a larger family of K74-like proteins, homologous proteins provide a useful framework for further investigation of the structure and function of K74.

### 2.7. Mechanistic Insights into the K1, K2, K45, and K74 Families

Molecular modeling of the K1, K2, K45, and K74 killer toxin families indicated a shared structural organization. Primary and secondary structure analysis has previously revealed homology between several *Saccharomyces* killer toxins, suggesting conserved tertiary structures between K1L/K1 and K2/KHS [47,107]. Other similarities between K1, K2, and K21 toxins have been based on commonalities in cellular proteins required for killer toxin resistance and their apparent mechanism of action, i.e., cell permeabilization and ion efflux. A four-domain organization is also a common feature among these killer toxins, as indicated by the conserved positioning of monobasic and dibasic motifs recognized by Kex proteases. However, a shared structural layout and nomenclature do not directly determine a common antifungal mechanism, as toxins such as K28 and K1 have the same domain naming convention and domain order (delta, alpha, gamma, and beta) but kill cells by fundamentally different mechanisms.

**Figure 7:**
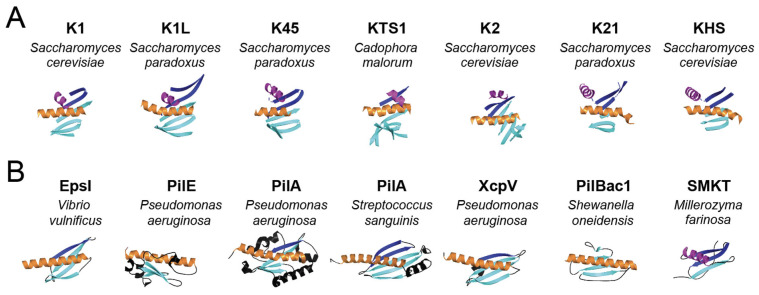
A conserved core motif of the K1 superfamily shows structural similarity to pilins, pseudopilins, and a killer toxin from *Millerozyma farinosa*. (**A**) Core K1 superfamily toxin motifs and (**B**) bacterial pilins, pseudopilins from *Pseudomonas aeruginosa* (PilE (PDB: 4noa), PilA (PDB: 3jyz), XcpV (PDB: 5bw0)), *Streptococcus sanguinis* (PilA (PDB: 7o5y)), *Shewanella oneidensis* (PilBac1 (PDB: 4d40), *Vibrio vulnificus* (EpsI (PDB: 2ret)), and a killer toxin from *Millerozyma farinosa* (SMKT (PDB: 1kvd)) identified by DALI analysis as homologs of K1 superfamily killer toxins. Secondary structure elements that are conserved across structures are colored to highlight similarities, including the central hydrophobic helix (orange) and a second conserved helix (magenta) that is positioned before and after β-strands (cyan), ending in C-terminal β-strands (dark blue).

Despite low sequence identity among K1, K2, K45, and K74 families of killer toxins (7.7–32.0%), they exhibit conserved secondary and tertiary structure. Alignment of the tertiary structure models of K1, K2, K45, and K74 families of killer toxins revealed a well-defined core motif of two α-helices and a single β-sheet composed of four to seven antiparallel β-strands (Figure 7A). The first α-helix in this motif is the central α-helix of the alpha domain that is buried in all tertiary structure models. The second α-helix from the beta domain interacts with this central α-helix, with the only exception being the model of K2. The majority of β-strands are formed from the primary sequence between the two α-helices, with the exception of two C-terminal β-strands. A conserved structural motif is observed at the C-terminus of the beta domain, consisting of a β-strand followed by an α-helix and then two additional β-strands. Importantly, the final β-strand is inserted between the two other β-strands to form an antiparallel β-sheet (Figure 7A). Overall, this conserved core motif has an average RMSD of 4.5 Å across the K1, K2, K45, and K74 families of killer toxins, compared to 8.7 Å when comparing the pTox structures (Table S10).

The central α-helix of the K1, K1L, K2, K21, KHS, and K45 killer toxins was always located within the alpha domain, which has been shown to be responsible for cytotoxicity [115,170]. PSIPRED analysis predicted that the central α-helix in all K1 superfamily toxins interacts with membranes. Specifically, four of these α-helices are predicted to be transmembrane helices (K2, K21, KHS, and K45), and three are amphipathic pore-lining α-helices (K1, K1L, and K74). The differences in PSIPRED predictions could indicate mechanistic differences in membrane attack, especially as K2, K21, KHS, and K45 have additional α-helices and β-sheets in the alpha domain that are also predicted to interact with membranes. In the pTox, the central α-helix is sequestered and buried, likely to prevent unwanted interactions with membranes and associated toxicities while enabling the correct folding and association of the alpha and beta domains (Table S6). The alpha domains all share a conserved disulfide bond that pins the N-terminal end of the central α-helix to the C-terminal end of the preceding secondary structure element in alpha (Table S5). This bond may play a conserved role in positioning or constraining the conformational flexibility of the central α-helix.

Overall, the structural conservation and biochemical characteristics of the central α-helices support the proposed antifungal mechanism of these toxins, namely, membrane disruption likely through pore formation. The close structural similarities of these toxins also support the proposal that their mechanism of intoxication is conserved and that they represent a broader “K1 superfamily”, a naming convention based on the early discovery of K1. As such, the extensive mechanistic insights gained from the study of K1 and K2 can be broadly applied to the wider superfamily, including hundreds of homologous sequences that remain uncharacterized.

To identify structural homologs of the killer toxins of the K1 superfamily, predicted pTox structures were used to query the protein database using the DALI server. The top hit for four of the seven K1 superfamily killer toxins (K1, K1L, KHS, K45) identified structural matches to bacterial pilins or pseudopilins (z-scores 3.3–4.5) (File S1). Analysis of the structural homologs shared among the different killer toxins identified ten proteins that were homologs of four or more killer toxins. Of these ten, six were pilins or pseudopilins from five different species of bacteria (Table S11). The pseudopilin EpsI (pdb; 2ret) from the type 2 secretion system of the bacteria *Vibrio vulnificus* was identified as having structural homology to six of the seven killer toxins, with an average z-score of 3.0 and RMSD of 6.4 Å (Figure 7B and Tables S10 and S11). The structural similarity with killer toxins is due to the characteristic pilin motif, which is an α-helix that is partially wrapped by a four-stranded antiparallel β-sheet (Figure 7 and Figure S7) [179]. The identification of these structural homologs was unexpected, as pilins and pseudopilins are not toxins, but subunits of helical filaments that extend from bacterial cells to aid in processes such as effector secretion, DNA uptake, adhesion, and motility.

Filament formation, similar to that of pilins, is a mechanism of membrane attack and has been observed for the insecticidal cytolysins (Cyt) from the biocontrol bacterium *Bacillus thuringiensis* [180,181]. Cyt toxins have similar tertiary structures to pilins and K1 superfamily toxins, with an alpha/beta/alpha sandwich organization [182,183]. Similar to Cyt toxins, K1 can oligomerize into large complexes or aggregates, but has been considered as evidence of membrane pore formation [112,154,155]. For Cyt toxins, there are two models of their cytotoxic mechanism: one predicting a detergent-like action of filaments and the other involving the formation of membrane pores [184,185,186,187]. Importantly, filament formation and pore formation may not be mutually exclusive mechanisms of Cyt toxin membrane attack and may depend on the biological properties of the target membrane and toxin concentration [184,188]. Whether the structural similarity of K1-superfamily proteins to pilins is biologically relevant or a coincidence of their common structural organization remains to be further investigated.

In addition to pilins, DALI also identified two notable homologs to the K1 superfamily: the antifungal protein ginkbilobin-2 (Gnk-2) from the *Ginkgo biloba* tree and the salt-mediated killer toxin (SMKT) from *Millerozyma farinosa* (File S1) [189,190]. Gnk-2 was more structurally similar to K1L (z-score 3.5), while SMKT was more similar to KHS, but with a low z-score of only 2.1. Notably, homology between the empirically determined structure of SMKT and a tertiary structure model of mature K2 has been reported, with shared secondary and tertiary structures and functional motifs (Figure 7B) [169]. The RMSD of SMKT to the pTox model of K2 was 6.2 Å, but it was a closer structural match to other K1 superfamily toxins, specifically K1 (5.2 Å), K45 (5.2 Å), and KHS (5.9 Å).

Both Gnk-2 and SMKT are alpha/beta sandwich proteins belonging to a diverse family of cytotoxic proteins and lectins (Pfam: PF01657, PF21414, PF21415). Like K1, the antifungal mechanism of SMKT is thought to involve membrane attack and can permeabilize artificial liposomes, consistent with pore formation [191]. Unlike K1, SMKT does not specifically bind the yeast cell wall but directly interacts with membranes of various compositions [191]. SMKT has a domain organization of alpha/gamma/beta, similar to that of the K1 superfamily, and an amphipathic α-helix in the alpha domain. The SMKT alpha domain stably associates with fungal membranes, whereas the beta domain associates only loosely. This function mirrors that of the proposed K1 mechanism, in which the hydrophobic alpha domain alone is toxic to yeasts and is predicted to associate with membranes. Similar to SMKT, the K2, K45, and K74 toxins are predicted to lack interdomain disulfide linkages, and association of the alpha and beta domains is stabilized by non-covalent interactions [192]. Despite variations in sequence and size, their conserved structural features and domain organization suggest a common mechanism of action across killer toxins, most likely centered on membrane disruption. However, additional structural analysis presented below suggests that SMKT may also be more closely related to the Klus family of killer toxins.

### 2.8. The Klus Family

#### 2.8.1. The Klus Family Introduction: Discovery and Early Characterization

Klus was discovered during a screen for *S. cerevisiae* killer yeasts associated with 110 spontaneous grape fermentations in the Ribera del Guadiana region of Spain [94]. The screen isolated 423 killer yeast strains, with the majority being of killer type K2. However, 7% of the killer yeasts exhibited a unique spectrum of antifungal activity, due to the production of the killer toxin Klus [94]. Klus killer yeast strains inhibited the growth of K1, K2, and K28 killer yeasts and were resistant to their own toxins. As with other *Saccharomyces* killer toxins, Klus is encoded by a dsRNA satellite named Mlus, which has been identified in *S. cerevisiae* from other regions of Spain and around the world [163]. Curing of Mlus from *S. cerevisiae* resulted in the loss of killer toxin production. Klus is most active at pH 4.0 to 4.7 and between 28 °C and 30 °C (Table 1). Klus was also previously shown to share sequence and structural homology to an extracellular protein of unknown function encoded by the gene *CSS2* (YFR020W) in *S. cerevisiae* [193].

KHR is a genomically encoded toxin in *S. cerevisiae* isolated from Japanese wineries in Yamanashi Prefecture and has been identified in many *S. cerevisiae* [20]. KHR is thermostable, retaining its antifungal activity from 0 °C to 40 °C and over a pH range of 5.0 to 6.0 (Table 1) [104]. Mature extracellular KHR has an observed molecular weight of ~20 kDa, indicating that it is likely post-translationally modified by proteolytic cleavage and is close to the theoretical molecular weight of an alpha and beta domain heterodimer (18.4 kDa). It has antifungal activity against *N. glabratus*, *S. cerevisiae*, and *Kluyveromyces lactis* but is relatively understudied with respect to its function as a toxin.

#### 2.8.2. The Klus Family: Domain Organization and Maturation

**Figure 8:**
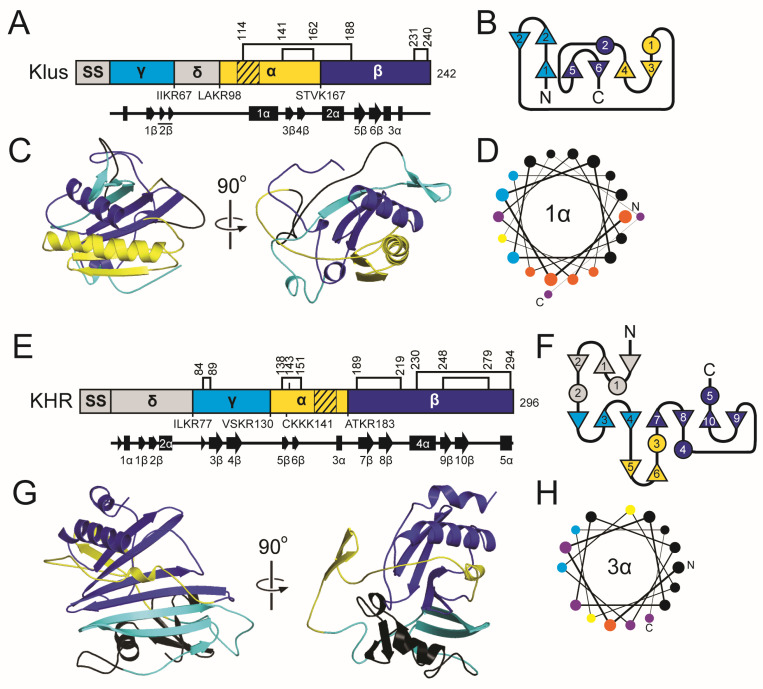
Secondary and tertiary structure models of Klus family killer toxins. (**A**,**E**) Domain diagrams of Klus and KHS ppTox indicating the sites of proteolytic processing that define the delta, alpha, gamma, and beta domains. The positioning of Kex protease cleavage sites is indicated below the domain diagram, with the four amino acids preceding the cleavage point illustrated. Cysteine residues are indicated by lines and numbers above the domain diagram, with connections between them representing predicted disulfide bonds. Hatching in the diagram represents amino acid sequences predicted to form transmembrane or pore-forming structures. Secondary structure, with numbered α-helices and β-strands, is represented below the domain diagram by arrows and rectangles, respectively. (**B**,**F**) Two-dimensional schematic of the relative secondary structure organization of Klus and KHR, with α-helices represented as circles and β-strands as triangles (numbered as in the linear representation of the secondary structure). N = amino-terminus, C = carboxyl-terminus. (**C**,**G**) Tertiary structure model of killer toxin pTox colored by domains as depicted in panel (**A**). (**D**,**H**). Helical wheel diagram of 2α helix, with hydrophobic (black), positively charged (yellow), negatively charged (blue), polar (purple), and aromatic (orange) amino acids. The line thickness between amino acids (from thick to thin) represents the progression of the sequence from the N-terminus to the C-terminus. During the MD simulation, the 3α helix unfolded; therefore, panel (**D**) represents the relaxed AlphaFold2 predicted α-helix before it was subjected to MD.

Klus has three predicted dibasic Kex2 cleavage sites, resulting in a classic four-domain configuration (amino acids 24–67, 68–98, 99–167, and 168–242), similar to other known killer toxins (Figure 8 and Table S4) [94]. This organization includes a predicted signal sequence cleavage site after amino acid 23, which would allow entry into the secretory pathway. However, the location of the six cysteine residues in the last two domains of the protein represented a unique configuration compared to other killer toxin families, which typically have most of their cysteines in the second (alpha) and last (beta) domains (Table S5). Klus is also predicted to have a transmembrane region that includes a portion of the 1α helix in the third domain from the N-terminus. Based on these data and additional modeling and comparison with empirically determined structural models, the domain organization for Klus is predicted to be gamma, delta, alpha, and beta.

Overall, KHR has a similar pattern of secondary structure compared to Klus, but is 54 amino acids longer with additional structural elements and one additional Kex2 cleavage site (Figure 8 and Table S4). The positioning of the proteolytic cleavage sites suggested that KHR has four domains between amino acids 22–77, 78–130, 131–183, 184–296, and a signal sequence cleavage site after residue 21. As is the case for Klus, a predicted transmembrane region is located in the third domain from the N-terminus of KHR. KHR also has 11 cysteine residues, which, like Klus, are mostly positioned in the third and fourth domains, supporting their assignment as the alpha and beta domains in the C-terminal half of the toxin (Table S5). KHR was identified as a sequence homolog of Klus, and the positions of the alpha and beta domains are the same, but the secondary structure organization suggests that the gamma and delta domains are reversed in KHR relative to Klus (delta, gamma, alpha, and beta). Further justification for the unique rearrangement of the structural domains of KHR and Klus is presented below.

#### 2.8.3. The Klus Family: Molecular Modeling Results

The tertiary structure of Klus is a globular protein composed of a central 1α helix (alpha domain) positioned next to a shorter 2α helix (beta domain), which is offset from parallel by ~45 degrees (Figure 8). The 1α helix is amphipathic and predicted by PSIPRED to be pore-lining, consistent with an ionophoric mechanism of action. Similar to other killer toxins, the 1α and 2α are cradled by a five-stranded discontinuous β-sheet, with 1α interacting with 3–4β and 2α with 1β/5–6β. The main β-sheet composed of β-strands from the gamma, alpha, and beta domains, with the 2α helix, creates a pocket that buries 1386.0 Å^2^ (59.3%) of 1α (Table S6). The β-sheet consists of two pairs of discontinuous β-strands (3–4β and 5–6β) that are aligned in an antiparallel configuration. β-strand 1β of the gamma domain is aligned in parallel to strand 5β of the beta domain on the outer edge of the β-sheet. The inclusion of 1β into the β-sheet that wraps the hydrophobic α-helix is similar to the gamma-domain interactions observed in other *Saccharomyces* killer toxins. This justifies assigning the Klus N-terminal domain as gamma and the second domain as delta, as the latter does not contribute to the beta sheet wrapping of the alpha domain helix. The opposite side of the β-sheet, relative to 1α and 2α, is a tangle of unstructured protein sequence, consisting of the N-terminus, C-terminus, and a 36 amino acid loop that straddles the delta and alpha domain boundary.

Predicted disulfide bonds in Klus connect the alpha and beta domains via the N-terminal end of 1α and the C-terminal end of 2α (C114–C188) and the C-terminal end of 1α to the N-terminal end of 2α (C141–C162). A third pair of disulfide bonds stabilizes an unstructured loop at the C-terminus of Klus; a similar linkage was observed for the majority of killer toxins in the K1 superfamily. Proteolytic processing of the pTox by Kex2 would mean that C114–C188 would tether the alpha and beta domains, whereas C141–C162 would pin the 3–4β sheet to 1α. This organization of disulfide bonds also supports the unique domain organization of Klus, and that the mature toxin is a heterodimer after cleavage and dissociation of the N-terminal delta and gamma domains.

KHR is also a globular protein with a similar overall organization to Klus, composed of two α-helices: 3α (alpha domain) and 4α (beta domain), which are positioned ~45 degrees apart. The 3α helix of the AlphaFold2 model is a 17-residue hydrophobic helix, but unraveled to a predominantly unstructured coil and smaller α-helix after MD simulations. The remaining α-helical structure buried a surface area of only 380.5 Å^2^ (Table S6). KHR has a predicted domain order of delta, gamma, alpha, and beta, with the gamma and beta domains contributing to the organization of a six-stranded discontinuous β-sheet formed from three pairs of antiparallel β-sheets (3–4β, gamma; 7–8β and 9–10β, beta). The inclusion of 3–4β into the β-sheet justifies the assignment of the gamma domain as the second domain from the N-terminus. As with other killer toxins of similar structure, the delta domain does not contribute any beta strands to the beta sheet. Unlike Klus, the extensive β-sheet does not include strands from the alpha domain. The opposite side of the β-sheet to the wrapped helices is a region of extensive elaboration compared to Klus, featuring small α-helices and β-sheets, as well as large unstructured loops from the delta, gamma, and alpha domains. Curiously, the alpha domain contains a dibasic cleavage site that splits the domain but is bridged by a predicted disulfide bond between C138 and C151 (Table S5). In KHR models, there is no indication of interdomain disulfide bonds, as was predicted for Klus and other killer toxins.

**Figure 9:**
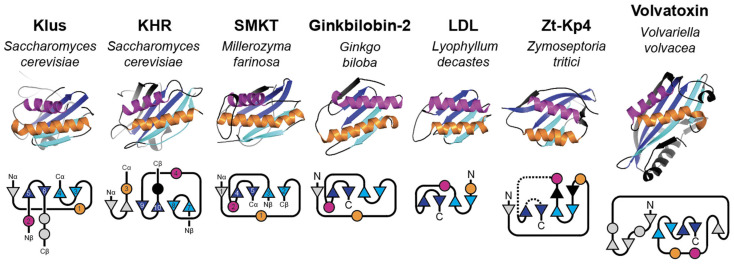
Mature structures of Klus and KHR compared to tertiary structure homologs. Secondary structure is colored to emphasize β-strand pairs and α-helices common to the tertiary structure of all proteins. Below the tertiary structures are two-dimensional schematics of the relative secondary structure organization of Klus and KHR and their structural homologs, with an emphasis on the relative organization of the β-sheet. Helices are represented as circles and β-strands as triangles; N = amino-terminus, C = carboxyl-terminus, Nα/Cα = alpha domain amino/carboxyl-termini, Nβ/Cβ = beta domain amino/carboxyl-termini. Secondary structure elements of mature Klus, KHR, and SMKT are labeled based on pTox (Figure 8 and Figure 10).

To better compare Klus and KHR with existing empirical protein structures, models of the mature toxin structures comprising only alpha and beta domains were constructed using AlphaFold2’s multimer prediction mode. These heterodimeric structures had high confidence (average pLDDT 71.0 (KHR) and 72.4 (Klus)) and were stable over a 1 µs MD simulation (Figure S8 and Table S12). The high confidence of the predicted mature toxins contrasted with the low confidence of other alpha/beta heterodimers of *Saccharomyces* killer toxins, which had an average pLDDT score of 45.4 (Table S12). During MD, the Klus and KHR structures reached similar conformations within 10 ns, with the structures stabilizing around 0.3 nm (Figure S8). The mature Klus and KHR did not recapitulate the exact structural organization of their respective pTox as they folded new regions of secondary structure and rearranged existing structural elements. This included the extension of the previously small 3α helical region in KHR to a long 54 amino acid α-helix that was similar to 3α predicted by AlphaFold before MD (compare Figure 8G to Figure 9). In Klus, 1β of the gamma domain was replaced with β-strands folded from an unstructured loop of the alpha domain. For KHR, β-strands 3–4β of the gamma domain were replaced by 5–6β from the beta domain. Disulfide bonds in mature Klus were unchanged from pTox and indicated that the mature heterodimer is bonded between the alpha and beta domains by C114–C188. For mature KHR, the disulfide configuration was significantly different from pTox, with only C248–279 maintained between the predicted pTox and mature toxins. Alternative disulfide pairings were predicted that created two interdomain bonds between alpha and beta domains (C138–C294 and C143–C230) and one intradomain bond in the beta domain (C189–C219).

**Figure 10:**
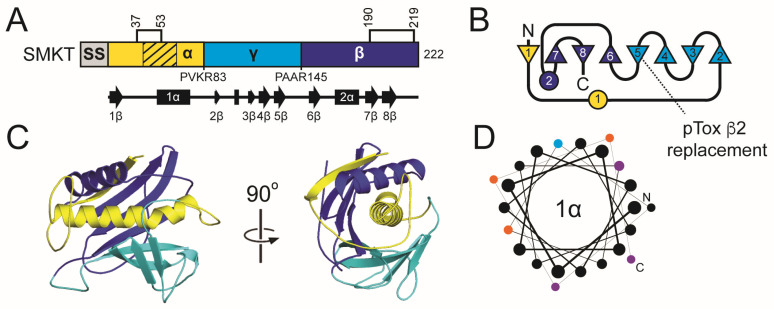
Secondary and tertiary structure models of the SMKT pTox. (**A**) Domain diagrams of SMKT ppTox indicating the sites of proteolytic processing that define the alpha, gamma, and beta domains. The positioning of Kex protease cleavage sites is indicated below the domain diagram, with the four amino acids preceding the cleavage point illustrated. Cysteine residues are indicated by lines and numbers above the domain diagram, with connections between them representing predicted disulfide bonds. Hatching in the diagram represents amino acid sequences predicted to form transmembrane or pore-forming structures. Secondary structure, with numbered α-helices and β-strands, is represented below the domain diagram by arrows and rectangles, respectively. (**B**) Two-dimensional schematic of the relative secondary structure organization of Klus and KHR, with α-helices represented as circles and β-strands as triangles (numbered as in the linear representation of the secondary structure). N = amino-terminus, C = carboxyl-terminus. (**C**) Tertiary structure model of SMKT pTox colored by domains as depicted in panel (**B**). (**D**) Helical wheel diagram of 2α helix, with hydrophobic (black), negatively charged (blue), polar (purple), and aromatic (orange) amino acids. The line thickness between amino acids (from thick to thin) represents the progression of the sequence from the N-terminus to the C-terminus.

#### 2.8.4. Mechanistic Insights into the Klus Family

Comparison of the tertiary structures of Klus and KHR revealed average RMSD values of 5.7 Å (pTox) and 4.1 Å (mature alpha/beta heterodimer), confirming the observed sequence similarities. Querying the DALI server with these models identified homology to alpha/beta sandwich protein families (Pfam: PF01657, PF21414, PF24145, and PF09044) (File S1). In particular, the PF01657 family includes a large (30,000+) subfamily of plant-specific proteins that contain the Domain of Unknown Function 26 (DUF26), including cysteine-rich receptor kinases and secreted cysteine-rich proteins. These proteins are used by plants for responding to environmental and biotic stressors, including bacteria and fungi [194]. Many of the structural homologs had a pair of α-helices on one side of a four- to six-stranded antiparallel β-sheet (Figure 9). Consistent with the cytotoxic activities of Klus and KHR, many structural homologs were identified that are cytotoxic to fungi, including the antifungal proteins Gnk-2, SMKT, and Zt-KP4 [110,189,190]; other homologs are toxic to cells of higher organisms (Lyophyllum decastes lectin (LDL), and Volvatoxin A2 (VVA2)) [195,196] (Figure 9). Compared to prior pTox models of K2, K1L, and KHS that were found to have structural similarity to SMKT (RMSD = 5.2–6.2 Å), the alignment of mature Klus and KHR to the empirical SMKT structure resulted in lower RMSD values, indicating a better structural match (3.2 Å and 4.1 Å, respectively). Klus also showed structural homology with a newly described killer toxin produced by the yeast *Candida sinolaborantium*, isolated from the gardens of attine ants [12].

Despite the close tertiary structure match to Klus and KHR, the domain order of SMKT is more similar to other canonical killer toxins (delta/alpha/gamma/beta) (Figure 10) [197]. However, it was unclear whether the pTox structure of SMKT would better match the K1 superfamily or the Klus family of toxins. To compare the structures of these immature toxins, the pTox structure of SMKT was determined by AlphaFold (Figure 10). The predicted configuration of the SMKT pTox overlapped almost perfectly with the alpha/beta heterodimer of the mature SMKT (RMSD 0.28 Å), but with the replacement of pTox β5 from the gamma domain with a β-strand folded from an unstructured loop in the alpha domain of the mature SMKT [169]. The close similarity in the tertiary structures of SMKT and its pTox meant that Klus and Klus also had a similar tertiary structure to SMKT pTox (RMSD, 3.6 Å and 4.2 Å) (Table S13). SMKT pTox also showed structural homology to the K1-superfamily, but with a higher RMSD than Klus and KHR (5.2–5.3 Å). As was observed in the pTox models of the K1 superfamily, Klus, and KHR, the SMKT pTox wrapped a central α-helix (1α) with a large eight-stranded antiparallel β-sheet assembled from β-strands from the alpha (1β), beta (6–8β), and gamma (2–5β) domains (Figure 10B,C). The similar structures underscore the importance of the respective gamma domains in pTox folding and in the burial of the centrally positioned transmembrane α-helix in SMKT and *Saccharomyces* killer toxins (Figure 10). Killer toxin immunity is another known function of the killer toxin gamma domain of K1, but differences in the Klus and KHR domain order and the positioning of gamma domain β-strands could indicate a non-canonical immunity mechanism. Moreover, it is unclear whether SMKT encodes functional immunity, as ectopic expression by *S. cerevisiae* results in a suicidal phenotype [198]. Overall, the tertiary structures of Klus and KHR are most similar to SMKT, despite their unusual domain organization.

The intoxication mechanism of SMKT is thought to involve disruption of biological membranes, providing possible insights into the antifungal mechanisms of Klus and KHR [191]. Intoxication by SMKT appears to be independent of cell wall binding, but dependent on membrane interaction [191,199]. SMKT also shows structural homology with VVA2 from the mushroom *Volvariella volvacea* and with cytolytic (Cyt) toxins from the bacterium *Bacillus thuringiensis*, both of which can permeabilize and lyse cells [200,201]. Cyt toxins have been shown to cause specific ion leakage, leading to a model of pore formation at high toxin concentrations, with the possibility of a detergent-like mechanism at low concentrations [184,185,186,187]. Moreover, VVA2 and Cyt toxins can oligomerize into filaments (as discussed in the K1 superfamily section) [182,183]. Cyt toxin filaments are thought to cause membrane delamination and the collapse of large liposomes to smaller vesicles and lipid aggregates. A similar dramatic destruction of liposomes has also been observed by SMKT, but it is unclear whether this mode of action requires oligomerization [191].

Amphipathic α-helices in the alpha domains of SMKT and Klus family proteins are positioned in the same location as amphipathic α-helices in Cyt and VVA2 (Figure 9). In VVA2, this α-helix is important for oligomerization and toxicity, but not for membrane interaction [202]. Indeed, C-terminal β-strands of VVA2 and Cyt toxins associate with membranes, suggesting the formation of β-barrel pores [202,203,204]. The membrane association of the C-terminal domains of Cyt and VVA2 contrasts with SMKT, which stably associates its N-terminal alpha domain with the membrane fraction of intoxicated yeasts [191]. A model of alpha domain membrane attack would be more similar to the K1 superfamily of toxins. Additionally, the C-terminal beta domain of SMKT appears to be loosely associated with membranes, which would argue against a model of C-terminal domain β-barrel formation demonstrated by VVA2 and Cyt toxins [191].

Other mechanistic insights into the antifungal activities of Klus and KHR can also be drawn from the KP4-like killer toxin from the wheat pathogen *Zymoseptoria tritici* (Zt-KP4-1) [110]. This toxin is a close structural homolog of the well-studied antifungal protein KP4 from the corn-smut fungus *Mycosarcoma maydis* (syn. *Ustilago maydis*) and toxin homologs from other fungal pathogens [82,102]. However, unlike the predicted pore-forming mechanisms of SMKT, KP4 blocks L-type voltage-gated calcium channels in both fungal and mammalian cells [80,205]. KP4 and its homologs also inhibit plant root growth [82,83]. Consistent with the interference of ion homeostasis, high concentrations of calcium can protect cells and plants from intoxication by KP4 and its homologs [82,83,102]. However, the precise molecular interactions by which KP4 targets and disrupts calcium homeostasis remain unresolved, as does whether KP4 and its homologs interact directly or indirectly with calcium channels.

Other alpha/beta sandwich proteins with structural similarity to Klus and KHR include the α-galactosyl binding lectin from the mushroom *Lyophyllum decastes* (LDL), the glycan binding protein Y3 from *Coprinus comatus*, and Gnk-2. These proteins are toxins with activities against higher eukaryotic cells (LDL and Y3) and fungi (Gnk-2) [206,207,208]. How these toxins damage cells is unknown, but all bind specific carbohydrates and have defined surface-binding pockets on the same face of the tertiary structure close to the C-terminal end of the second α-helix [207,208,209]. Carbohydrate binding suggests the possibility of similar motifs in Klus and KHR, given their conserved tertiary structures. The potential for carbohydrate binding by Klus and KHR would be similar to other killer toxins that have carbohydrate cell wall receptors, i.e., glucans and mannan. However, SMKT is also a structural homolog of Klus and KHR and appears not to require cell wall binding to cause cytotoxicity [199].

The structural similarity of Klus and KHR to other alpha/beta sandwich proteins offers insight into their potential mechanisms of action. Members of this protein family are known to kill cells by disrupting membrane permeability or ion channel function, indicating that the alpha/beta sandwich fold can support diverse cytotoxic functions. However, the exact mechanisms underlying these activities remain unresolved, and it is unclear whether observed functional differences reflect true mechanistic divergence or variation in experimental systems. A key goal for future research is to determine whether these proteins share a common mode of intoxication or if substantial functional diversification has occurred around a conserved tertiary structure.

### 2.9. The K28 Family

#### 2.9.1. The K28 Family Introduction: Discovery and Early Characterization

The first K28 killer yeast was isolated from grapes and identified by screening 163 yeasts collected by the Johannes Gutenberg-Universität Mainz for the killer phenotype [113]. Initially described as an *S. cerevisiae* toxin, more recent work has identified K28 as being produced by *S. paradoxus* [97]. The antifungal activities of K28 appear to be different from those of other killer toxins, and K28 killer yeasts are uniquely sensitive to K1 and K2, suggesting a distinct toxin type [114]. K28 is encoded on a satellite dsRNA named M28, and curing of the dsRNA with cycloheximide caused the concomitant loss of the K28 killer phenotype [210]. Determination of the M28 genetic sequence identified the K28 gene but revealed no sequence homology to any other protein in the database, and this still remains true (Figure 1) [211]. The antifungal activity of K28 is optimal at pH 5.8, which is less acidic than optimal conditions for other killer toxins [113,114]. K28 is also more tolerant of higher temperatures, remaining active at 40 °C for up to one hour (Table 1).

#### 2.9.2. The K28 Family Introduction: Domain Organization and Maturation

**Figure 11:**
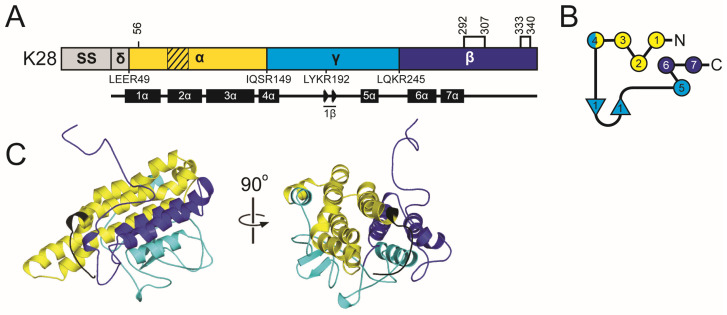
Secondary and tertiary structure models of the K28 killer toxin. (**A**) Domain diagram of K28 ppTox indicating the sites of proteolytic processing that define the delta, alpha, gamma, and beta domains. The positioning of Kex protease cleavage sites is indicated below the domain diagram, with the four amino acids preceding the cleavage point illustrated. Cysteine residues are indicated by lines and numbers above the domain diagram, with connections between them representing disulfide bonds. Hatching in the diagram represents an amino acid sequence predicted to be transmembrane or pore-forming lining. Secondary structures, including numbered α-helices and β-strands, are represented below the domain diagram by arrows and rectangles, respectively. (**B**) Two-dimensional schematic of the relative secondary structure organization of K28 with α-helices represented as circles and β-strands as triangles (numbered as in the linear representation of the secondary structure). Number of the secondary structure elements is consistent with panel (**A**). (**C**) Tertiary structure model of killer toxin pTox colored by domains as depicted in panel (**A**).

Like all other *Saccharomyces* killer toxins, K28 is trafficked through the secretory system to enable maturation and extracellular export. After translation, exportation of the ppTox to the ER is directed by an N-terminal signal sequence that is cleaved after G36. Mutagenesis of G36 and N-terminal sequencing of the 42 kDa K28 pTox confirmed the location of signal sequence cleavage [211,212]. Purification of the extracellular K28 resulted in the detection of a ~16 kDa disulfide-linked heterodimer consisting of two polypeptides that were named alpha (10.5 kDa) and beta (11.0 kDa) (Table S4). N-terminal sequence analysis of extracellular K28 confirmed that the alpha and beta domains started at amino acids 50 and 246, respectively [211]. The identification of alpha and beta led to the definition of the four functional domains of K28 pTox (delta, 36–49; alpha, 50–149; gamma, 150–245; beta, 246–344), based on the presence of dibasic motifs that are targeted by the Golgi-resident endopeptidases Kex1 and Kex2 (Figure 11) [212].

Consistent with the major role of Kex2 in the proteolytic processing of K28, a *kex2∆* null strain does not produce active K28 and is unable to cleave pTox to release the gamma and delta domains from pTox. Mutation of dibasic sites at the predicted domain boundaries prevented the expression of active K28 and resulted in incomplete pTox cleavage. Although not initially thought to be essential for K28 activity, Kex1 removes a critical C-terminal arginine residue to reveal an ER retention motif “HDEL” that is essential for efficient K28 retrograde trafficking into K28 susceptible cells [213,214].

With only a single cysteine residue present in the alpha domain (C56), it has been deemed essential for heterodimer formation by mutagenesis. Of the four additional cysteines present in beta, only C340 is essential for K28 toxicity, as mutagenesis of the remaining cysteines to tryptophan (C292W, C307W, C333W) has no apparent effect on K28 activity [212]. However, a more extensive follow-up study identified that C56–C333 was the more likely interdomain disulfide bond, with the remaining beta domain cysteine residues important for the toxicity and stability of K28 [215].

#### 2.9.3. The K28 Family Introduction: Antifungal Mechanism

The antifungal mechanism of K28 halts the cell cycle at G1/S and arrests DNA synthesis through an unknown mechanism [152,216,217]. Cell targeting relies on the interaction of the beta domain with a primary cell wall receptor and secondary cell membrane receptor [118,213,214]. K28 interacts with yeast cell wall mannans, specifically the short, branching mannose chains attached to the 1,6-linked mannose main chain, which are critical for K28 interaction with the cell wall [218,219]. Moreover, mutants lacking functional mannosyl transferases (i.e., *MNN1*, *MNN2*, and *MNN5*), which attach mannose residues to the main chain via 1,2 and 1,3 glycosidic bonds, are resistant to K28, but not to killer toxins K1 and K2, which use cell wall 1,6-β-D-glucan as their cell wall receptor [39,220,221].

Following the binding of the cell wall mannans, K28 is translocated to the cell membrane, where the beta domain interacts with a secondary cell surface receptor, Erd2, via a C-terminal HDEL motif [213]. This interaction allows the retrograde transport of K28 to the ER. K28 requires the protein disulfide isomerase (Pdi1) to prevent toxin inactivation by unproductive oligomerization in the more neutral pH of the ER [215]. K28 then crosses into the cytosol from the ER, likely using the Sec61 translocase complex, via a mechanism distinct from the ER-associated protein degradation (ERAD) pathway [222]. In the cytoplasm, the disulfide bond linking the alpha-beta heterodimer is reduced, and the beta domain is ubiquitinated and degraded. The alpha domain translocates to the nucleus, where it induces cytotoxicity by arresting cells at G1/S phase through an unknown mechanism [152,216,217].

#### 2.9.4. The K28 Family Introduction: Immunity

The immunity mechanism of K28 depends on the ppTox, which is analogous to other killer toxins but has a unique mechanism. Similar to K1, the K28 alpha domain is required for immunity and requires a C-terminal extension to fully protect from exogenous K28 [223]. For example, expression of the alpha domain confers partial immunity to exogenous killer toxin, whereas expression of alpha and gamma confers full immunity; the exact sequence of gamma is not essential for immunity [223]. K28 immunity is based on a fraction of ppTox being present in the cytosol to intercept mature K28 during its transit from the ER to the nucleus (via the cytosol). Complex formation between mature and ppTox K28 in the cytosol is thought to result in ubiquitination and the degradation of K28 by the proteasome [223].

In addition to ppTox-derived immunity, there is a dedicated Killer Toxin Defense factor, *KTD1*, that protects cells from mature extracellular K28 [45]. Alleles of *KTD1* in *S. cerevisiae* confer different levels of K28 resistance, and there are elevated rates of non-synonymous substitutions in the gene, suggesting that it is under positive selection and is possibly locked in a genetic arms race with K28. The mechanism of *KTD1* protection differs from ppTox immunity, as it appears to intercept K28 in the endosomal trafficking system, preventing retrograde translocation to the nucleus. Indeed, Ktd1 requires the conserved oligomeric Golgi (COG) complex for proper antitoxin function and correct localization [44]. Signatures of positive selection in *KTD1* suggest a direct interaction with K28, and this may reroute the toxin to the vacuole, preventing it from reaching the nucleus and arresting cells in G1/S phase.

#### 2.9.5. The K28 Family: Molecular Modeling Results

The molecular model of K28 had a low pLDDT confidence with an average of 35.0, a minimum of 19.0, and a maximum of 60.4 (Figure 11 and Figure S3). Modeling with AlphaFold3 did not improve the confidence in the predicted structure (pTM = 0.21) [178]. The two predicted structures shared a similar overall organization but differed in the alignment of the polypeptide backbone (RMSD: 9.7 Å). MD simulation of the AlphaFold2 model also indicated structural instability, with flexibility in the gamma domain, the N-terminal and C-terminal sequences, and helix 3α (Figure S4). The lack of sequence homologs of K28 prevented a broader modeling study, as was done for the K74 family. The model’s low confidence means the following insights into the structure and function of K28 should be treated with caution.

The K28 model was predominantly α-helical (51.1%) and assembled into an α-helical bundle composed of seven α-helices aligned along a single axis. Flexible loops of varying length enabled the antiparallel packing of the α-helices. The alpha domain of K28 contains α-helices 1–4α, the gamma domain includes part of 4α and 5α, and the beta domain α-helices 6–7α. Helices 2α and 6α were in the core of the structure, surrounded by other α-helices, with 2α of the alpha domain predicted to be transmembrane. Domain boundaries defined by Kex cleavage sites were located on exposed, flexible loops that would be readily accessible for proteolysis during K28 maturation. The C-terminal tail of K28 contained the HDELR motif that is essential for retrotranslocation from the cell surface to the cytoplasm. In the tertiary structure model, this motif was exposed at the end of a flexible linker, making it accessible to the action of the Kex1 carboxypeptidase, which removes the terminal arginine, allowing for recognition by the Erd2 receptor.

The K28 model did not predict a disulfide bond between the alpha and beta domains as reported in the mature toxin (C56–C333). Instead, the four cysteines in the beta domain formed a pair of intradomain disulfide bonds with C56 remaining unbonded (Table S5). MD simulations revealed that the K28 model was flexible and that C56 moved from 51.7 Å to 20.1 Å relative to C333. The observed structural flexibility could allow the linkage of the alpha and beta domains via a C56–C333 disulfide bond, as well as alternative disulfide configurations that may account for unproductive oligomerization at neutral pH [215]. Disulfide rearrangement also enables the release of the alpha domain into the cytoplasm, a critical step for intoxication by mature K28. As cytoplasmic K28 ppTox is essential for immunity to exogenous mature toxin, the tertiary structure model could represent the reduced immature toxin. Despite the low confidence of the current model of pTox K28, it does provide insights into the structural flexibility of immature K28 and the possibility of alternative conformations relevant to K28 immunity, maturation, export, retrograde transport, and intoxication.

#### 2.9.6. Mechanistic Insights into the K28 Killer Toxin Family

Analysis of the K28 tertiary structure model using DALI identified 34 protein structures with z-scores greater than 4.0, which are functionally diverse and include ferritins, saccharide translocases, interleukins, and motor proteins (File S1). The top hit from DALI analysis, with a z-score of 5.8, was the C-terminus of *S. cerevisiae* Swt1 (PDB: 4pqz), an RNA endonuclease that is a member of the Higher Eukaryotes and Prokaryotes Nucleotide binding protein (HEPN) superfamily [224,225]. The Swt1 HEPN domain matches four α-helices in pTox K28. However, posttranslational cleavage of pTox would separate these α-helices, disrupting the predicted HEPN domain structure. Furthermore, the K28 alpha domain alone is responsible for toxicity and lacks the conserved consensus motifs required for hydrolysis of the phosphodiester backbone of nucleotides. Thus, it is unlikely that the structural match between K28 and the HEPN domains is functionally relevant. Given the functional diversity of the remaining proteins identified by DALI and the low confidence of the K28 model, we concede that the structure and antifungal mechanism of K28 continue to remain enigmatic.

### 2.10. The K62 Killer Toxin Family

#### 2.10.1. The K62 Family Introduction: Discovery and Early Characterization

The K62 killer toxin was first identified in the *S. paradoxus* strain Q62.5 isolated from an English oak tree. Based on its antifungal spectrum of activity against other killer yeasts, K62 was initially misclassified as a K1 toxin [10,176]. Challenging a greater diversity of yeast strains with the K62 killer yeast confirmed a different spectrum of antifungal activity compared to K1 or K28 toxins [49]. Further investigations of K62 found that it was encoded by the satellite dsRNA named M62, and the genetic sequence of the novel K62 killer toxin was confirmed [97]. Loss of M62 by exposure to cycloheximide led to loss of the K62 killer phenotype, confirming that the toxin gene was present on the dsRNA satellite [49]. However, loss of K62 expression did not result in loss of immunity, suggesting that immunity is encoded by the genome. Like other canonical *Saccharomyces* killer toxins, K62 has optimal activity at <30 °C and pH 4.0–4.5 (Table 1) [49].

#### 2.10.2. The K62 Family: Domain Organization and Maturation

**Figure 12:**
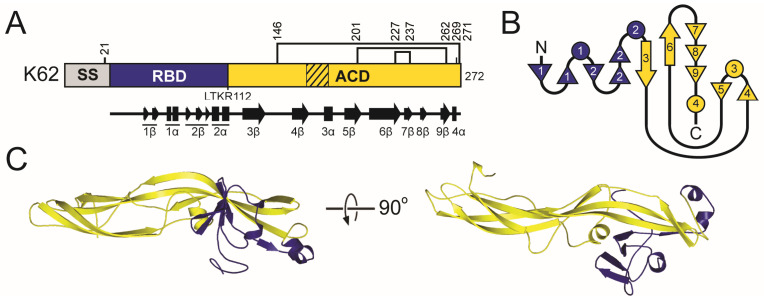
Secondary and tertiary structure models of K62 family killer toxins. (**A**) Domain diagram of ppTox K62 representing the N-terminal signal sequence (SS), putative receptor binding domain (RBD), and aerolysin core domain (ACD). The domain boundaries are defined by a predicted signal sequence cleavage site and a single site of proteolytic processing based on the position of a dibasic (KR) motif. Disulfide bonds are represented as lines between cysteine residues marked above the domain diagram. The hatched region indicates a predicted transmembrane sequence. Below the domain diagram is a representation of pTox K62 secondary structure with α-helices and β-sheets shown as rectangles and arrows, respectively. (**B**) Two-dimensional schematic of the relative secondary structure organization of K28, with α-helices represented as circles and β-strands as triangles (numbered as in the linear representation of the secondary structure). Number of the secondary structure elements is consistent with panel (**A**). (**C**) Tertiary structure model of K62 pTox colored by domains as depicted in panel (**A**).

K62 is a 272-amino acid protein, shorter than most known *Saccharomyces* killer toxins, which average 320 amino acids in length. K62 showed no detectable amino acid sequence homology to any previously characterized killer toxins, and secondary structure prediction indicated a high proportion of β-strands (41.6%) and few α-helices (4.1%). This secondary structure composition contrasts with other *Saccharomyces* killer toxins, which, on average, contain a higher proportion of α-helices (Figure 12).

K62 has a signal peptidase cleavage site between amino acids 31 and 32, which is consistent with it being an extracellular killer toxin. However, unlike all other *Saccharomyces* killer toxins, the K62 sequence contains only a single dibasic KR motif at residues 111 and 112. This motif is located on a solvent-exposed loop that separates the N-terminal domain from the elongated C-terminal domain. This location suggests a potential Kex2 protease cleavage site, analogous to those found in other *Saccharomyces* killer toxins (Table S4). However, unlike K1 and K28 killer toxins, such cleavage would separate the N- and C-terminal domains because the N-terminal domain lacks cysteine residues required to form an interdomain disulfide bond. PSIPRED predicted a region between 4β and 3α capable of interacting with membranes and forming pores, distinguishing K62 from other killer toxins, which predominantly harbor α-helices predicted to be transmembrane and pore-forming (Figure 12).

#### 2.10.3. The K62 Family: Molecular Modeling Results

The AlphaFold2 model of K62 predicted an elongated architecture (~86 × 40 Å), characterized by extended β-sheets, which deviates substantially from the compact globular folds typical of other *Saccharomyces* killer toxins. The C-terminal domain consists of a five-stranded β-sheet arrangement. In this configuration, β-strands 3β, 6–9β form a scaffold that is stabilized by three intramolecular disulfide bonds: one between C146–C271 linking the flexible loop between β-strands 3–4β to the C-terminal tail; a second between C201–C262 stabilizing β-strands 5β and 9β; and a third across a loop at the end of β-strand 6β (C227–C237) (Figure S5 and Table S5). These cysteines have been judged essential for toxin activity, as demonstrated by alanine mutagenesis [49]. A second sheet is formed by strands 4β and 5β flanking a flexible loop containing α-helix 3α. This loop has an alternating sequence of hydrophobic and polar residues, enriched in serine and threonine, residues often implicated in promoting oligomer assembly, facilitating membrane engagement, and pore formation in toxins.

#### 2.10.4. Mechanistic Insights into the K62 Family

**Figure 13:**
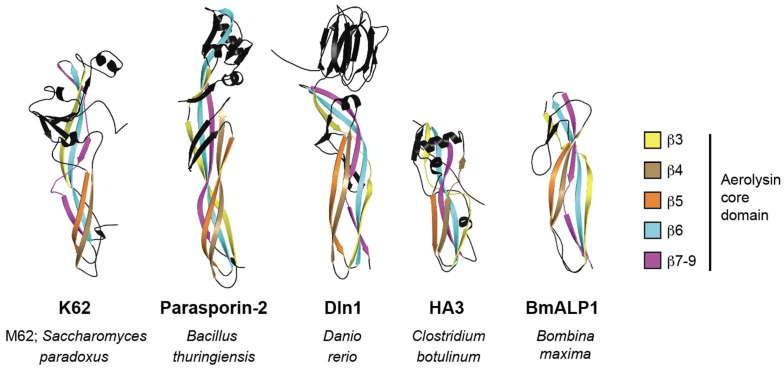
K62 structural homologs identified by DALI analysis. Tertiary structure models are colored by β-strands that contribute to the conserved five β-sheet motif of the aerolysin core domain in K62. The N-terminal receptor binding domain and insertion loop are colored black. PDB accession numbers for parasporin-2, Dln1, HA3, and BmALP1 are 2ZTB, 4ZNO, 2ZS6, and 6LH8, respectively.

Analysis of the predicted tertiary structure of K62 using DALI yielded a top hit of parasporin-2 from the bacterium *Bacillus thuringiensis* with a z-score of 5.2 (PDB: 2ZTB). Parasporin-2 is an aerolysin family protein, a class of β-barrel, pore-forming toxins that are bacterial virulence factors and highly toxic to eukaryotic cells [226,227,228]. Aerolysin toxins are thought to contribute to hemorrhaging and tissue necrosis in fish and small ruminants, with lethal dose 50% (LD-50) values as low as 100 ng/kg [227,229,230]. Moreover, parasporin-2 can recognize, permeabilize, and kill human cells [231]. The calculated RMSD of the aerolysin core domain of parasporin-2 compared to K62 was 6.9 Å, indicating structural homology despite only 16.8% and 32.1% amino acid identity and similarity. Several other K62 structural homologs from the aerolysin family of pore-forming proteins and toxins were also identified by DALI analysis (File S1). These homologs included members of a class of aerolysins found in bony fish and lamprey named natterins, which are theorized to play a role in innate immunity [232]. The natterin-like protein Dln1 from *Danio rerio* (zebrafish, z-score 4.3) binds mannans, including those of the fungal cell wall, a property shared with the yeast killer toxin K28. Dln1 also undergoes pH-dependent oligomerization, with an optimal pH similar to that of killer toxins [232]. Other K62 homologs were the HA3 toxin from the bacterium *Clostridium botulinum* (z-score 4.2) [233] and the innate immune stimulator BmALP1 from *Bombina maxima* (Yunnan firebelly toad, z-score 3.8) [234] (Figure 13). Notably, the receptor-binding domain of parasporin-2 and other K62 homologs had no structural similarity to the modelled K62 N-terminal domain. Bioinformatics studies have previously identified 41 fungal sequences from 19 different species that are homologous to aerolysin family proteins, but none are sequence or structural homologs of K62 by PSI-BLAST or DALI analysis [235]. Although K62 is the first aerolysin family protein found in the *Saccharomyces* genus of yeasts, it is not the first fungal aerolysin toxin. The monomeric structure and biological activity of a hemolytic lectin from *Laetiporus sulphureus* was previously identified as a novel fungal aerolysin family protein [236]. However, the structure of the aerolysin domain of this hemolytic lectin is not a close homolog of K62 (RMSD 9.8 Å).

Oligomerization of aerolysin monomers to form a pre-pore structure is an essential first step before membrane attack by pore formation and has been observed after K62 expression in both bacteria and yeast [49]. Based on known aerolysin family pre-pore structures, K62 β-strands 4β and 5β would oligomerize with the same β-strands of neighboring monomers in an alternating pattern to form an inner ring, while β-strands 3β, 6–9β form an outer ring [237]. After pre-pore formation, β-strands 4β and 5β and the insertion loop containing 3α would penetrate the membrane, forming the extended β-barrel pore [232,237,238,239,240]. The enrichment and alternating pattern of polar serine (S) and threonine (T) residues with hydrophobic amino acids in K62 (160_L**S**W**S**Y**T**Y**T**W**S**YDV**S**IGI**S**WEVI**S**A**S**VDY**S**I**S**Q**S**L**S**Y**S**_196) is characteristic of the membrane insertion loop of aerolysin-family toxins. The putative insertion loop of K62 also correlates with a predicted membrane-interacting region that was identified by PSIPRED (Figure 12). More detailed in silico modeling studies have shown that K62 can assemble oligomers resembling pore and pre-pore structures that are strikingly similar to empirically determined structures of other members of the aerolysin family [49]. Based on these similarities in sequence and structure, it is predicted that K62 is an ionophoric toxin that, upon oligomerization, forms a β-barrel pore to penetrate target membranes, leading to cell death.

Despite similarities to aerolysin toxins, K62 has several unique structural features. Specifically, the possible cleavage of the N-terminal domain from the aerolysin core domain by Kex proteases at a dibasic motif. This would represent a departure from the canonical aerolysin family of toxins, in which the N-terminal domain typically mediates membrane and receptor recognition. Removal of the K62 N-terminal domain would result in a structure similar to aerolysins that have a minimal N-terminal domain, such as monalysin, which has an aerolysin core domain that is necessary and sufficient for membrane targeting and pore formation [241]. It is therefore plausible that the K62 core domain alone retains toxicity and is independent of the N-terminal region. The N-terminal domain may remain associated with the aerolysin core domain via noncovalent interactions, as in the SMKT killer toxin [192]. Another possibility is that immature K62 pTox may prevent the unwanted toxicity of the mature K62 before it is exported from the cell. The removal of the N-terminal domain by Kex cleavage could activate the toxin, analogous to the proteolytic processing required for aerolysin activation [242,243]. Another unique feature of K62 is the presence of disulfide bonds, which are common in killer toxins and, in K62, are essential for toxicity.

Despite differences from known aerolysin toxins, confident structural predictions of K62 and its homology to parasporin-2 indicate it is a new member of the aerolysin family of toxins. The potency of aerolysin toxins and the presence of K62 and its homologs in fungal species relevant to plant, animal, and human health, as well as industrial fermentations, justify further study of their antifungal activities and toxicity to cells of higher eukaryotes.

## 3. Conclusions

This study presents a comprehensive structural and functional analysis of *Saccharomyces* killer toxins, integrating decades of empirical research with state-of-the-art protein structure prediction and MD simulation. These models will benefit the future study of *Saccharomyces* killer toxins, advancing the functional understanding of these antifungal proteins and increasing our knowledge of thousands of sequence and structural homologs. Using *S. cerevisiae* to study these toxins will also help clarify how they interact with and intoxicate fungal cells, as well as their potential future applications against pathogenic and spoilage fungi. Furthermore, the homology of killer toxins to bacterial virulence factors and other cytotoxic proteins warrants investigation into their potential contribution to diseases in humans, plants, and animals.

**Figure 14:**
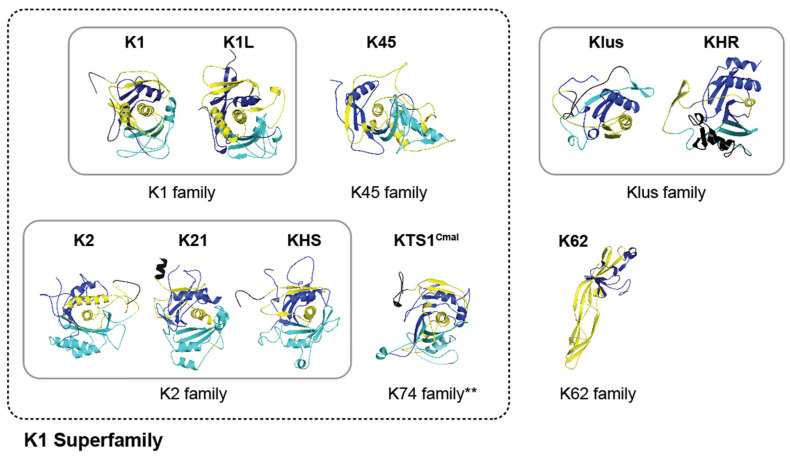
Summary of the proposed family and superfamily organization of *Saccharomyces* killer toxins. Confident tertiary structure models of killer toxins are summarized and grouped by structural similarity. Tertiary structure models are colored by domains as depicted with delta (black), alpha (yellow), gamma (cyan), beta (dark blue), with the exception of K62, which is colored according to the aerolysin core domain (yellow) and N-terminal domain (dark blue). ** The K74 family is included for comparison despite low-confidence predictions of the K74 tertiary structure.

In this manuscript, amino acid sequence homology was used to define killer toxin families that also shared tertiary structure homology, as determined by AlphaFold and MD simulations. This approach identified unexpected structural homology between members of the K1, K2, K45, and K74 families, which share little to no amino acid sequence similarity (Figure 14). Some of the structural models in this group have lower confidence regions, or, in the case of K74, a low overall confidence score that forced the modeling of a sequence homolog that has not been confirmed as a killer toxin (KTS1^Cmal^). However, the shared core tertiary structure, domain organization, and posttranslational modification patterns of both high- and low-confidence models allowed the designation of the K1 superfamily.

All of these K1-like toxin families included proteins with a central α-helix buried within an alpha/beta sandwich. This central α-helix is hydrophobic and predicted to interact with membranes. This suggests a common ionophoric mechanism of antifungal activity, consistent with the proposed pore-forming mechanism of K1 and K2. Intriguingly, the immunity mechanisms of K1 and K2 differ despite the close structural homology and similar methods of intoxication. Structural homology was observed between SMKT and KHS, again supporting a mechanism of membrane attack in which a toxic alpha domain, containing the central hydrophobic α-helix, stably associates with the membranes of intoxicated cells [191]. Other modeling studies of mature K2 have also indicated structural similarity to SMKT, but we have noted higher structural similarities with the Klus family [169].

AlphaFold predicted confident mature toxin models for both Klus and KHR, similar to the empirically determined structure of SMKT [190]. However, the novel domain organization of Klus and KHR differs from that of any previously described toxins, and there are noticeable differences in the more open configuration of Klus and KHR pTox structures. Specifically, the central hydrophobic α-helix is more solvent-exposed, less hydrophobic, and more amphipathic than the analogous α-helices of the K1 superfamily, suggesting that there could be important differences in their antifungal mechanisms, justifying their separation from the K1 superfamily (Figure 14). Despite these differences, the most parsimonious interpretation is that all of the aforementioned toxins share a membrane-attack mechanism involving pore formation by the central hydrophobic α-helix. This prediction is supported by the inherent toxicity of the isolated alpha domains of K1, K2, and other K1-like toxins when expressed in *S. cerevisiae* [115,127,169] as well as mutagenesis experiments showing the importance of the alpha domain for toxicity [132,136,141,156]. However, these data, along with the mechanistic insights from SMKT, have not provided additional evidence on the structure or assembly of K1 superfamily toxin pores, which remains an important area of future study.

Supporting a possible mechanistic divergence of the alpha/beta sandwich killer toxins, the Klus family shows structural homology with toxic lectins that exhibit mechanisms including the formation of β-barrel pores, toxic filaments, and the inhibition of calcium channels. These mechanisms differ from the proposed cation-specific α-helical membrane pores formed by ionophoric killer toxins. Conflicting models of membrane attack have been prominent in the study of Cyt toxins and their homologs, with evidence for membrane-disrupting filaments and membrane-pore formation using electrophysiological methods [244]. Contemporary techniques and advances in synthetic biology will help to determine whether the alpha/beta sandwich is a highly adaptable structural configuration with multiple distinct mechanisms for attacking membranes, or whether there are unifying principles governing the mechanisms of these cytotoxic proteins.

K62 is a structural outlier among *Saccharomyces* killer toxins, placing it in the aerolysin-like pore-forming toxin family, a group more commonly associated with bacterial toxins [226,227,228]. This discovery significantly expands the functional and structural diversity of yeast killer toxins and, more broadly, of killer toxins across fungi. A recent study by Creagh et al. (2025) provides an extensive structural and functional analysis of K62, further supporting its role as an antifungal toxin of the aerolysin family and identifying K62 homologs in pathogenic fungal species [49]. Although there is evidence of horizontal gene transfer of these aerolysin-like toxins from fungi to bacteria and plants, whether they share ancient ancestry with canonical aerolysins or arose through a process of convergent evolution is an open question. Pathogenic fungi have been shown to produce antifungal killer toxins [245] and other protein toxins that enhance tissue invasion and contribute to pathogenicity [246,247]. Therefore, in addition to the more conventional role of killer toxins in niche competition, some of the hundreds of K62 homologs identified in pathogenic fungi could also contribute to fungal virulence.

Beyond the structural findings, this work also highlights the need for a more consistent and informative nomenclature for fungal killer toxins. Historically, toxins have been named somewhat arbitrarily, based on discovery order (e.g., K1, K1L, K2), source organism or strain (e.g., K21, K45, K74), or biological activity (e.g., KHR: killer of heat-resistant yeasts). Moreover, dsRNA-encoded killer toxins have not followed the standardized gene naming conventions used in *S. cerevisiae* and other model fungi. Given the growing number of genome-encoded homologous killer toxin sequences and their clear structural grouping into families, we propose a nomenclature informed by sequence homology, which can be further validated by structural modeling. For instance, we previously designated K1 homologs as “K1-like Killer Toxins” (KKT) [47] and K62 homologs in *Saccharomyces* yeasts as KTA (K62 family; Killer Toxin Aerolysin) [49]. We now propose gene names for the remaining killer toxin families: KTT (K2 family; Killer Toxin Two), KTF (K45 family; Killer Toxin Forty-five), KTS (K74 family; Killer Toxin Seventy-four), and KTN (Klus family; Killer Toxin NaCl-mediated/Klus family). These designations retain a connection to the first-discovered member of each family and reflect their predicted structural and mechanistic classifications. This naming approach offers clarity, reduces redundancy, and allows researchers to draw meaningful functional comparisons across species. It also facilitates a more systematic exploration of killer-toxin biology, enabling insights from well-characterized members, such as K1 and K2, to accelerate our understanding of more recently discovered or poorly understood putative toxins and their homologs.

In summary, this work establishes a framework for the structural classification and functional study of killer toxins in *Saccharomyces* and their homologs. It reveals unexpected evolutionary and mechanistic relationships, proposes a unifying nomenclature, and sets the stage for future experimental studies into a diverse and potent group of eukaryotic toxins.

## 4. Materials and Methods

**Identification of Killer Toxin Homologs.** To identify homologs of killer toxins, publicly available databases were searched using NCBI BLAST. Protein sequences of the *Saccharomyces* killer toxins, K1, K1L, K2, K21, K28, K45, K62, K74, KHR, KHS, and Klus were used as queries for position-specific iterated BLAST (PSI-BLAST) (https://blast.ncbi.nlm.nih.gov) (accessed on 1 December 2025) searches. Each PSI-BLAST analysis was conducted until convergence was reached or 1000 related sequences were retrieved, whichever occurred first. Across all searches, 4437 sequences were obtained. Sequences shorter than 75% or longer than 150% of their respective query sequence length were discounted as potential nonfunctional genes or proteins with significant departure from the length of known killer toxins, removing 1703 potential homologs. PSI-BLAST searches using K62, KHR, and Klus were the only killer toxins that reached the 1000 protein sequence cutoff before convergence and, after filtering, returned 883, 322, and 663 homologs, respectively.

**AlphaFold2 and 3.** Structural models were generated using AlphaFold2 (version 2.2.0) installed on the University of Idaho’s Research Computing and Data Services infrastructure. Models were built with default configuration settings using the singularity.py script. For each single-chain query sequence, we generated five models; the model with the highest pLDDT confidence score was selected for subsequent MD relaxation and analyses. Multimeric complexes were modeled following the same procedure, except that 25 relaxed models were generated for each complex to better sample the conformational space. AlphaFold3-derived structures were obtained directly from the AlphaFold3 Server [178] https://alphafoldserver.com. Sequences acquired from NCBI Genbank were used as inputs into the ‘sequence’ field on AlphaFold Server, while default settings were used.

**Molecular Dynamics.** AlphaFold2 generated models were prepared for MD using a dodecahedral bounding box, solvated with SPC water molecules [248]. Ions were added using Monte Carlo placement to neutralize the system. Each system was energy minimized using the steepest descent until the maximum force within the system reached 1000 kJ/mol/nm. Subsequently, 100 ps of NVT equilibration at 300 K and 100 ps of NPT equilibration at 1 atm were performed, with protein heavy atoms restrained. All simulations were performed using GROMACS v2024.2 [249] molecular dynamics engine with the AMBER99-ILDN [250] forcefield parameters and the SPC water model. The V-rescale [251] thermostat and C-rescale [252] barostat were employed during equilibration and production runs. The LINCS [253] algorithm was used to enforce proper bond lengths involving hydrogen atoms. Long-range electrostatics were computed with the Particle Mesh Ewald [254], and both electrostatic and Van der Waals cutoff distances were set to 1 nm. A 2 fs integration timestep was used throughout. MD simulations were performed for 1 μs. Trajectory analyses were performed using GROMACS analysis tools. Backbone RMSD values were calculated over simulation time, and structural clustering was based on pairwise Root Mean Squared Deviation (RMSD) and until a cutoff yielding ≤10 clusters was obtained.

**FoldX.** Version 5.0 was used to calculate the change in folding stability (ΔΔG) resulting from cysteine-to-alanine point mutations. Calculations were performed using the highest confidence AlphaFold2 generated structural model [158]. Prior to mutation and ΔΔG calculation, the model was subjected to six successive rounds of FoldX RepairPDB command to optimize side-chain conformations, which has been shown to improve accuracy of ΔΔG calculation. Wild-type and mutant structures and their associated ΔΔG values were calculated using the FoldX PositionScan command.

**Software.** Protein secretion signals were predicted using SignalP6 (https://dtu.biolib.com/SignalP-6) (accessed on 1 December 2025) for identifying N-terminal signal peptides and cleavage sites across eukaryotic sequences. Secondary-structure predictions were generated with the PSIPRED suite (https://bioinf.cs.ucl.ac.uk/psipred) (accessed on 1 December 2025), using the PSI-BLAST–based workflow. Topology and transmembrane-helix propensity were further assessed using MemSat, as implemented in the PSIPRED package, to refine predictions of membrane-associated regions. All structural visualization, figure preparation, and qualitative model inspections were performed in PyMOL v2.6 (https://www.pymol.org/) (accessed on 1 December 2025).

## Figures and Tables

**Table 1:** Discovery and general properties of *Saccharomyces* killer toxins. gDNA = Genomic DNA, dsRNA = Double-stranded RNA, *Sc* = *S. cerevisiae*, *Sp* = *S. paradoxus*, β-1,6-G = β-1,6-D-glucan, ‘-’ = unknown, Cell cycle arrest = CC arrest, ** K21 and K66 are close sequence homologs with K21 being discovered first. * depending on assay conditions, ^#^ function predicted by tertiary structure modeling in this study.

Toxin	Gene	Species	Year	Location (Source)	pH	Temp (°C)	Receptor		Mech.
							1°	2°	
K1	dsRNA	*Sc*	1963	- (-)	4.6–4.8	<25 * <42 *	β-1,6-G	Kre1	Ionophore
K2	dsRNA	*Sc*	1978	U.K. (brewery)	4.3	<40	β-1,6-G	Kre1	Ionophore
K28	dsRNA	*Sc/Sp*	1982	- (grape)	5.0	<40	Mannan	Erd2	CC arrest
KHS	gDNA	*Sc/Sp*	1984	Japan (winery)	4.7	<30	-	-	Ionophore ^#^
KHR	gDNA	*Sc*	1984	Japan (winery)	5.2–5.4	<40	-	-	Ionophore ^#^
Klus	dsRNA	*Sc*	2011	Spain (grape)	3.5–5.5	18–28	-	-	Ionophore ^#^
K21 **	dsRNA	*Sp*	2013	U.K. (oak tree)	4.8	15–30	β-1,6-G	-	Ionophore ^#^
K62	dsRNA	*Sp*	2013	U.K. (oak tree)	4.0–4.5	<30	-	-	Ionophore
K74	dsRNA	*Sp*	2013	U.K. (oak tree)	4.3	<28	β-1,6-G	-	Ionophore ^#^
K45	dsRNA	*Sp*	2015	Russia (oak)	-	-	-	-	Ionophore ^#^
K1L	dsRNA	*Sp*	2021	Russia (aspen)	4.5	<30	-	-	Ionophore

## Data Availability

The original contributions presented in this study are included in the article/Supplementary Material. Further inquiries can be directed to the corresponding authors.

## Supplementary Materials

The following supporting information can be downloaded at: https://www.mdpi.com/article/10.3390/toxins18050235/s1, Figure S1. Graphical PSI-BLAST results. Red lines indicate size cutoffs. Figure S2. Killer toxin tertiary structure models colored by pLDDT confidence score. Figure S3. AlphaFold confidence scores and RMSD trajectories of killer toxin tertiary structure models. Figure S4. RMSD trajectories and Ramachandran plots of killer toxin tertiary structure models. Figure S5. Relative positioning of cysteine pairs in all killer toxin tertiary structure models. Figure S6. AlphaFold3 predicted structures of representative K74 homologs. Figure S7. Linear representation of K1 superfamily killer toxins and tertiary structure homologs colored by conserved secondary structure features. Figure S8. Molecular dynamics simulations of mature Klus and KHS. RMSD of backbone atoms of mature Klus (left) and mature KHR (right) compared to the starting conformation over 1 μs MD simulation. Table S1. PDB designations for empirical structures of killer toxins. Table S2. Results of bioinformatic screens using NCBI PSI-BLAST showing the total numbers of homologs of each of the canonical *Saccharomyces* killer toxins identified (highlighted to emphasize larger values in red and orange). Table S3. Confidence statistics and secondary structure composition of AlphaFold models for *Saccharomyces* killer toxins. Table S4. Kex cleavage sites and domain boundary predictions for killer toxins. Table S5. Predicted and experimentally determined disulfide bonds and unpaired cysteines in the modeled structures of *Saccharomyces* killer toxins. Table S6. Central helix buried surface area calculated using PyMol. KHR central helix calculations based on post MD clustered model. Table S7. *∆∆G_fold_* values calculated by FoldX of K1 mutations and their effect on killer toxin function as previously described in the literature. Resi., residue; nd, not done. Table S8. DUF5341-containing proteins identified by sequence homology. Table S9. AlphaFold modeling of K74 homologs and RMSD comparison to KTS1 from *Cadophora malorum*. Table S10. RMSD of ionophoric killer toxin models compared to pilin crystal structures. Measured using the cealign command in PyMOL. Table S11. DALI hits with the K1, K2, and K45 killer toxins and their overlap with 4 or more pilin or pseudopilins (marked by *). Table S12. pLDDT scores for alpha/beta heterodimer models created by AlphaFold. Table S13. RMSD of Klus family killer toxins models to crystal structure matches from DALI. Measured using the cealign command in PyMOL. AF1, AlphaFold2; AF3, AlphaFold3. Table S14. Accession numbers for protein sequences used for tertiary structure modeling.

## Abbreviations

The following abbreviations are used in this manuscript:

MD	Molecular Dynamics
