# Supplementary material for: A Comprehensive Structural and Functional Analysis of Saccharomyces Killer Toxins
Source: Toxins (Basel). 2026 May 20;18(5):235. doi: 10.3390/toxins18050235 (PMC13211469; doi:10.3390/toxins18050235)
Supplement: Supplementary file 1 [file toxins-18-00235-s001.zip › Sup Tables Survey paper_V8.docx]

## Supplementary Tables

| **Name** | **Abbr.** | **Organism** | **PDB ID** | **Residues** |
| --- | --- | --- | --- | --- |
| Salt Mediated Killer Toxin | SMKT | *Millerozyma farinosa* | 1KVD, 1KVE | 222 |
| Killer Protein 4 | KP4 | *Mycosarcoma maydis* | 1KPT | 127 |
| Williopsis mrakii Killer Toxin | WKT | *Williopsis mrakii* | 1WKT | 125 |
| Killer Protein 6 | KP6 | *Mycosarcoma maydis* | 1KP6, 4GVB | 219 |
| Zymoseptoria tritici Killer Protein 6 | Zt-KP6-1 | *Zymoseptoria tritici* | 6QPK, 9GWD | 162 |
| Zymoseptoria tritici Killer Protein 4 | Zt-KP4-1 | *Zymoseptoria tritici* | 8ACX | 96 |

**Table S1.** PDB designations for empirical structures of killer toxins.

|  | **Killer toxin** | | | | | | | | | | |
| --- | --- | --- | --- | --- | --- | --- | --- | --- | --- | --- | --- |
|  | K1 | K1L | K2 | K21 | KHS | K28 | K45 | K62 | K74 | Klus | KHR |
| **Total** | 43 | 44 | 287 | 283 | 282 | 3 | 490 | 1000 | 45 | 1000 | 1000 |
| # too long | 0 | 0 | 2 | 2 | 2 | 0 | 4 | 43 | 0 | 44 | 38 |
| # too short | 23 | 23 | 140 | 136 | 135 | 0 | 92 | 74 | 11 | 313 | 640 |
| Actinomycetes | 0 | 0 | 1 | 1 | 1 | 0 | 0 | 0 | 0 | 0 | 0 |
| Agaricomycetes | 0 | 0 | 0 | 0 | 0 | 0 | 17 | 0 | 0 | 27 | 5 |
| Alphaproteobacteria | 0 | 0 | 0 | 0 | 0 | 0 | 0 | 0 | 0 | 0 | 1 |
| Bacilli | 0 | 0 | 2 | 2 | 2 | 0 | 0 | 36 | 0 | 0 | 0 |
| Chytridiomycetes | 0 | 0 | 0 | 0 | 0 | 0 | 0 | 3 | 0 | 0 | 0 |
| Clostridia | 0 | 0 | 0 | 0 | 0 | 0 | 0 | 1 | 0 | 0 | 0 |
| Dipodascomycetes | 0 | 0 | 0 | 0 | 0 | 0 | 3 | 0 | 2 | 0 | 0 |
| Dothideomycetes | 0 | 0 | 0 | 0 | 0 | 0 | 67 | 49 | 0 | 14 | 1 |
| dsRNA | 6 | 6 | 12 | 12 | 12 | 3 | 1 | 1 | 1 | 1 | 1 |
| Eurotiomycetes | 0 | 0 | 0 | 0 | 0 | 0 | 96 | 21 | 13 | 209 | 48 |
| Exobasidiomycetes | 0 | 0 | 0 | 0 | 0 | 0 | 0 | 1 | 0 | 1 | 0 |
| Lecanoromycetes | 0 | 0 | 0 | 0 | 0 | 0 | 0 | 0 | 0 | 10 | 1 |
| Leotiomycetes | 0 | 0 | 7 | 8 | 8 | 0 | 55 | 23 | 15 | 15 | 1 |
| Lipomycetes | 0 | 0 | 1 | 1 | 1 | 0 | 0 | 2 | 0 | 0 | 0 |
| Lycopodiopsida | 0 | 0 | 0 | 0 | 0 | 0 | 0 | 1 | 0 | 0 | 0 |
| Magnoliopsida | 0 | 0 | 0 | 0 | 0 | 0 | 1 | 0 | 0 | 0 | 0 |
| Myxococcia | 0 | 0 | 0 | 0 | 0 | 0 | 0 | 0 | 1 | 0 | 0 |
| Pichiomycetes | 3 | 3 | 19 | 19 | 19 | 0 | 6 | 65 | 0 | 45 | 29 |
| Polypodiopsida | 0 | 0 | 0 | 0 | 0 | 0 | 0 | 3 | 0 | 0 | 0 |
| Saccharomycetes | 12 | 12 | 102 | 101 | 99 | 0 | 18 | 116 | 2 | 88 | 199 |
| Sordariomycetes | 0 | 0 | 1 | 1 | 1 | 0 | 90 | 553 | 0 | 246 | 34 |
| Tremellomycetes | 0 | 0 | 0 | 0 | 0 | 0 | 0 | 8 | 0 | 1 | 0 |
| Wallemiomycetes | 0 | 0 | 0 | 0 | 0 | 0 | 0 | 0 | 0 | 6 | 2 |

**Table S2.** Results of bioinformatic screens using NCBI PSI-BLAST showing the total numbers of homologs of each of the canonical *Saccharomyces* killer toxins identified (highlighted to emphasize larger values in red and orange).

|  | **pLDDT score** | | | **Clustered 2^o^ structure** | | **Ramachandran favored %** | | **Outliers %** | |
| --- | --- | --- | --- | --- | --- | --- | --- | --- | --- |
| **Toxin** | **Global** | **Min** | **Max** | **Alpha %** | **Beta %** | **relaxed** | **rep frame** | **relaxed** | **rep frame** |
| K1 | 51.35 | 25.24 | 72.3 | 17.50 | 26.07 | 87.77 | 91.70 | 2.88 | 0.83 |
| K1L | 62.93 | 26.21 | 87.72 | 21.66 | 29.94 | 86.54 | 89.53 | 4.17 | 1.81 |
| K2 | 51.01 | 19.42 | 93.48 | 27.35 | 14.09 | 87.33 | 87.31 | 4.44 | 1.21 |
| K21/66 | 67.95 | 28.7 | 93.87 | 30.35 | 20.52 | 87.79 | 90.73 | 4.36 | 1.28 |
| KHS | 60.95 | 25.98 | 90.47 | 26.00 | 20.57 | 85.06 | 91.69 | 3.16 | 0.33 |
| K28 | 34.95 | 18.89 | 60.37 | 51.13 | 2.59 | 85.34 | 90.37 | 4.56 | 1.48 |
| K45 | 62.23 | 23.36 | 86.45 | 21.08 | 20.27 | 85.87 | 89.91 | 4.35 | 2.08 |
| K62 | 82.00 | 26.52 | 97.88 | 10.79 | 41.91 | 92.47 | 94.04 | 1.26 | 0.46 |
| K74 | 35.05 | 22.1 | 61.42 | 28.83 | 6.91 | 83.69 | 89.33 | 4.53 | 1.00 |
| Klus | 65.34 | 21.66 | 95.68 | 21.00 | 19.63 | 87.56 | 91.37 | 2.03 | 4.15 |
| KHR | 58.43 | 20.67 | 83.56 | 15.64 | 31.64 | 89.10 | 88.14 | 5.13 | 1.27 |
| KTS1 (AF3) | 80.60 | 39.12 | 97.65 | 20.10 | 17.10 | 94.58 | 90.82 | 0.60 | 1.31 |
| **Average** | **57.47** | **23.52** | **83.93** | **24.67** | **21.28** | **87.76** | **90.41** | **3.46%** | **1.43** |

**Table S3.** Confidence statistics and secondary structure composition of AlphaFold models for *Saccharomyces* killer toxins.

| **Toxin** | **SignalP** | **PSIPRED signal** | **Delta/**  **Alpha** | **Alpha/**  **Gamma** | **Gamma/**  **Beta** | **Other Kex cleavage sites** |
| --- | --- | --- | --- | --- | --- | --- |
| K1 | 26* | | LLPR44* | VA(RR)147* | VAKR234* | GERK100, YVKR188 |
| K1L | 26 | 10 | FGKR36 | PLKR147 | LTKRR248 | LNKK98, LTRR196 |
| K2 | After 54* | | LVER79 | MAER165 | VGKR268 | IVKR221 |
| K21 | - | 40 | LHKR59 | VHER129 | FTKR240 | LGKR181 |
| KHS | - | 36 | LMRR63 | ATKR132 | FQKR237 | TIKR181 |
| Klus | 23 | 23 | n/a | n/a | n/a | IIKR67, LAKR98, STVK167 |
| KHR | 21 | 16 | n/a | n/a | n/a | ILKR77, VSKR130, CKKK141, ATKR183 |
| K45 | - | 27 | IHRR56 | GFKKR179 | RAKR267 | FAKR97 |
| KTS1 | 23 | 38 | n/a | NNKR116 | LKAR227 | LHRR156 |
| K74 | - | 38 | n/a | WWKR110 | TVKR220 | LHKK102, TSRKR171, |
| K62 | 31 | 23 | n/a | n/a | n/a | LTKR112 |
| K28 | 36* | | LEER49* | IQSR149* | LQ(KR)245* | LYKR192 |
| SMKT | - | 35 | n/a | PVKR83* | PAAR145* | - |

#### **Table S4.** Kex cleavage sites and domain boundary predictions for killer toxins. Cleavage sites are written as the four amino acids (in single-letter nomenclature) occurring on the N-terminal side of the cleavage point. The numbering represents the number of the last amino acid in the sequence of four. n/a, not applicable. *site of posttranslational cleavage that is supported by empirical evidence. Residues in parentheses represent kex1 cleavage of terminal arginine residues.

| **Toxin** | **Empirical prediction** | **Modelled disulfide bonds** | **Unpaired cysteines** |
| --- | --- | --- | --- |
| K1 | C92-C239 | C92-C107, C95-C239, C245-C312 | - |
| K1L | - | C91-C105, C94-C257, C266-C336, | C56, C154, C159, C226 |
| K2 | - | C109-C128, C315-C320, C328-C359, | C25, C45, C300 |
| K21/66 | - | C90-C98, C292-C335 | C29 |
| KHS | - | C94-C102, C292-C336 | - |
| Klus | - | C114-C188, C141-C162, C231-C240 | - |
| Klus mature | - | C114-C188, C141-C162, C231-C240 | - |
| KHR | - | *AF2* (C84-C89, C138-C151, C230-C294, C248-C279)  *MD* (C84-C89, C230-C294, C248-C279) | *AF2* (C143, C189, C219)  *MD* (C138, C143, C151, C189, C219) |
| KHR mature | - | C138-C294, C143-C230, C189-C219, C248-C279 | C151 |
| K45 | - | C123-C137, C311-C367 | C27 |
| KTS1 | - | C69-C82, C326-C334 | C92 |
| K74 | - | C70-C83, C284-C298 | C321, C329 |
| K62 | - | C146-C271, C201-C262, C227-C237 | C269 |
| K28 | C56-C333 | C292-C307, C333-C340 | C56 |

**Table S5.** Predicted and experimentally determined disulfide bonds and unpaired cysteines in the modeled structures of *Saccharomyces* killer toxins.

| **Toxin** | **Area (Å^2^)** | **Solvent exposed (Å^2^)** | **Buried (Å^2^)** | **% buried** | **Central helix sequence** |
| --- | --- | --- | --- | --- | --- |
| K1 | 2026.2 | 328.2 | 1698.0 | 83.8 | CGKQTLALLVSIFVAVTSG |
| K1L | 2406.7 | 220.0 | 2186.8 | 90.9 | CFTAVSEVLKMSIMNAVKISI |
| K2 | 1730.0 | 77.8 | 1652.2 | 95.5 | PTLCAGAYVIGAMS |
| K21/66 | 2181.2 | 367.1 | 1814.1 | 83.2 | APLVNAILSTIVVSVAGGMAW |
| KHS | 2053.3 | 221.5 | 1831.8 | 89.2 | CAPIAGAVLATAAVIVAAVLV |
| Klus | 2336.5 | 950.5 | 1386.0 | 59.3 | YWDIADGVWQAGWDIYRATS |
| KHR | 501.0 | 120.1 | 380.5 | 76.0 | TDRI/ VYNLVSGITDRIKEAT* |
| K45 | 1301.0 | 142.9 | 1158.1 | 89.0 | DCVVGAVDTGVTLG |
| KTS1 | 1972.2 | 251.0 | 1721.2 | 87.3 | KAACQQSVLETGVCMGLSV |

#### **Table S6.** Central helix buried surface area calculated using PyMol. KHR central helix calculations based on post MD clustered model.

| **Resi.** | **#** | **Mutation** | **ΔΔG_folding_** | **Whole cell killing** | **Sphero. killing** | **Toxin Secretion** | **Cell wall bind** | **Immune** | **Ref.** |
| --- | --- | --- | --- | --- | --- | --- | --- | --- | --- |
| G | 83 | D | 2.669 | <0.01 | - | 66 | + | - | (141) |
| C | 92 | Y | 9.133 | <0.02 | - | 65 | - | - | (141) |
| D | 101 | R | 0.517 | <0.05 | + | 3 | - | + | (141) |
| L | 114 | K | 2.394 | <0.01 | - | 59 | - | - | (141) |
| L | 115 | P | 6.449 | <0.01 | - | 41 | - | - | (141) |
| S | 124 | P | 0.012 | <0.01 | - | 21 | - | - | (141) |
| I | 129 | R | -0.157 | 10 | +/- | 80 | +/- | +/- | (141) |
| D | 140 | R | 0.373 | 10 | +/- | 80 | +/- | +/- | (141) |
| P | 237 | S | 3.574 | <0.1 | + | 68 | - | + | (141) |
| G | 264 | L | 3.861 | 75 | + | 75 | + | + | (141) |
| C | 312 | L | 6.321 | 50 | + | 57 | + | + | (141) |
| C | 92 | S | 3.191 | - | - | - | nd | + | (132) |
| C | 95 | S | 5.767 | - | - | - | nd | - | (132) |
| C | 107 | S | 4.236 | - | - | - | nd | - | (132) |
| C | 239 | S | 6.086 | - | - | nd | nd | + | (132) |
| C | 248 | S | 6.987 | - | + | + | nd | + | (132) |
| C | 312 | S | 6.277 | - | - | + | nd | + | (132) |
| T | 191 | P | 3.086 | 80 | nd | 80* | nd | + | (136) |
| R | 188 | A | 1.476 | 25 | nd | nd | nd | + | (136) |
| N | 181 | K | -0.359 | 40 | nd | 40* | nd | + | (136) |
| I | 151 | H | 1.640 | 100 | nd | 100* | nd | + | (136) |
| V | 85 | T | 0.624 | +/- | nd | + | nd | +/- | (156) |
| V | 116 | T | 2.156 | +/- | nd | + | nd | + | (156) |
| D | 101 | R | 0.517 | - | + | +/- | nd | + | (156) |

**Table S7.** *∆∆G_fold_* values calculated by FoldX of K1 mutations and their effect on killer toxin function as previously described in the literature. Resi., residue; nd, not done.

| **Accession** | **Name** | **Tax Name** | **aa** | **Matched region** | **C-term?** |
| --- | --- | --- | --- | --- | --- |
| A0A0L0P143 | Uncharacterized protein | *Candidozyma auris* | 324 | 206..282,288..322 | Y |
| A0A0L8VR10 | Uncharacterized protein | *Saccharomyces boulardii* | 175 | 3..122 | N |
| A0A0L8VS32 | YER187Wp protein | *Saccharomyces boulardii* | 350 | 185..344 | Y |
| A0A1D9QD80 | Uncharacterized protein | *Sclerotinia sclerotiorum* | 298 | 198..296 | Y |
| A0A1E3NFD2 | Uncharacterized protein | *Pichia membranifaciens* | 304 | 155..301 | Y |
| A0A1E3NZB6 | Uncharacterized protein | *Wickerhamomyces anomalus* | 135 | 4..131 | N |
| A0A1E3PUM7 | Uncharacterized protein | *Lipomyces starkeyi* | 322 | 197..285 | Y |
| A0A1E3QRX6 | Uncharacterized protein | *Babjeviella inositovora* | 213 | 98..197 | Y |
| A0A1E3QTL6 | Uncharacterized protein | *Babjeviella inositovora* | 201 | 50..194 | Y |
| A0A1X7QXL0 | Flo11 domain-containing protein | *Maudiozyma saulgeensis* | 309 | 157..302 | Y |
| A0A1X7R3D2 | Uncharacterized protein | *Maudiozyma saulgeensis* | 302 | 174..301 | Y |
| A0A1X7R7E7 | Uncharacterized protein | *Maudiozyma saulgeensis* | 451 | 348..450 | Y |
| A0A1X7RAH9 | Cell wall protein | *Maudiozyma saulgeensis* | 320 | 174..319 | Y |
| A0A1X7RB12 | Uncharacterized protein | *Maudiozyma saulgeensis* | 448 | 364..447 | Y |
| A0A291FEA7 | K2 killer toxin | M2-4 satellite | 362 | 239..360 | Y |
| A0A2D1CIW1 | K21 killer preprotoxin | M21 satellite | 346 | 190..343 | Y |
| A0A2H1A5H9 | Uncharacterized protein | *Candidozyma auris* | 313 | 195..271,277..311 | Y |
| A0A3E2GRT2 | Uncharacterized protein | *Scytalidium lignicola* | 264 | 162..262 | Y |
| A0A3G2M388 | K66 protein | M66 satellite | 346 | 190..343 | Y |
| A0A6C1DQI2 | Uncharacterized protein | *Saccharomyces pastorianus* | 350 | 185..344 | Y |
| A0A6C1EAJ4 | Uncharacterized protein | *Saccharomyces pastorianus* | 235 | 74..228 | Y |
| A0A7G3Z9N0 | Uncharacterized protein | *Torulaspora globosa* | 61 | 20..54 | N |
| A0A8B8UQG8 | Uncharacterized protein | *Saccharomyces paradoxus* | 407 | 242..401 | Y |
| A0A8F3AG53 | Uncharacterized protein | *Candidozyma auris* | 324 | 206..282,288..322 | Y |
| A0A8H2VEL1 | Cell wall protein | *Maudiozyma barnettii* | 295 | 167..294 | Y |
| A0A8H2VHB5 | Uncharacterized protein | *Maudiozyma barnettii* | 442 | 317..441 | Y |
| A0A8H2VJW5 | Cell wall protein | *Maudiozyma barnettii* | 318 | 172..317 | Y |
| A0A8H2ZGN9 | Uncharacterized protein | *Maudiozyma barnettii* | 150 | 47..147 | Y |
| A0A8H2ZHH3 | Uncharacterized protein | *Maudiozyma barnettii* | 366 | 204..360 | Y |
| A0A8H2ZK67 | Uncharacterized protein | *Maudiozyma barnettii* | 449 | 363..448 | Y |
| A0A8H2ZRB1 | 65605ac8-9276-40ca-8d7f-4e054c7d8f35 | *Sclerotinia trifoliorum* | 298 | 200..296 | Y |
| A0A8H7TB95 | Uncharacterized protein | *Cadophora malorum* | 300 | 205..298 | Y |
| A0A9P6VFC7 | Uncharacterized protein | *Hyphodiscus hymeniophilus* | 316 | 190..314 | Y |
| A0A9P6VZZ3 | Uncharacterized protein | *Maudiozyma exigua* | 259 | 128..248 | Y |
| A0A9P6W2C2 | Uncharacterized protein | *Maudiozyma exigua* | 363 | 202..357 | Y |
| A0A9P6W7S6 | Uncharacterized protein | *Maudiozyma exigua* | 451 | 357..450 | Y |
| A0A9P6WF38 | Cell wall protein | *Maudiozyma exigua* | 323 | 177..322 | Y |
| A0A9P7BA26 | Uncharacterized protein | *Maudiozyma exigua* | 294 | 165..290 | Y |
| A0A9P7BC61 | Methyl-accepting transducer domain-containing protein | *Maudiozyma exigua* | 516 | 385..515 | Y |
| A0A9P7BCG2 | Uncharacterized protein | *Maudiozyma exigua* | 110 | 45..94 | Y |
| A0A9P8PYQ9 | Uncharacterized protein | *Wickerhamomyces mucosus* | 310 | 146..305 | Y |
| A0A9X0DGA6 | Uncharacterized protein | *Sclerotinia nivalis* | 298 | 200..296 | Y |
| A0AAD5FWM1 | Uncharacterized protein | *Candida theae* | 80 | 3..73 | N |
| A0AAJ8W4I4 | Uncharacterized protein | *Candida parapsilosis* | 385 | 223..379 | Y |
| A0AAN7W6T7 | Uncharacterized protein | *Arxiozyma heterogenica* | 334 | 228..332 | Y |
| A0AAN7WJU1 | Uncharacterized protein | *Arxiozyma heterogenica* | 309 | 203..307 | Y |
| A0AAN7WJV8 | Uncharacterized protein | *Arxiozyma heterogenica* | 318 | 227..291 | Y |
| A0AAN7WN43 | Uncharacterized protein | *Arxiozyma heterogenica* | 233 | 112..179 | N |
| A0AAN7WN76 | Uncharacterized protein | *Arxiozyma heterogenica* | 443 | 181..331 | N |
| A0AAV5QE32 | Uncharacterized protein | *Saccharomycopsis crataegensis* | 66 | 22..59 | N |
| A0AAV5QEV5 | Uncharacterized protein | *Saccharomycopsis crataegensis* | 321 | 198..297 | Y |
| A0AAV5RC26 | Uncharacterized protein | *Pichia kluyveri* | 306 | 153..302 | Y |
| A0AAV5S0A0 | Uncharacterized protein | *Maudiozyma humilis* | 298 | 165..297 | Y |
| A0AAV5S243 | Uncharacterized protein | *Maudiozyma humilis* | 499 | 408..498 | Y |
| A0AAV5S3I0 | Uncharacterized protein | *Maudiozyma humilis* | 501 | 373..500 | Y |
| A0AAV5S4C9 | Uncharacterized protein | *Maudiozyma humilis* | 326 | 183..325 | Y |
| A0AAV5S5V7 | Uncharacterized protein | *Maudiozyma humilis* | 501 | 371..500 | Y |
| A6ZRE3 | YER187Wp-like protein | *Saccharomyces cerevisiae* | 350 | 185..344 | Y |
| B3LHV3 | Uncharacterized protein | *Saccharomyces cerevisiae* | 175 | 3..122 | N |
| B3LRU6 | Uncharacterized protein | *Saccharomyces cerevisiae* | 141 | 2..135 | N |
| B5FV67 | KLLA0C19327p | *Kluyveromyces lactis* | 117 | 4..107 | N |
| B5RT98 | 1,3-beta-glucanosyltransferase | *Debaryomyces hansenii* | 207 | 67..205 | Y |
| B5VHW8 | YER187Wp-like protein | *Saccharomyces cerevisiae* | 237 | 72..231 | Y |
| B5VI83 | YGL262Wp-like protein | *Saccharomyces cerevisiae* | 175 | 3..122 | N |
| C5M1X0 | Uncharacterized protein | *Candida tropicalis* | 255 | 121..253 | Y |
| C7GUZ0 | YER187W-like protein | *Saccharomyces cerevisiae* | 141 | 2..135 | N |
| C8Z6R6 | EC1118_1E31_0001p | *Saccharomyces cerevisiae* | 141 | 2..135 | N |
| E9P9Y8 | M2-1 protein | *Saccharomyces cerevisiae* | 362 | 239..360 | Y |
| E9PA29 | K2 killer toxin | *Saccharomyces cerevisiae* | 362 | 239..360 | Y |
| G0VBZ5 | Uncharacterized protein | *Naumovozyma castellii* | 50 | 1..47 | N |
| G0W5M8 | Uncharacterized protein | *Naumovozyma dairenensis* | 96 | 2..46 | N |
| G0WFL7 | Uncharacterized protein | *Naumovozyma dairenensis* | 357 | 193..350 | Y |
| G0WFL9 | Uncharacterized protein | *Naumovozyma dairenensis* | 144 | 2..137 | N |
| G2WD74 | K7_Yer187wp | *Saccharomyces cerevisiae* | 141 | 2..135 | N |
| G8BHI6 | Uncharacterized protein | *Candida parapsilosis* | 385 | 223..379 | Y |
| G8BMG7 | Uncharacterized protein | *Tetrapisispora phaffii* | 128 | 4..128 | N |
| G8BQ85 | Uncharacterized protein | *Tetrapisispora phaffii* | 235 | 68..229 | Y |
| G8BS31 | Uncharacterized protein | *Tetrapisispora phaffii* | 380 | 218..373 | Y |
| G8BX89 | Uncharacterized protein | *Tetrapisispora phaffii* | 326 | 189..326 | Y |
| G8C2G5 | Uncharacterized protein | *Tetrapisispora phaffii* | 344 | 182..342 | Y |
| H0GFI5 | YER187W-like protein | *Saccharomyces cerevisiae* | 141 | 2..135 | N |
| H0GFX1 | YGL262W-like protein | *Saccharomyces cerevisiae* | 359 | 198..352 | Y |
| H2AM48 | Uncharacterized protein | *Kazachstania africana* | 236 | 74..230 | Y |
| H2AQH1 | Uncharacterized protein | *Kazachstania africana* | 216 | 60..210 | Y |
| H2ATF3 | Uncharacterized protein | *Kazachstania africana* | 267 | 112..265 | Y |
| H2AUW4 | Uncharacterized protein | *Kazachstania africana* | 323 | 210..321 | Y |
| H2AXE1 | Uncharacterized protein | *Kazachstania africana* | 150 | 44..146 | Y |
| H2AXE3 | C-type lectin domain protein | *Kazachstania africana* | 323 | 225..320 | Y |
| H8X6K5 | Uncharacterized protein | *Candida orthopsilosis* | 52 | 2..44 | N |
| I2GWM8 | Uncharacterized protein | *Henningerozyma blattae* | 367 | 209..364 | Y |
| I2H2I0 | Uncharacterized protein | *Henningerozyma blattae* | 385 | 217..384 | Y |
| I2H3P0 | Uncharacterized protein | *Henningerozyma blattae* | 362 | 212..360 | Y |
| I2H5S6 | Uncharacterized protein | *Henningerozyma blattae* | 356 | 209..355 | Y |
| I2H689 | Uncharacterized protein | *Henningerozyma blattae* | 365 | 207..363 | Y |
| I2H6N7 | Uncharacterized protein | *Henningerozyma blattae* | 354 | 202..353 | Y |
| N1P3M7 | Uncharacterized protein | *Saccharomyces cerevisiae* | 175 | 3..122 | N |
| N1P5I2 | Uncharacterized protein | *Saccharomyces cerevisiae* | 141 | 2..135 | N |
| P40102 | YER187W | *Saccharomyces cerevisiae* | 141 | 2..135 | N |
| P53054 | YGL262W | *Saccharomyces cerevisiae* | 175 | 3..122 | N |
| Q6BQY9 | DEHA2E01254p | *Debaryomyces hansenii* | 56 | 5..32 | N |
| Q6CX15 | KLLA0A12045p | *Kluyveromyces lactis* | 387 | 223..380 | Y |
| Q751P8 | AGL359Cp | *Eremothecium gossypii* | 375 | 205..365 | Y |
| Q87020 | K2 killer toxin | M2 satellite | 362 | 239..360 | Y |
| R9XGX4 | AaceriAGL359Cp | *Ashbya aceri* | 374 | 205..364 | Y |

**Table S8.** DUF5341-containing proteins identified by sequence homology

| **Accession** | **Species** | **pTM** | **% ID** | **Group** | **Gamma/beta cleavage** | **RMSD KTS1** |
| --- | --- | --- | --- | --- | --- | --- |
| KAH7395541 | *Cadophora sp.* | 0.84 | 16.83 | 1 | LKAR227 | 1.16 |
| KAI6714648 | *D. mali* | 0.84 | 15.94 | 1 | LQAR227 | 1.05 |
| PBP21118 | *D. rosae* | 0.84 | 13.25 | 1 | VEAR230 | 1.07 |
| KAG4413772 | *C. malorum* | 0.83 | 16.96 | 1 | LKAR226 | 1.19 |
| XP_018065141 | *M. scopiformis* | 0.83 | 16.40 | 2 | LAKR212 | 1.20 |
| PBP20030 | *D. rosae* | 0.83 | 13.65 | 1 | LEAR230 | 1.08 |
| XP_025506929 | *A. aculeatinus* | 0.82 | 16.57 | 3 | HQRR214 | 1.45 |
| KAJ5673074 | *P. longicatenatum* | 0.80 | 17.63 | 3 | MSRR217 | 1.42 |
| XP_008087614 | *G. lozoyensis* | 0.80 | 12.67 | 2 | LVER240 | 1.36 |
| XP_025522666 | *A. japonicus* | 0.80 | 17.66 | 3 | LRRR217 | 1.56 |
| RMJ21078 | *Aspergillus sp.* | 0.80 | 17.24 | 3 | WLKR240 | 1.30 |
| OJJ75601 | *A. brasiliensis* | 0.79 | 16.27 | 3 | HQRR217 | 1.48 |
| XP_025385908 | *A. eucalypticola* | 0.79 | 16.92 | 3 | HQRR215 | 1.43 |
| XP_025575136 | *A. ibericus* | 0.78 | 17.86 | 3 | LQRR221 | 1.68 |
| XP_020051169 | *A. aculeatis* | 0.77 | 17.86 | 3 | LQRR221 | 1.76 |
| KAG4432325 | *Cadophora sp.* | 0.76 | 13.81 | 1 | LTPR222 | 1.02 |
| KAF8860732 | *A. macrosclerotiorum* | 0.75 | 14.29 | 2 | IKGR189 | 1.12 |
| XP_056952645 | *P. manginii* | 0.75 | 16.81 | 3 | MTRR216 | 1.50 |
| KAI9052667 | *D. brunnea f sp.* | 0.73 | 13.6 | 1 | LKAR238 | 1.22 |
| XP_025442839 | *A. brunneviolaceus* | 0.73 | 16.57 | 3 | HQRR215 | 1.60 |
| KAJ5623171 | *P. lividum* | 0.72 | 17.48 | 3 | MNRR219 | 1.72 |
| OWP04658 | *D. corinariae* | 0.31 | 16.10 | 1 | LQRR193 | 18.83 |
| OOF98769 | *A. carbonarius* | 0.30 | 17.47 | 3 | LQRR193 | nd |
| CZR64371 | *P. subalpina* | 0.26 | 13.84 | 2 | KDRR245 | nd |
| CZT01702 | *R. graminicola* | 0.24 | 15.83 | 1 | NIKR233 | nd |
| SCU84484 | *L. myersii* | 0.2 | 33.43 | U | TNKR223 | nd |
| XP_007291920 | *D. brunnea* | 0.19 | 14.49 | 1 | LKAR237 | nd |
| XP_003956103 | *K. africana* | 0.18 | 27.46 | U | LVKR218 | nd |
| KAA8900892 | *T. ciferrii* | 0.17 | 14.83 | U | TNKR214 | nd |
| XP_058350194 | *C. gregata* | 0.81 | 16.37 | 1 | LKAR238 | 1.22 |
| MAD84956 | *D. bacterium* | 0.68 | 16.67 | U | VSKR239 | 1.10 |

**Table S9.** AlphaFold modeling of K74 homologs and RMSD comparison to KTS1 from *Cadophora malorum*.

|  |  | K1 | K1L | K2 | K21 | KHS | K45 | KTS1 | KHR | Klus | 2ret | 4noa | 3jyz | 7o6y | 5bw0 | 4d40 |
| --- | --- | --- | --- | --- | --- | --- | --- | --- | --- | --- | --- | --- | --- | --- | --- | --- |
| **AF2****+****MD** | K1 | 0.0 |  |  |  |  |  |  |  |  |  |  |  |  |  |  |
|  | K1L | 5.0 | 0.0 |  |  |  |  |  |  |  |  |  |  |  |  |  |
|  | K2 | 11.1 | 6.1 | 0.0 |  |  |  |  |  |  |  |  |  |  |  |  |
|  | K21 | 6.5 | 6.0 | 6.6 | 0.0 |  |  |  |  |  |  |  |  |  |  |  |
|  | KHS | 6.6 | 7.3 | 6.9 | 6.1 | 0.0 |  |  |  |  |  |  |  |  |  |  |
|  | K45 | 5.7 | 5.8 | 5.1 | 5.1 | 6.4 | 0.0 |  |  |  |  |  |  |  |  |  |
|  | KTS1 | 5.7 | 6.5 | 8.1 | 6.3 | 6.2 | 6.8 | 0.0 |  |  |  |  |  |  |  |  |
|  | KHR | 5.4 | 5.2 | 9.3 | 5.3 | 6.3 | 8.2 | 5.1 | 0.0 |  |  |  |  |  |  |  |
|  | Klus | 4.8 | 5.2 | 6.3 | 4.6 | 4.5 | 9.8 | 5.8 | 6.8 | 0.0 |  |  |  |  |  |  |
| **PDB** | 2ret | 6.3 | 7.5 | 5.7 | 5.3 | 5.3 | 7.5 | 5.5 | 5.5 | 9.3 | 0.0 |  |  |  |  |  |
|  | 4noa | 4.8 | 5.6 | 6.3 | 5.5 | 5.4 | 5.4 | 5.7 | 10.0 | 9.6 | 3.9 | 0.0 |  |  |  |  |
|  | 3jyz | 4.8 | 5.8 | 6.0 | 5.4 | 5.4 | 6.2 | 5.5 | 5.6 | 7.8 | 6.1 | 4.3 | 0.0 |  |  |  |
|  | 7o5y | 9.2 | 6.9 | 7.5 | 5.9 | 6.5 | 6.7 | 6.2 | 5.4 | 4.5 | 4.7 | 4.3 | 6.3 | 0.0 |  |  |
|  | 5bw0 | 6.5 | 9.2 | 5.9 | 5.6 | 5.1 | 7.2 | 5.8 | 8.4 | 8.8 | 2.3 | 4.8 | 6.5 | 4.0 | 0.0 |  |
|  | 4d40 | 6.4 | 8.7 | 8.2 | 6.4 | 5.1 | 6.2 | 6.0 | 5.7 | 9.3 | 4.9 | 2.5 | 3.9 | 4.9 | 4.9 | 0.0 |
|  | 1kvd | 4.6 | 3.9 | 6.1 | 4.4 | 4.6 | 4.2 | 5.4 | 4.2 | 3.4 | 4.7 | 5.8 | 5.3 | 5.9 | 4.5 | 4.6 |

**Table S10.** RMSD of ionophoric killer toxin models compared to pilin crystal structures. Measured using the cealign command in PyMOL.

| **PDB** | **# killer toxins** | **Description** |
| --- | --- | --- |
| 2ret* | 6 | The crystal structure of a binary complex of two pseudopilins: EpsI and EpsJ from the Type 2 Secretion System of *Vibrio vulnificus* |
| 4noa* | 4 | Truncated minor pilin PilE from *Pseudomonas aeruginosa* |
| 3jyz* | 4 | Crystal structure of *Pseudomonas aeruginosa* (strain: Pa110594) typeIV pilin in space group P41212 |
| 7o5y* | 4 | PilA minor pilin of *Streptococcus sanguinis* type IV pili |
| 6grf | 4 | Crystal structure of the tandem DUF26 ectodomain from the *Arabidopsis thaliana* cysteine-rich receptor-like protein PDLP8. |
| 1uyn | 4 | Translocator domain of autotransporter NalP from *Neisseria meningitidis* |
| 5bw0* | 4 | The crystal structure of minor pseudopilin binary complex of XcpV and XcpW from the Type 2 secretion system of *Pseudomonas aeruginosa* |
| 4q6u | 4 | Crystal structure of a putative uncharacterized protein from *Mycobacterium tuberculosis* |
| 4d40* | 4 | High-Resolution Structure of a Type IV Pilin from *Shewanella oneidensis* |

**Table S11.** DALI hits with the K1, K2, and K45 killer toxins and their overlap with 4 or more pilin or pseudopilins (marked by *).

|  | **K1** | **K1L** | **K2** | **K21** | **KHS** | **K45** | **KTS1** | **Klus** | **KHR** |
| --- | --- | --- | --- | --- | --- | --- | --- | --- | --- |
| Average | 40.1 | 48.5 | 41.1 | 40.2 | 45.5 | 43.5 | 58.7 | **72.4** | **71.0** |
| Max | 58.6 | 81.4 | 66.1 | 74.7 | 81.2 | 71.5 | 96.9 | **96.3** | **89.5** |
| Min | 19.3 | 18.2 | 22.5 | 18.7 | 17.3 | 20.8 | 19.8 | **31.8** | **39.9** |

#### **Table S12.** pLDDT scores for alpha/beta heterodimer models created by AlphaFold.

|  |  | Klus pptox | KHR pptox | Mature Klus | Mature KHR | SMKT | KP4 | Gnk-2 | LDL | Zt-KP4 | Y3 | VVA2 |
| --- | --- | --- | --- | --- | --- | --- | --- | --- | --- | --- | --- | --- |
| **AF1****+****MD** | Klus ppTox | 0.0 |  |  |  |  |  |  |  |  |  |  |
|  | KHR ppTox | 6.8 | 0.0 |  |  |  |  |  |  |  |  |  |
|  | Mature Klus | 3.5 | 4.7 | 0.0 |  |  |  |  |  |  |  |  |
|  | Mature KHR | 5.2 | 4.6 | 4.6 | 0.0 |  |  |  |  |  |  |  |
| **PDB** | SMKT | 4.1 | 4.5 | 3.2 | 4.1 | 0.0 |  |  |  |  |  |  |
|  | Kp4 | 5.4 | 5.8 | 4.8 | 5.9 | 5.4 | 0.0 |  |  |  |  |  |
|  | Gnk-2 | 4.5 | 4.5 | 4.7 | 4.9 | 4.5 | 5.4 | 0.0 |  |  |  |  |
|  | LDL | 4.8 | 3.8 | 3.6 | 3.7 | 4.3 | 4.6 | 4.1 | 0.0 |  |  |  |
|  | Zt- kp4 | 4.9 | 5.0 | 4.7 | 6.2 | 8.2 | 5.0 | 5.5 | 3.8 | 0.0 |  |  |
|  | Y3 | 5.1 | 5.9 | 3.9 | 5.2 | 4.8 | 4.2 | 4.2 | 3.8 | 4.0 | 0.0 |  |
|  | VVA2 | 7.1 | 6.5 | 6.8 | 4.4 | 7.0 | 5.4 | 5.8 | 5.0 | 5.8 | 5.9 |  |
| **AF3** | SMKT ppTox | 3.6 | 4.2 | 4.8 | 6.5 | 4.2 | 5.7 | 6.1 | 7.9 | 4.7 | 7.7 | 6.4 |

#### **Table S13.** RMSD of Klus family killer toxins models to crystal structure matches from DALI. Measured using the cealign command in PyMOL. AF1, AlphaFold2; AF3, AlphaFold3.

| **Killer toxin** | **Accession number** |
| --- | --- |
| Klus | ADG64740.1 |
| K1 | NP_044402.1 |
| K1L | QQX23408.1 |
| K2 | AAB21819.1 |
| K28 | 2205370A |
| K62 | ATN38496.1 |
| K66 | AYN80721.1 |
| KHR | CAY80481.1 |
| KHS | EDN63163.1 |
| K74 | ATN38272.1 |
| K45 | ATN38271.1 |

**Table S14.** Accession numbers for protein sequences used for tertiary structure modeling.
