# Supplementary material for: A Comprehensive Structural and Functional Analysis of Saccharomyces Killer Toxins
Source: Toxins (Basel). 2026 May 20;18(5):235. doi: 10.3390/toxins18050235 (PMC13211469; doi:10.3390/toxins18050235)
Supplement: Supplementary file 1 [file toxins-18-00235-s001.zip › Supplementary Figures_mdpi submission_V2.pdf]

Supplementary Figures

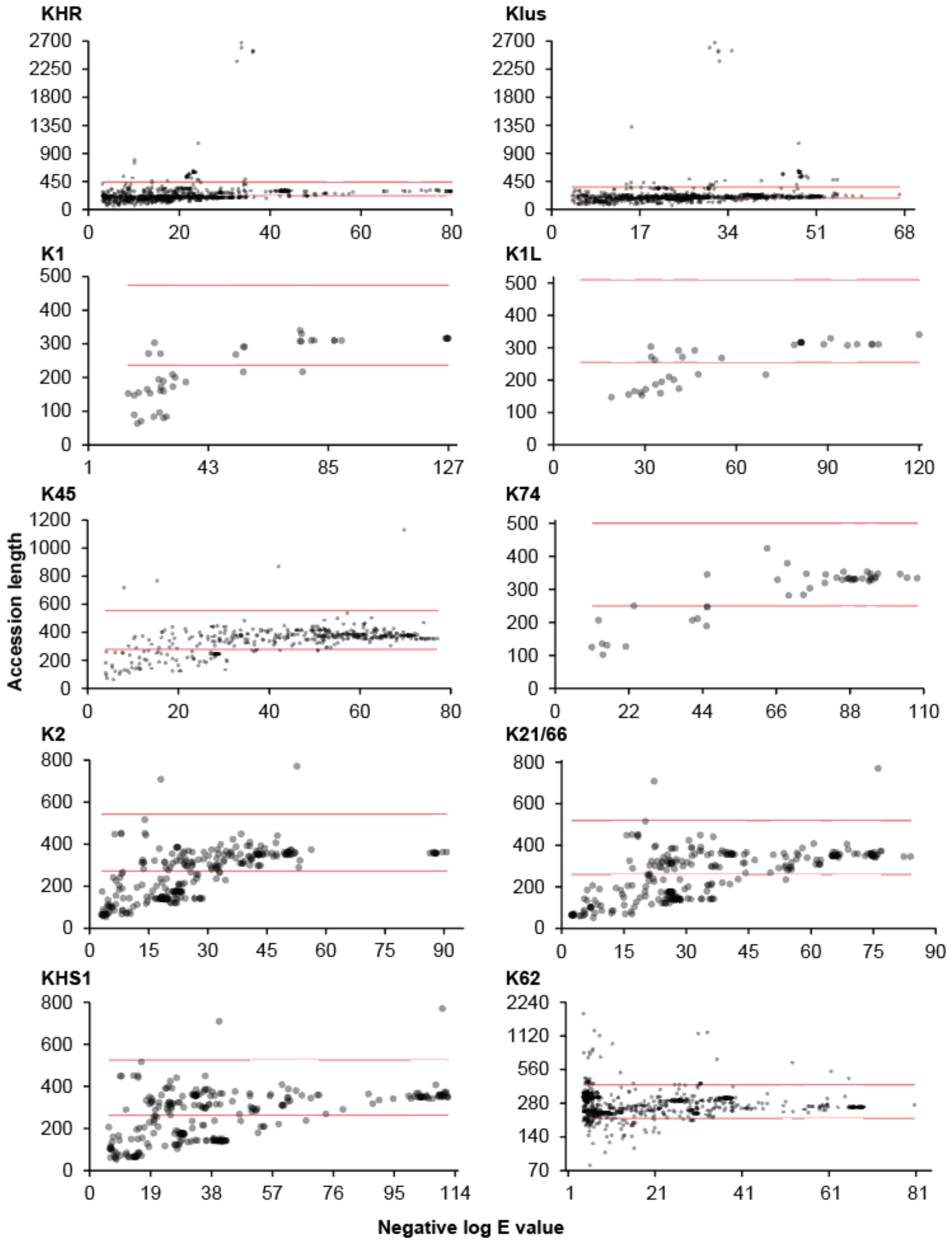

Figure S1. Graphical PSI-BLAST results. Red lines indicate size cutoffs.

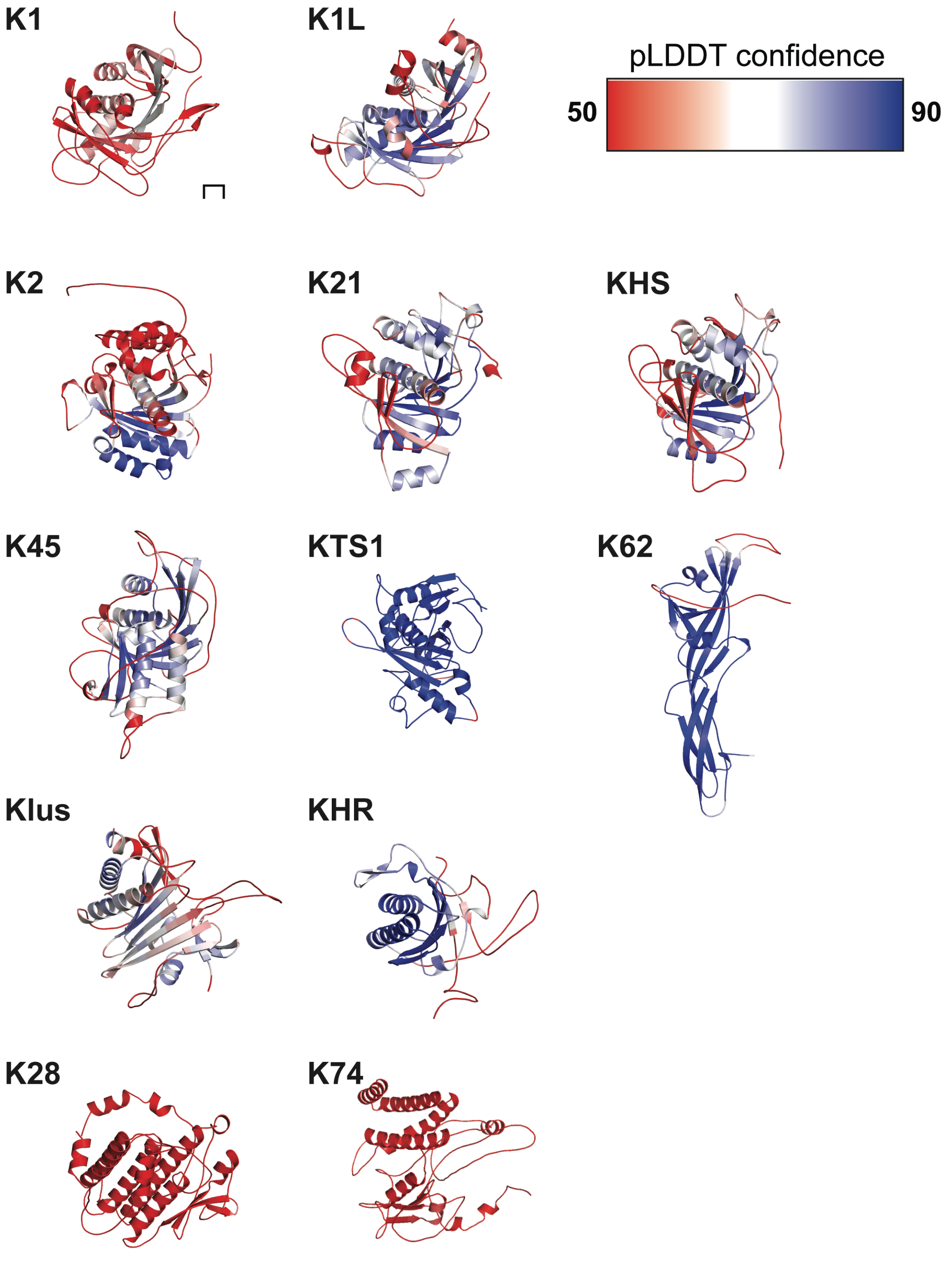

Figure S2. Killer toxin tertiary structure models colored by pLDDT confidence score. Top relaxed AlphaFold2 models colored by pLDDT confidence, with blues being more confident structural predictions.

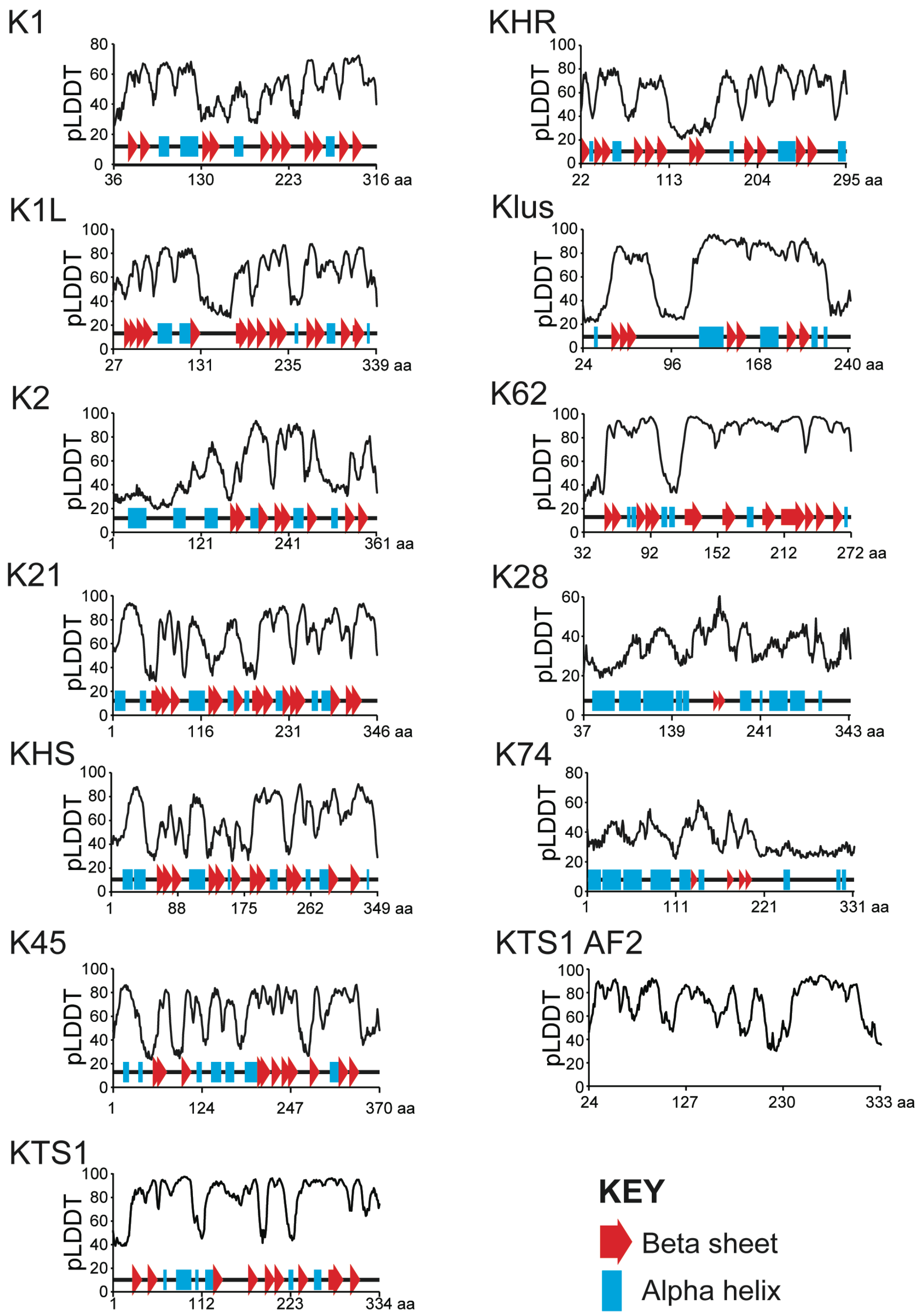

Figure S3. AlphaFold confidence scores and RMSD trajectories of killer toxin tertiary structure models. Predicted local distance difference test (pLDDT) scores from AlphaFold2 models of all *Saccharomyces* killer toxins, showing the confidence in the top-ranked model.

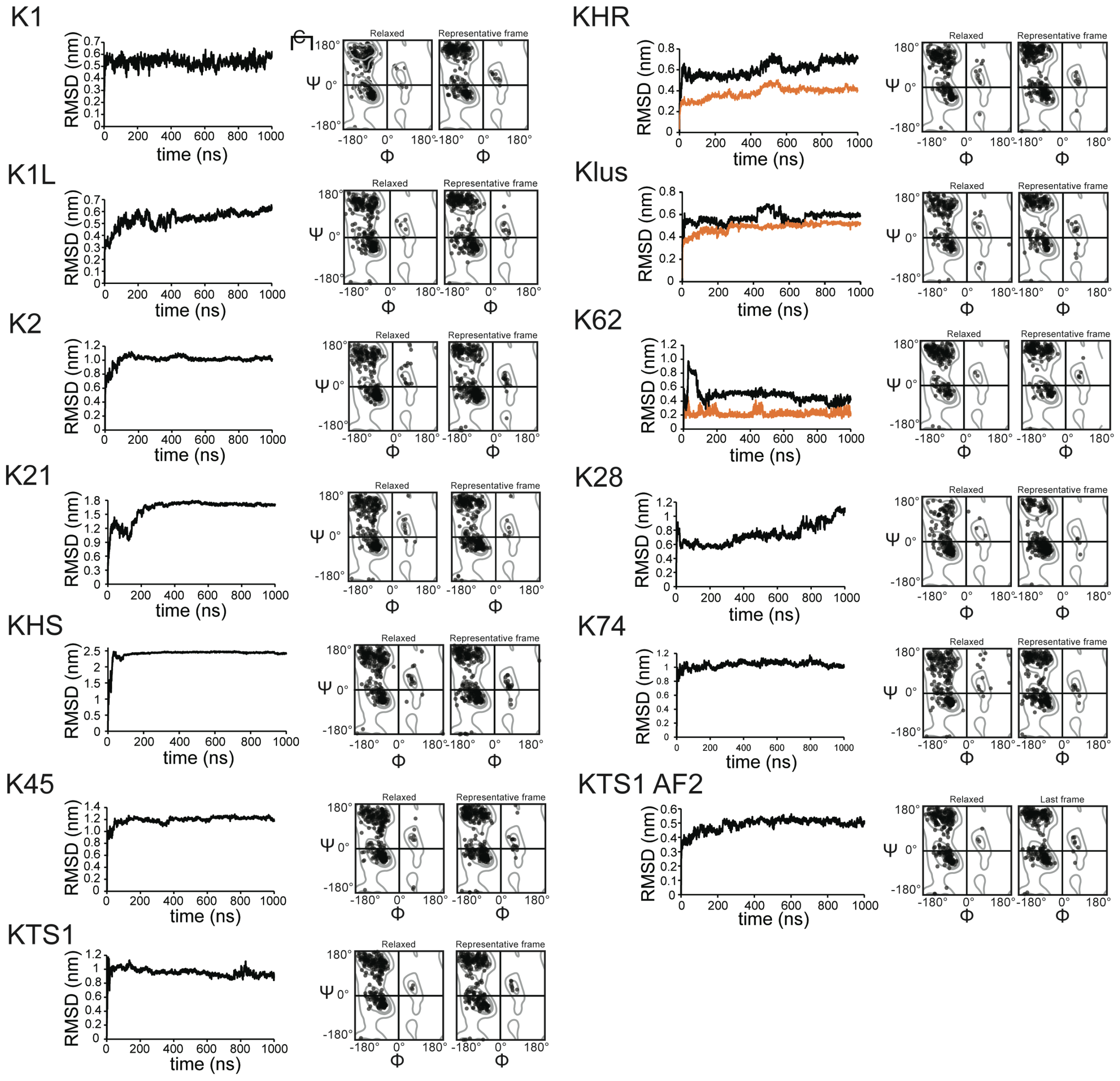

Figure S4. RMSD trajectories and Ramachandran plots of killer toxin tertiary structure models. Protein RMSD over 1 μs molecular dynamics simulation. GROMACS was used to generate alignments of each snapshot to the structure at 0 ns. Orange RMSD traces in KHR, Klus, and K62 have 111 to 161 residues removed, 15 N-terminal residues removed and 112 N-terminal residues removed, demonstrating the RMSD fluctuations are due to N-terminal flexibility. Orange RMSD traces for KHR and Klus represent the mature alpha/beta domain models, while for K62 it represents the C-terminal core aerolysin domain. Ramachandran plots of general residues (non-proline/glycine) generated by SWISS structure assessment tool before (AlphaFold2's amber relaxed output) and after MD simulation.

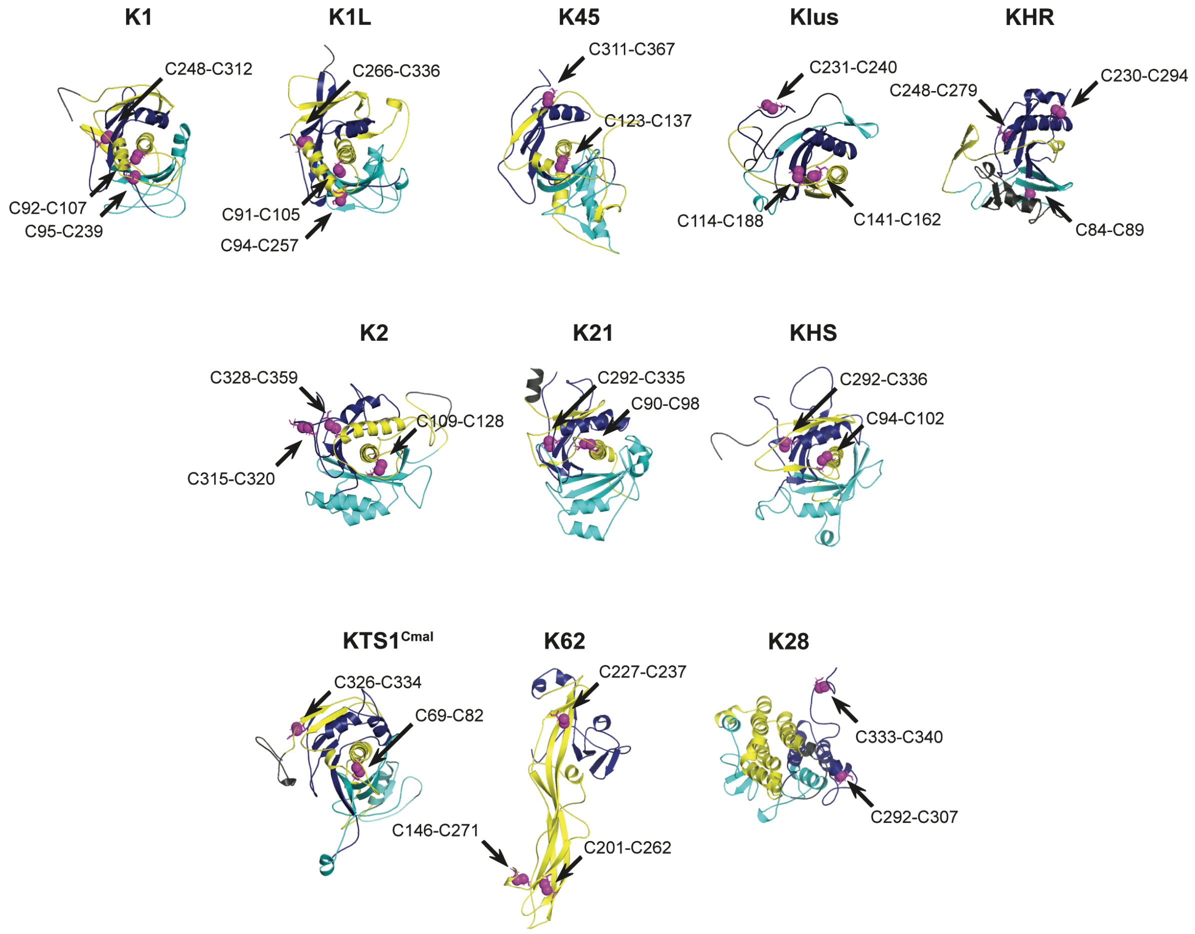

Figure S5. Relative positioning of cysteine pairs in all killer toxin tertiary structure models. Cartoon representations of pTox tertiary structures colored by their delta (black), alpha (yellow), gamma (cyan), and beta domains (dark blue). Cysteine pairs are represented by adjacent magenta spheres and are labelled by amino acid numbers (unpaired cysteine residues are omitted for clarity).

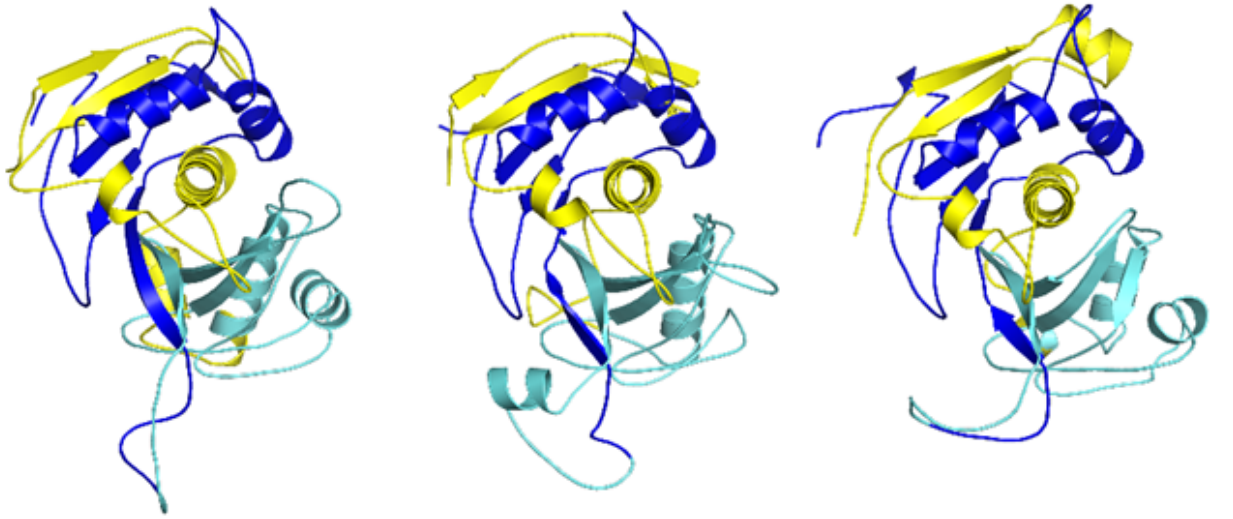

Figure S6. AlphaFold3 predicted structures of representative K74 homologs. Left to right: Accession: KAI6714648.1 from *Diplocarpon mali*, accession: CZR64371.1 from *Phialocephala subalpin*, and accession: OJJ75601.1 from *Aspergillus brasiliensis*. Structures colored by predicted alpha domain (yellow), gamma domain (cyan), and beta domain (blue) based on dibasic motif.

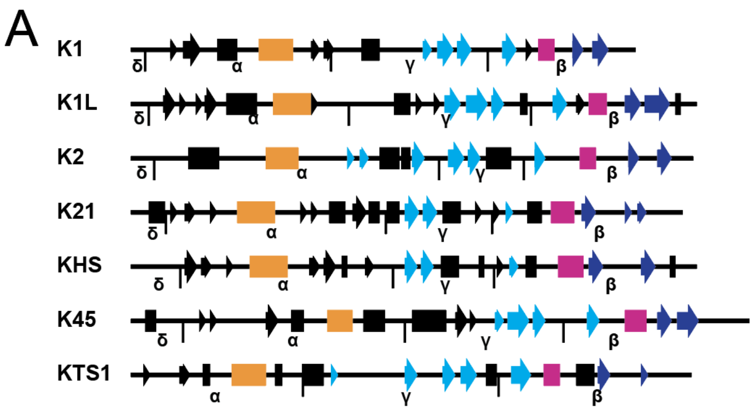

Figure S7. Linear representation of K1 superfamily killer toxins and tertiary structure homologs colored by conserved secondary structure features. Linear representation of proteins representing (A) killer toxins and (B) pilins, pseudopilins, and SMKT. Secondary structure elements that are conserved across structures are colored to highlight similarities including the central hydrophobic helix (orange) and a second conserved helix (magenta) that are positioned before and after beta strands (cyan) ending in C-terminal beta strands (dark blue). Structural features colored black are not considered to be conserved between different proteins. Alpha helices and beta sheets shown as rectangles and arrows, respectively. PDB accession numbers are included after gene names for the pilins, pseudopilins, and SMKT.

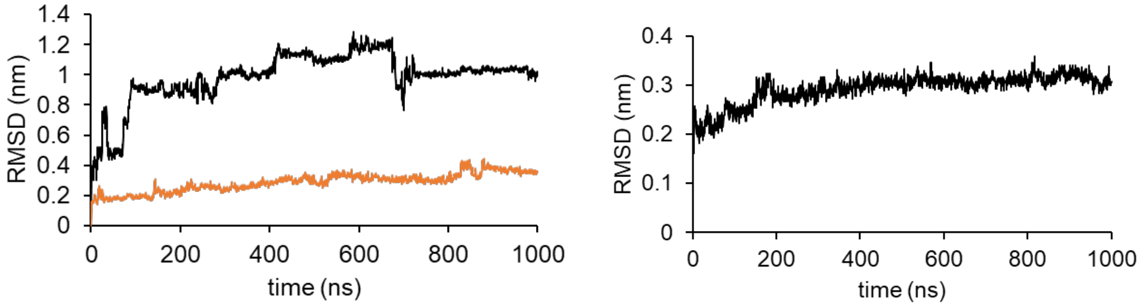

Figure S8. Molecular dynamics simulations of mature Klus and KHS. RMSD of backbone atoms of mature Klus (left) and mature KHR (right) compared to the starting conformation over 1 µs MD simulation. The orange line on Klus is the backbone atom RMSD minus the 30 C-terminal residues of the beta domain.
